# Relationships Between Diet and Geographic Atrophy Progression in the Age-Related Eye Diseases Studies 1 and 2 †

**DOI:** 10.3390/nu17050771

**Published:** 2025-02-22

**Authors:** Elvira Agrón, Emily Vance, Amitha Domalpally, Emily Y. Chew, Tiarnán D. L. Keenan

**Affiliations:** 1 Division of Epidemiology and Clinical Applications, National Eye Institute, National Institutes of Health, Bethesda, MD 20892, USA; 2 Department of Ophthalmology and Visual Sciences, Madison School of Medicine and Public Health, University of Wisconsin, Madison, WI 53726, USA

**Keywords:** age-related macular degeneration, geographic atrophy, Mediterranean diet, micronutrients, Age-Related Eye Diseases Study, diet, oral supplements

## Abstract

**Background/Objectives**: The objective of this study was to analyze the relationships between diet and geographic atrophy (GA) progression, both area-based and proximity-based, for dietary pattern, components, and micronutrients. **Methods**: In the Age-Related Eye Diseases Study (AREDS) and AREDS2, an Alternative Mediterranean Diet Index (aMedi), its nine components, and individual micronutrient intakes were calculated. Mixed-model regression was performed for square root GA area, GA foveal proximity, and acuity. **Results**: The study populations comprised 657 (AREDS) and 1179 eyes (AREDS2). For area-based progression, a higher aMedi was associated with slower progression in AREDS2 and (in analyses excluding MUFA:SFA) AREDS. A higher intake was associated with slower progression for seven components (including vegetables and fruit at Bonferroni) and four components (including fruit and less red meat at Bonferroni), and seven and 15 nutrients, in AREDS1/2, respectively. For proximity-based progression, a higher aMedi was associated with slower progression in AREDS. A higher intake was associated with slower progression for three components (including vegetables at Bonferroni) and two components, and 10 and 8 nutrients, in AREDS1/2, respectively. With increasing oral supplementation, associations between proximity-based progression and aMedi/components/nutrients were weaker. In AREDS2 eyes with non-central GA, higher aMedi was associated with a slower acuity decline. **Conclusions**: A Mediterranean-type diet is associated with slower GA area-based progression and slower progression to the fovea, accompanied by a slower decline in acuity. The most important components and micronutrients for incidence, area-based progression, and foveal progression overlap only partially. For the latter two, they include vegetables, fruit, and less red meat. These findings suggest the benefits of targeted nutritional and supplementation strategies.

## 1. Introduction

Geographic atrophy (GA) represents the atrophic subtype of late age-related macular degeneration (AMD) [1]. Its global prevalence has been estimated at 0.44%, corresponding to approximately five million people affected worldwide [2,3]. Importantly, its prevalence increases markedly with age, from approximately 0.5% (65–79 years) to 4.7% (80–84 years) and 11.3% (90+ years) in populations of European ancestry [4]. In addition to its impact on quality of life, it carries a substantial economic burden for individuals and society [5,6]. GA typically progresses gradually to severe loss of central vision [4,7,8]. No treatment is available to prevent its occurrence or restore vision to affected areas. Multiple clinical trials have tested various therapeutic approaches to slow its progression, but only two drugs have been approved by the United States Food and Drug Administration for this indication [9,10]. In both cases, potential disadvantages include the requirement for dosing by frequent intravitreal injection, risks including increased incidence of neovascular AMD, and high costs [11,12,13]. For these reasons, additional therapeutic approaches to slow GA progression remain a high priority.

The relationship between diet and risk of late AMD, including GA, has been studied in detail [14,15,16,17,18]. However, we are aware of only one study examining relationships between diet and GA progression rate [19]. This previous study analyzed diet at the level of dietary pattern in the Age-Related Eye Diseases Study 2 (AREDS2), using GA area-based progression as the outcome measure. Higher adherence to a Mediterranean-type diet was associated with slower area-based progression.

However, additional analysis of diet at the level of biologically active nutrients may provide further insights into the molecular basis of GA progression and potential preventative strategies. Ideally, this should be performed using a broad and unbiased approach, by analyzing many nutrients in different classes [14]. Dietary analyses should be performed at three levels—pattern, component, and nutrient—to maximize the likelihood of identifying important relationships, since each food component (e.g., vegetables) contains multiple nutrients, while each nutrient (e.g., lutein) can be consumed through different foods.

We have written on the importance of analyzing proximity-based progression, alongside area-based progression, for non-central GA [20]. First, GA progression into the central macula is slower than progression elsewhere, leading to the beneficial phenomenon of foveal sparing [21,22,23]. Second, when GA involves the macular center-point and has sufficient size, it is typically accompanied by severely decreased visual acuity [7,24,25,26,27,28]. Therefore, the time taken for GA to reach the macular center-point is an important metric [21]. Therapeutic approaches that could slow GA progression toward the central macula would be highly valuable. Indeed, our recent findings suggest that oral supplementation with antioxidants and lutein/zeaxanthin leads to slower GA progression to the central macula [20].

This study aimed to extend our previous analyses in three ways: (i) add analyses at the nutrient level to previous analyses at the pattern and component level [19]; (ii) add GA proximity-based progression and change in visual acuity as additional outcome measures, alongside GA area-based progression; and (iii) analyze both AREDS and AREDS2.

## 2. Methods

### 2.1. Study Procedures

The AREDS and AREDS2 designs have been described previously [29,30]. In AREDS, 4757 participants were recruited at 11 US retinal specialty clinics. The eligibility criteria have been described in detail [29]. In brief, participants had to be aged 55–80 years at enrollment and be free of any illness or condition that would make long-term follow-up or compliance with study medications unlikely or difficult. The ocular media had to be sufficiently clear for adequate fundus photographs. At least one eye had to be free from any eye disease that could complicate the assessment of AMD, lens opacity progression, or visual acuity. Study eyes could not have had previous ocular surgery (other than cataract surgery). Of the 4757 participants, 3640 participants with AMD were randomized in a 2 × 2 factorial design to the following: antioxidants (500 mg vitamin C; 400 IU vitamin E; 15 mg β-carotene), 80 mg zinc, antioxidants plus zinc, or placebo. The randomized trial lasted five years, followed by epidemiologic follow-up in 3549 of the 4203 surviving participants. All participants were offered the supplements during epidemiologic follow-up.

In AREDS2, 4203 participants were recruited at 82 US retinal specialty clinics [30]. Again, the eligibility criteria have been described in detail [30]. Participants had to be aged 50–85 years at enrollment; be able and willing to consent to the study and undergo yearly examinations for at least five years; to agree to stop oral supplements other than those supplied by AREDS2; demonstrate adherence to the run-in regimen; and have fundus photographs of adequate quality. The exclusion criteria included the following: ocular disease in either eye that could confound retinal evaluation; previous ocular surgical procedures (other than cataract extraction) that might complicate the assessment of AMD progression; chronic requirement for any medication known to be toxic to the retina or optic nerve; ocular hypertension or glaucoma; or cataract surgery within three months or capsulotomy within six weeks before the qualification visit. The AMD-specific eligibility criteria were either large drusen in both eyes or late AMD in one eye and large drusen in the fellow eye. The participants were randomized in a 2 × 2 factorial design to the following: lutein plus zeaxanthin (10 mg/2 mg), docosahexaenoic acid (DHA) plus eicosapentaenoic acid (EPA) (350 mg/650 mg), lutein/zeaxanthin and DHA/EPA, or placebo. All participants were also offered the original AREDS formulation. The clinical trial lasted five years.

In both studies, at baseline and annual follow-up visits, best-corrected visual acuity (BCVA) was measured using Early Treatment Diabetic Retinopathy Study (ETDRS) charts, eye examinations were performed, and stereoscopic color fundus photographs were captured and graded at the Wisconsin Reading Center [31]. The participants, investigators, and reading center personnel were masked to the treatment assignments.

Institutional review board approval was obtained at each site and written informed consent obtained from all participants. The research was conducted under the tenets of the Declaration of Helsinki and, for AREDS2, complied with the Health Insurance Portability and Accountability Act. The first AREDS participant was enrolled in November 1992 and the last study visit of the last participant for the primary outcome was in April 2001. The trial was registered at ClinicalTrials.gov (https://clinicaltrials.gov/study/NCT00000145, accessed on 7 January 2025) in September 1999, soon after ClinicalTrials.gov was launched. The AREDS2 was registered at ClinicalTrials.gov (https://clinicaltrials.gov/study/NCT00345176, accessed on 7 January 2025) in June 2006. The first participant was enrolled in October 2006 and the last study visit of the last participant for the primary outcome was in October 2012.

### 2.2. Evaluation of Geographic Atrophy on Fundus Photographs

The GA grading definitions and methods have been described previously [7,28,32]. In both studies, planimetry tools were used to measure the GA area within the AREDS grid and GA proximity to the foveal center-point [7,28,31].

### 2.3. Modified Alternative Mediterranean Diet Index

The assessment of the modified Alternative Mediterranean Dietary Index (aMedi) in AREDS/AREDS2 has been described previously [33]. In brief, in both studies, food frequency questionnaires (FFQs) were administered to all participants at randomization. The AREDS FFQ, a 90-item, semi-quantitative modified Block FFQ, and its validation, have been described previously [34]. The AREDS2 FFQ, a 131-item, semi-quantitative Harvard FFQ, and its validation, have also been described previously [35,36]. In both FFQs, participants were asked how often, on average, they had consumed each food/beverage item during the preceding year. The FFQs were used to determine the number of medium-sized servings of each food item consumed per week (or gram/day for alcohol). These data were summed for each participant to obtain the intake for each of the nine components that define adherence to the Mediterranean diet: whole fruits, vegetables, whole grains, nuts, legumes, red meat, fish, monounsaturated fatty acid–saturated fatty acid ratio (MUFA:SFA), and alcohol. For each component, sex-specific intake quartiles (1–4) were calculated, with quartile 4 representing the highest intake. Alcohol intake was converted into binary format: 4 for intake within the specified intervals (5–15 g/day (female) or 10–15 g/day (male)), and 0 for intake above or below the specified intervals [37]. The quartiles for red meat were reversed (i.e., highest intake scored 1, as least aMedi-adherent, and lowest intake scored 4). To calculate the aMedi score for each participant, the quartile values for the nine components were summed (range 8–36).

The FFQ data were also used to estimate, for each participant, the daily dietary intake of 38 nutrients (AREDS) or 44 nutrients (AREDS2). For each nutrient, intake was divided by total calorie intake to represent nutrient intake density and intake tertiles were calculated (separately for each cohort and for men/women), with tertile 3 representing highest intake.

### 2.4. Study Populations

All eyes with GA measurements available at two or more study visits (without previous or simultaneous neovascular AMD) were eligible. In the proximity-based analyses, the study population was restricted to eyes with non-central GA.

### 2.5. Statistical Methods: Exposure Variables

Three sets of exposure variables were used in separate analyses: (i) aMedi (considered in tertiles), (ii) nine aMedi components (considered in quartiles), and (iii) nutrient intake (considered in tertiles). For the nine aMedi components, a separate model was made for each component (component x), adjusting for the total aMedi modified to exclude that component (modified aMedi = total aMedi—component x), to isolate the contribution of each component from that of the overall diet pattern [33,38].

### 2.6. Statistical Methods: Outcome Measures

The two co-primary outcome measures were (i) rate of change per year in square root of the GA area and (ii) rate of change per year in GA proximity to the central macula. The analyses were performed using mixed-model repeated-measures regression, employing methods similar to those described previously, with the eye as the unit of analysis [7,19,20]. For the GA area, the square root transformation was used to reduce the dependence of the area-based progression rate on baseline lesion size [7,39,40,41,42,43,44]. Further details are provided in the Supplementary Materials.

In supplementary analyses, the rate of change in BCVA was analyzed by mixed-model repeated-measure regression in each of the study populations, using similar methods (Supplementary Materials).

To explore whether oral supplement use influenced the relationships between diet and GA progression, the proximity analyses were repeated separately for those participants randomized to (i) antioxidants vs. no antioxidants (AREDS), while limiting follow-up to the randomized trial, and (ii) lutein/zeaxanthin vs. no lutein/zeaxanthin (AREDS2). This was performed because recent analyses have shown that the AREDS participants randomized to the antioxidants, and the AREDS2 participants randomized to lutein/zeaxanthin, had significantly slower proximity-based progression, though those analyses did not evaluate dietary intake alongside the oral supplement randomizations [20].

All analyses were performed with commercially available statistical software (SAS version 9.4; SAS Institute, Cary, NC, USA). The Bonferroni level of significance was set at *p* = 0.0005 for the AREDS analyses (based on 48 dietary exposure variables and two main outcome measures) and at *p* = 0.0005 for the AREDS2 analyses (based on 54 dietary exposure variables and two main outcome measures). We considered that *p*-values higher than these levels could be due to chance, but did not disregard these associations, as higher *p*-values could also reflect how well the dietary intake of those nutrients was captured on FFQs and the limitations of nutrient databases, rather than their physiological importance.

## 3. Results

### 3.1. Geographic Atrophy Area-Based Progression Rate in AREDS

The study population for these analyses comprised 657 eyes of 508 participants. Their demographic and clinical characteristics are shown in Table 1 and their dietary characteristics in Table S1. The mean follow-up was 5.0 years.

**Figure 1:**
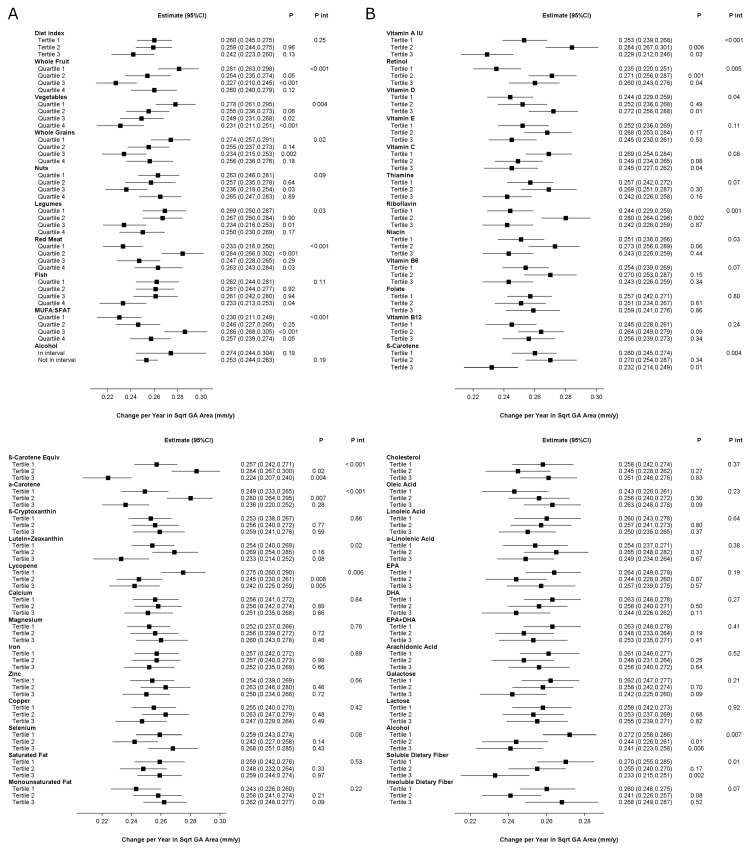
Geographic atrophy area-based progression rates, according to the quantiles of dietary index or intake in the Age-Related Eye Diseases Study: (**A**) Alternative Mediterranean Diet Index and its nine components; (**B**) nutrients. Higher quantiles indicate higher adherence to the Alternative Mediterranean Diet, higher intake of its components, or higher intake of individual nutrients. The exceptions are red meat, where higher quantiles indicate lower intake, i.e., higher adherence to the Alternative Mediterranean Diet, and alcohol, where “in interval” refers to intake within the specified interval. Abbreviations: CI = confidence interval; DHA = docosahexaenoic acid; EPA = eicosapentaenoic acid; GA = geographic atrophy; IU = international units; MUFA:SFAT = monounsaturated fatty acid–saturated fatty acid ratio.

The results of the analyses are shown in Figure 1, and Tables S2 and S3. A higher aMedi was not significantly associated with an altered GA area-based progression rate at the nominal level. However, for two of the aMedi components analyzed individually, a higher intake was associated with slower area-based progression at the Bonferroni level: whole fruit and vegetables. For five additional components, it was associated at the nominal level. Paradoxically, for one component (MUFA:SFA), a higher quartile was associated with faster progression. In the analyses of a modified aMedi that excluded MUFA:SFA, a higher aMedi tertile was associated with slower area-based progression at the nominal level (*p* = 0.037), with estimates of 0.269 mm/year (95% confidence interval [CI] 0.255–0.283), 0.249 mm/year (95% CI 0.233–0.264, *p* = 0.055), and 0.240 mm/year (95% CI 0.220–0.260, *p* = 0.020) for tertiles 1–3, respectively.

No nutrient was associated with slower or faster area-based progression at the Bonferroni level. For seven of the 38 nutrients, association with slower progression was present at the nominal level. For four nutrients, association with faster progression was present at the nominal level.

The results of similar analyses in the proximity study population (i.e., only eyes with non-central GA) are shown in Figure S1 and Tables S4 and S5. A higher aMedi was significantly associated with slower area-based progression at the nominal level. The estimates were 0.296 mm/year (95% CI 0.275–0.316), 0.284 mm/year (95% CI 0.262–0.307, *p* = 0.47), and 0.259 mm/year (95% CI 0.233–0.285, *p* = 0.030), for tertiles 1–3, respectively.

### 3.2. Geographic Atrophy Area-Based Progression Rate in AREDS2

**Figure 2:**
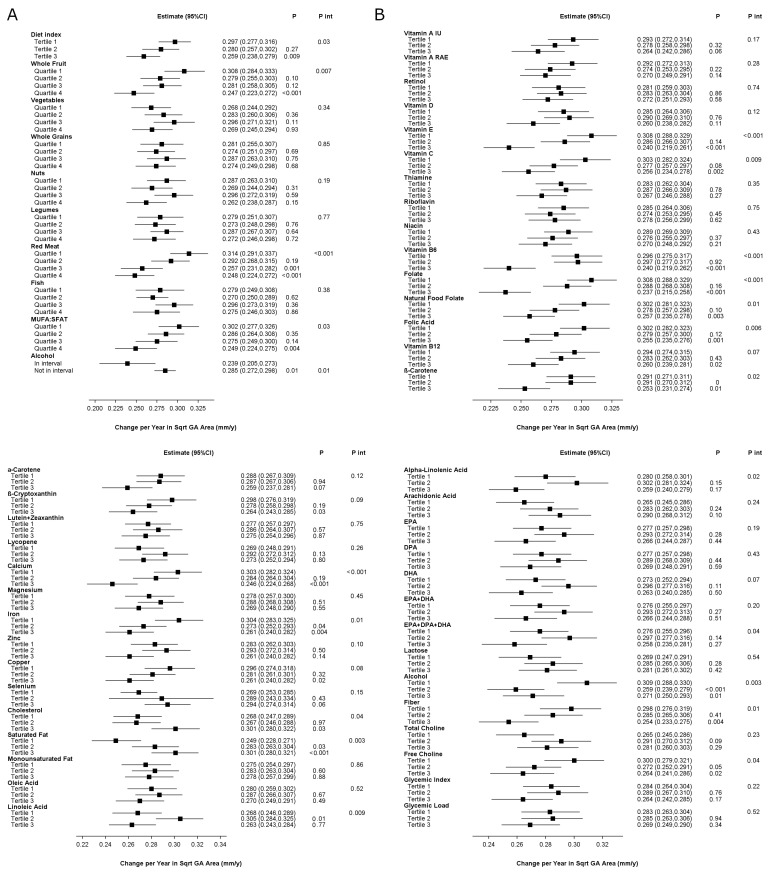
Geographic atrophy area-based progression rates, according to quantiles of dietary index or intake in the Age-Related Eye Diseases Study 2: (**A**) Alternative Mediterranean Diet Index and its nine components; (**B**) nutrients. Higher quantiles indicate higher adherence to the Alternative Mediterranean Diet, higher intake of its components, or higher intake of individual nutrients. The exceptions are red meat, where higher quantiles indicate lower intake, i.e., higher adherence to the Alternative Mediterranean Diet, and alcohol, where “in interval” refers to intake within the specified interval. Abbreviations: CI = confidence interval; DHA = docosahexaenoic acid; DPA = docosapentaenoic acid; EPA = eicosapentaenoic acid; GA = geographic atrophy; IU = international units; MUFA:SFAT = monounsaturated fatty acid: saturated fatty acid ratio; RAE = retinal activity equivalents.

The study population for these analyses comprised 1179 eyes of 867 participants. The mean follow-up was 3.1 years. The results of the analyses are shown in Figure 2, and Tables S2 and S3. A higher aMedi was significantly associated with slower progression at the nominal level. The estimates were 0.297 mm/year (95% CI 0.277–0.316), 0.280 mm/year (95% CI 0.257–0.302, *p* = 0.27), and 0.259 mm/year (95% CI 0.238–0.279, *p* = 0.010), for tertiles 1–3, respectively. For two components, association with a slower progression was present at the Bonferroni level: whole fruit and red meat. For two additional components, it was present at the nominal level.

For four of the 44 nutrients, a higher intake was associated with slower progression at the Bonferroni level. For 11 additional nutrients, association was present at the nominal level. For three nutrients, association with faster progression was present at the nominal level.

The results in the proximity study population are shown in Figure S2 and Tables S4 and S5. Again, a higher aMedi was associated with a slower area-based progression at the nominal level.

### 3.3. Geographic Atrophy Progression Toward Central Macula in AREDS

**Figure 3:**
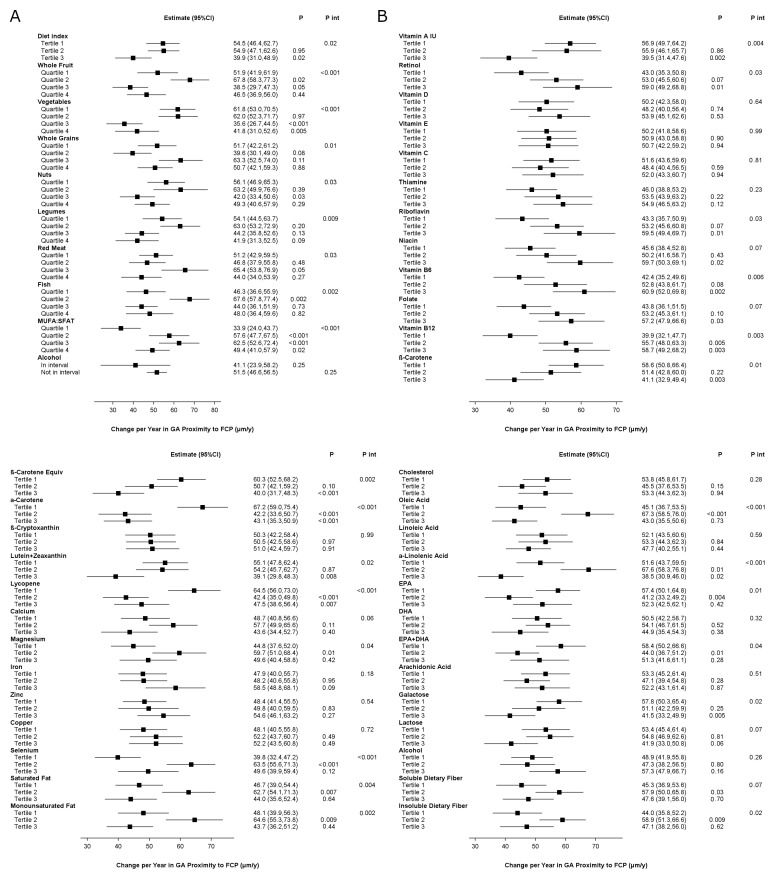
Geographic atrophy proximity-based progression rates, according to quantiles of dietary index or intake in the Age-Related Eye Diseases Study: (**A**) Alternative Mediterranean Diet Index and its nine components; (**B**) nutrients. Higher quantiles indicate higher adherence to the Alternative Mediterranean Diet, higher intake of its components, or higher intake of individual nutrients. The exceptions are red meat, where higher quantiles indicate lower intake, i.e., higher adherence to the Alternative Mediterranean Diet, and alcohol, where “in interval” refers to intake within the specified interval. Abbreviations: CI = confidence interval; DHA = docosahexaenoic acid; EPA = eicosapentaenoic acid; GA = geographic atrophy; IU = international units; MUFA:SFAT = monounsaturated fatty acid–saturated fatty acid ratio.

The study population for these analyses comprised 390 eyes of 328 participants. The mean follow-up was 3.5 years. The results of the analyses are shown in Figure 3, and Tables S6 and S7. A higher aMedi was significantly associated with slower proximity-based progression at the nominal level. For one component, higher intake was associated with slower progression at the Bonferroni level: vegetables. For two additional components, association was present at the nominal level. Conversely, for one component (MUFA:SFA), higher intake was associated with faster progression at the Bonferroni level.

For three nutrients, higher intake was associated with slower progression at the Bonferroni level. For seven additional nutrients, it was present at the nominal level. Conversely, for two nutrients, higher intake was associated with faster progression at the Bonferroni level. For 11 additional nutrients, association with faster progression was present at the nominal level.

In participants randomized to no antioxidants, a higher aMedi was associated with slower progression at the nominal level (Figure S3 and Tables S8 and S9). Associations with slower progression were present for six components (three at Bonferroni and three at nominal). By contrast, in participants randomized to antioxidants, a higher aMedi was not associated with slower progression. Associations with slower progression were present for only two components (nominal only).

### 3.4. Geographic Atrophy Progression Toward Central Macula in AREDS2

**Figure 4:**
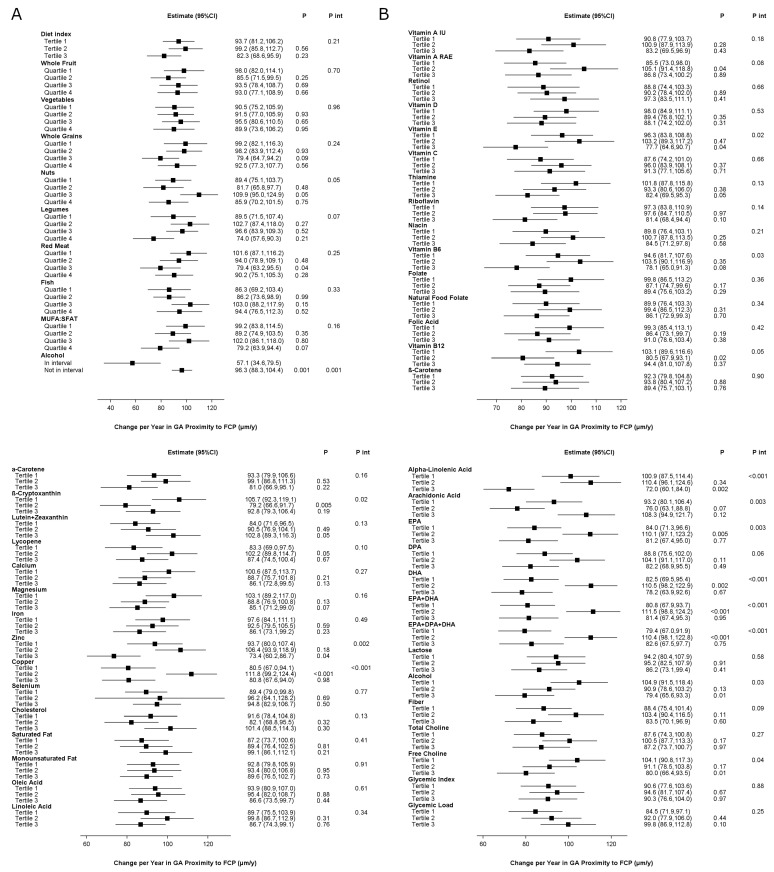
Geographic atrophy proximity-based progression rates, according to quantiles of dietary index or intake in the Age-Related Eye Diseases Study 2: (**A**) Alternative Mediterranean Diet Index and its nine components; (**B**) nutrients. Higher quantiles indicate higher adherence to the Alternative Mediterranean Diet, higher intake of its components, or higher intake of individual nutrients. The exceptions are red meat, where higher quantiles indicate lower intake, i.e., higher adherence to the Alternative Mediterranean Diet, and alcohol, where “in interval” refers to intake within the specified interval. Abbreviations: CI = confidence interval; DHA = docosahexaenoic acid; DPA = docosapentaenoic acid; EPA = eicosapentaenoic acid; GA = geographic atrophy; IU = international units; MUFA:SFAT = monounsaturated fatty acid: saturated fatty acid ratio; RAE = retinal activity equivalents.

The study population for these analyses comprised 826 eyes of 652 participants. Mean follow-up was 2.4 years. The results are shown in Figure 4 and Tables S6 and S7. Higher aMedi was not associated with slower progression at the nominal level. For two components, a higher quartile was associated with slower progression at the nominal level: red meat and alcohol.

For eight nutrients, an association with slower progression was present at the nominal level. For seven nutrients, an association with faster progression was present at the nominal level.

In participants randomized to no lutein/zeaxanthin, a higher aMedi had a borderline *p*-value for association with slower progression (Figure S4 and Tables S8 and S9). Associations with a slower progression were present for four components (one at Bonferroni and three at nominal). By contrast, in participants randomized to lutein/zeaxanthin, a higher aMedi was not associated with slower progression. An association with slower progression was present for only one component.

### 3.5. Rate of Change in Visual Acuity in AREDS1/2

The results of the analyses of change in visual acuity are shown in Figures S5–S8 and Tables S10–S13. In the AREDS area study population, a higher aMedi was not associated with an altered rate of BCVA decline at the nominal level. For one nutrient, higher intake was associated with a slower decline at the Bonferroni level: vitamin C. For four additional nutrients, association was present at the nominal level. Conversely, for eight nutrients, higher intake was associated with faster decline at the nominal level.

In the AREDS2 area study population, a higher aMedi was not associated with a slower BCVA decline at the nominal level. For one component, higher quartile was associated with a slower decline at the Bonferroni level: red meat. For three additional components, an association with a slower decline was present at the nominal level. For seven nutrients, a higher tertile was associated with a slower rate of decline at the Bonferroni level. For 15 additional nutrients, an association was present at the nominal level. Conversely, for one nutrient, a higher intake was associated with a faster decline at the nominal level: saturated fat.

In the AREDS2 proximity study population, a higher aMedi was associated with slower BCVA decline at the nominal level. For two components, a higher quartile was associated with a slower BCVA decline at the Bonferroni level: whole fruit and red meat.

## 4. Discussion

### 4.1. Main Findings and Clinical Implications

Overall, the findings were consistent with higher adherence to a Mediterranean-type diet being associated with slower GA area-based progression. Those components contributing most strongly were fruit, vegetables, and less red meat. At the nutrient level, the strongest protective associations were for some carotenoids (particularly beta-carotene), some vitamins (e.g., vitamins A, B6, B12, folate, C, and E), and some minerals (e.g., calcium), as well as fiber. The findings were also consistent with a Mediterranean-type diet being associated with slower proximity-based progression, i.e., greater tendency toward foveal sparing. Again, the association for vegetables was particularly strong, with beta-carotene as the most important nutrient. Some strong associations were observed even for change in visual acuity. The magnitude of the associations may be clinically meaningful. For example, the AREDS2 GA progression estimates for aMedi tertiles one vs. three were approximately 15% different for square root area and 32% different for proximity. Even for individual components, the equivalent estimates were 22% different for fruit (AREDS1/2 area) and 53% different for vegetables (AREDS proximity).

In AREDS, area-based progression was numerically slower with a higher aMedi. This was not statistically significant, despite the large majority of the constituent components having protective associations, because of the paradoxical MUFA:SFA results. Indeed, aMedi analyses excluding this component demonstrated a significant association. Higher MUFA:SFA is usually associated with health benefits [37,45]; its association here likely relates to it capturing higher animal fat intake, as opposed to higher plant-based oil intake, which it was designed to capture in Mediterranean populations [37,45]. In component analyses, vegetable and fruit intake each appeared highly important. Nutrients notable for protective associations included vitamins with antioxidant properties (vitamins A and C), carotenoids (beta-carotene and lycopene), and soluble fiber. This is consistent with the component analyses, since these nutrients are abundant in fruit and vegetables.

In similar AREDS2 analyses, the aMedi results did meet statistical significance. Consistent with the AREDS results, higher fruit and lower red meat intake each appeared highly important. Nutrients with protective associations included vitamins and carotenoids with antioxidant properties (particularly vitamin C, vitamin E, and beta-carotene, which comprise the AREDS/AREDS2 antioxidant formulation), together with some B vitamins, minerals, and fiber. SFA was strongly associated with faster progression. Overall, both the AREDS and AREDS2 results suggest the importance of plant-based vs. animal-based diets, with many plant-based components and nutrients associated with slower GA progression and many animal-based ones with faster progression.

In the AREDS analyses, a higher aMedi was associated with slower proximity-based progression. The component contributing most strongly was vegetables, followed by fruit and nuts. Hence, the findings for proximity-based vs. area-based progression overlapped, with vegetable and fruit intake relevant for both. However, more components had associations with area-based than proximity-based progression. For direct comparison, we performed both analyses in the same study population. Overall, the results point to the partially overlapping but partially distinct nature of GA progression near the fovea vs. elsewhere [21,22,46]. At the nutrient level, those with the strongest protective associations were carotenoids. Interestingly, non-linear associations were suggested for multiple lipids. Aside from genuine non-linear relationships, one likely explanation is animal vs. plant-based sources.

In the AREDS2 analyses, a higher aMedi was not associated with slower proximity-based progression. Despite vegetable and fruit intake being strongly associated with slower proximity-based progression in AREDS, neither was associated in AREDS2. Similarly, the number of nutrients with strong protective associations was low in AREDS2. The likely explanation is the high degree of oral supplementation in AREDS2. This question was therefore addressed in the dedicated analyses of potential interactions between supplementation vs. dietary intake, i.e., potential redundancy vs. complementarity, for proximity-based progression. Overall, the number and strength of associations with dietary intake appeared to decrease in the following order, from highest to lowest: (i) AREDS participants randomized to no antioxidants (i.e., lowest supplementation); (ii) AREDS participants randomized to antioxidants and AREDS2 participants randomized to no lutein/zeaxanthin; and (iii) AREDS2 participants randomized to lutein/zeaxanthin (i.e., highest supplementation). These results point to some degree of redundancy, though only partial redundancy, between supplements and diet. This might suggest that, for individuals with non-central GA, either adopting a healthy diet or taking antioxidant and lutein/zeaxanthin supplements may slow proximity-based progression. However, the AREDS1/2 cohorts tended to have much healthier diets, compared to the US population [47], so much less redundancy might be expected in the general population. Hence, pursuing both approaches is likely to have additional benefits beyond either one alone.

We have previously discussed potential mechanisms responsible for the associations between a Mediterranean-type diet and slower GA progression [19,48]. These may include the following properties of the diet pattern: anti-inflammatory, anti-oxidative, altered lipid metabolism, vascular protection, improved mitochondrial energetics, and neural protection [49]. For example, the protective associations for fruit and vegetable intake might be mediated by flavonoids and other antioxidants, which are thought to decrease oxidative stress and enhance vascular health, while the harmful association for red meat might relate to altered lipid metabolism and increased inflammation [50,51]. More broadly, chronic local inflammation is implicated in GA progression, and many aMedi-related components and nutrients are known to decrease systemic inflammation [49]. In addition, some of these are thought to improve vascular health, so might slow GA progression through effects on the choriocapillaris [52,53,54]. Other potential mechanisms include those mediated by alterations in the gut microbiome [55].

These findings demonstrate that the dietary factors associated with GA risk overlap, but only partially, with those for GA progression [48]. By comparison with our previous AREDS/AREDS2 analyses [14,33], a higher aMedi is associated with both decreased GA risk and slower GA progression. However, the components chiefly responsible appear to differ. Fish has the strongest protective association with GA risk but is weakly associated with GA progression. Vegetables and fruit, and less red meat have the strongest protective associations with GA progression but are weakly associated with GA risk. Similarly, in the nutrient analyses, higher or lower intakes of different lipids have the strongest protective associations with GA risk but have weaker associations with GA progression. By contrast, higher intakes of multiple vitamins and carotenoids have the strongest protective associations with GA progression but are less associated with GA risk. Overall, a Mediterranean-like dietary pattern is strongly associated with both slower progression to GA and slower GA progression, but for partially distinct reasons [48].

### 4.2. Findings for Visual Acuity

We had modest expectations that associations with slower GA progression would be accompanied by slower BCVA decline, over these follow-up times, for reasons discussed previously [20,56,57,58,59]. Despite this, corresponding results were observed for some components and nutrients, particularly in AREDS2, where power was greater. For example, in the AREDS2 proximity study population, a higher aMedi was associated with a slower BCVA decline, with strong individual associations for fruit and lower red meat intake, as well as for several nutrients, such as vitamin E. Even in the AREDS2 area study population, strong associations were seen for some components, such as red meat, and for multiple nutrients. These results strengthen those of the structural analyses. They suggest that the Mediterranean diet is associated with a slower decline in acuity, particularly for non-central GA. This is especially true for some of its components and nutrients, such as more fruit, healthier lipids, and less red meat.

### 4.3. Comparison with the Literature

We are not aware of any studies examining the relationships between diet and GA progression, aside from one from our group, which analyzed at the pattern/component levels only, in AREDS2 only [19].

### 4.4. Strengths and Limitations

Strengths include the analysis of three outcome measures (comprising structural and functional measures), three different and complementary dietary levels (for a broad and unbiased approach), and two independent datasets (towards replication of some findings and an understanding of reasons for partially distinct findings). Additional strengths include the large study populations, clinical trial setting, reading center grading, and detailed dietary information by validated FFQ. Potential limitations include post hoc hypothesis generation and the possibility of residual or unmeasured confounding (e.g., related to physical activity, as data on physical activity were not available in the AREDS1/2). Diet assessment by FFQ contains non-differential measurement error, though energy adjustment may partially address this [60,61]. Generalizability to populations of different racial or dietary characteristics is unknown.

## 5. Conclusions

In this comprehensive interrogation of the potential relationships between diet and GA progression, a Mediterranean-type diet was associated with slower area-based progression. Specific components and micronutrients appear particularly relevant, in a pattern that differs partially from GA risk. The most important components include vegetables, fruit, and less red meat, i.e., aspects of a plant-based diet. A Mediterranean-type diet was also associated with slower GA progression to the central macula. In the most highly powered analyses of eyes with non-central GA, a Mediterranean-type diet was also associated with a slower decline in visual acuity. These findings may suggest the potential benefits of targeted nutritional and supplementation strategies for individuals at different AMD stages. Future research directions in the field may include analyses of the relationship between diet, oral supplementation, other lifestyle factors and GA progression, ideally incorporating data from metabolomics/multi-omics and the intestinal microbiome. They may also include potential interventional clinical trials in GA, based on oral supplementation or lifestyle/diet modification.

## Figures and Tables

**Table 1:** Demographic and clinical characteristics of the study populations at the first time-point with geographic atrophy in the Age-Related Eye Diseases Studies 1 and 2.

		AREDS		AREDS2	
		GA Area Study Population	GA Proximity Study Population	GA Area Study Population	GA Proximity Study Population
Participants: n		508	328	867	652
Age: mean (SD)		70.5 (5.4)	70.9 (5.3)	75.0 (6.8)	74.8 (6.9)
Sex: n (%)	Female	281 (55.3)	196 (59.8)	501 (57.8)	386 (59.2)
	Male	227 (44.7)	132 (40.2)	366 (42.2)	266 (40.8)
Smoking status: n (%)	Never	203 (40.0)	134 (40.9)	352 (40.6)	265 (40.6)
	Former	255 (50.2)	165 (50.3)	458 (52.8)	347 (53.2)
	Current	50 (9.8)	29 (8.8)	57 (6.6)	40 (6.1)
Follow-up time (years): mean (SD)		5.0 (3.1)	3.5 (2.3)	3.1 (1.5)	2.4 (1.4)
Randomized oral supplementation assignment *	1	114 (22.4)	64 (19.5)	226 (26.1)	163 (25.0)
	2	110 (21.7)	75 (22.9)	194 (22.4)	142 (21.8)
	3	138 (27.2)	100 (30.5)	222 (25.6)	171 (26.2)
	4	146 (28.7)	89 (27.1)	225 (26.0)	176 (27.0)
Eyes: n		657	390	1179	826
GA cohort: n (%)	Prevalent	159 (24.2)	81 (20.8)	444 (37.7)	515 (62.3)
	Incident	498 (75.8)	309 (79.2)	735 (62.3)	311 (37.7)
GA configuration: n (%)	Missing	187 (28.5)	97 (24.9)	0 (0)	0 (0)
	Small (single patch < 1 DA)	262 (39.9)	171 (43.8)	587 (49.8)	430 (52.1)
	Multifocal	102 (15.5)	83 (21.3)	274 (23.2)	246 (29.8)
	Horseshoe, Ring	15 (2.3)	13 (3.3)	57 (4.8)	54 (6.5)
	Solid (center or not)	80 (12.2)	17 (4.4)	220 (18.7)	70 (8.5)
	Indeterminate	11 (1.7)	9 (2.3)	41 (3.5)	26 (3.1)
Central GA	No	392 (59.7)	.	854 (72.4)	.
	Yes	265 (40.3)	.	325 (27.6)	.
GA area (mm^2^): mean (SD)		3.3 (5.1)	2.39 (3.92)	2.4 (3.4)	2.0 (3.1)
GA proximity to central macula (µm): mean (SD)		285.7 (394.0)	480.7 (409.7)	433.3 (499.5)	596.0 (497.0)

Abbreviations: AREDS = Age-Related Eye Diseases Study; DA = disc area; GA = geographic atrophy; SD = standard deviation. * Randomized oral supplementation assignment: 1. placebo (AREDS); control (AREDS2); 2. antioxidants (AREDS); lutein/zeaxanthin (AREDS2); 3. zinc (AREDS); DHA/EPA (AREDS2); 4. antioxidants and zinc (AREDS); lutein/zeaxanthin and DHA/EPA (AREDS2).

## Data Availability

The data presented in this study are available on request from the corresponding author due to privacy and legal reasons.

## Supplementary Materials

The following supporting information can be downloaded at: https://www.mdpi.com/article/10.3390/nu17050771/s1, Table S1: Estimates of Daily Nutrient Intake from Foods in the Study Populations; Table S2: Geographic Atrophy Area-Based Progression Rates, according to Quantiles of the Alternative Mediterranean Dietary Index and its Components, in the Age-Related Eye Diseases Studies 1 and 2; Table S3: Geographic Atrophy Area-Based Progression Rates, according to Quantiles of Nutrient Intake, in the Age-Related Eye Diseases Studies 1 and 2; Table S4: Geographic Atrophy Area-Based Progression Rates, according to Quantiles of the Alternative Mediterranean Dietary Index and its Components, in the Geographic Atrophy Proximity Study Populations of the Age-Related Eye Diseases Studies 1 and 2; Table S5: Geographic Atrophy Area-Based Progression Rates, according to Quantiles of Nutrient Intake, in the Geographic Atrophy Proximity Study Populations of the Age-Related Eye Diseases Studies 1 and 2; Table S6: Geographic Atrophy Proximity-Based Progression Rates, according to Quantiles of the Alternative Mediterranean Dietary Index and its Components, in the Age-Related Eye Diseases Studies 1 and 2; Table S7: Geographic Atrophy Proximity-Based Progression Rates, according to Quantiles of Nutrient Intake, in the Age-Related Eye Diseases Studies 1 and 2; Table S8: Geographic Atrophy Proximity-Based Progression Rates, according to Quantiles of the Alternative Mediterranean Dietary Index and its Components, separately according to Randomized Oral Supplement Assignments, in the Age-Related Eye Diseases Studies 1 and 2; Table S9: Geographic Atrophy Proximity-Based Progression Rates, according to Quantiles of Nutrient Intake, separately according to Randomized Oral Supplement Assignments, in the Age-Related Eye Diseases Studies 1 and 2; Table S10: Rates of Decline in Best-Corrected Visual Acuity, according to Quantiles of the Alternative Mediterranean Dietary Index and its Components, in the Age-Related Eye Diseases Studies 1 and 2; Table S11: Rates of Decline in Best-Corrected Visual Acuity, according to Quantiles of Nutrient Intake, in the Age-Related Eye Diseases Studies 1 and 2; Table S12: Rates of Decline in Best-Corrected Visual Acuity, according to Quantiles of the Alternative Mediterranean Dietary Index and its Components, in the Proximity Study Population of the Age-Related Eye Diseases Studies 1 and 2; Table S13: Rates of Decline in Best-Corrected Visual Acuity, according to Quantiles of Nutrient Intake, in the Proximity Study Population of the Age-Related Eye Diseases Studies 1 and 2; Figure S1: Geographic Atrophy Area-Based Progression Rates, according to Quantiles of Dietary Index or Intake, in the Proximity Study Population of the Age-Related Eye Diseases Study: (A) Alternative Mediterranean Diet Index and its Nine Components; (B) Nutrients; Figure S2: Geographic Atrophy Area-Based Progression Rates, according to Quantiles of Dietary Index or Intake, in the Proximity Study Population of the Age-Related Eye Diseases Study 2: (A) Alternative Mediterranean Diet Index and its Nine Components; (B) Nutrients; Figure S3: Geographic atrophy proximity-based progression rates, according to quantiles of dietary index or intake for the Alternative Mediterranean Diet Index and its nine components, in the Age-Related Eye Diseases Study: (A) in participants randomized to oral antioxidant supplementation; (B) in participants randomized to no oral antioxidant supplementation; Figure S4: Geographic atrophy proximity-based progression rates, according to quantiles of dietary index or intake for the Alternative Mediterranean Diet Index and its nine components, in the Age-Related Eye Diseases Study 2: (A) in participants randomized to oral lutein/zeaxanthin supplementation; (B) in participants randomized to no oral lutein/zeaxanthin supplementation; Figure S5: Rates of Decline in Best-Corrected Visual Acuity, according to Quantiles of Dietary Index or Intake, in the Age-Related Eye Diseases Study: (A) Alternative Mediterranean Diet Index and its Nine Components; (B) Nutrients; Figure S6: Rates of Decline in Best-Corrected Visual Acuity, according to Quantiles of Dietary Index or Intake, in the Age-Related Eye Diseases Study 2: (A) Alternative Mediterranean Diet Index and its Nine Components; (B) Nutrients; Figure S7: Rates of Decline in Best-Corrected Visual Acuity, according to Quantiles of Dietary Index or Intake, in the Proximity Study Population of the Age-Related Eye Diseases Study: (A) Alternative Mediterranean Diet Index and its Nine Components; (B) Nutrients; Figure S8: Rates of Decline in Best-Corrected Visual Acuity, according to Quantiles of Dietary Index or Intake, in the Proximity Study Population of the Age-Related Eye Diseases Study 2: (A) Alternative Mediterranean Diet Index and its Nine Components; (B) Nutrients. References [62,63,64] are cited in Supplementary Materials.
