# Supplementary material for: Relationships Between Diet and Geographic Atrophy Progression in the Age-Related Eye Diseases Studies 1 and 2
Source: Nutrients. 2025 Feb 22;17(5):771. doi: 10.3390/nu17050771 (PMC11901604; doi:10.3390/nu17050771)
Supplement: Supplementary file 1 [file nutrients-17-00771-s001.zip › AREDS Research Group.pdf]

AREDS Research Group:

The Eye Center at Memorial, Albany, NY: Principal Investigator: Aaron Kassoff, MD; Co-Investigator: Jordan Kassoff, MD; Clinic Coordinators: JoAnne Buehler; Mary Eglow, RN; Francine Kaufman; Photographer: Michel Mehu; Past Participating Personnel: Co-Investigator: Shalom Kieval, MD; Examiner: Michael Mairs, MD; Photographers: Barbara Graig; Andrea Quattrocchi; Technicians:Denise Jones; Joan Locatelli;

Associated Retinal Consultants, PC, Royal Oak, Mich: Principal Investigator: Alan Ruby, MD; Co-Investigators: Antonio Capone, Jr, MD; Bruce Garretson, MD; Tarek Hassan, MD; Michael T. Trese, MD; George A. Williams, MD; Clinic Coordinators: Virginia Regan, RN; Patricia Manatrey, RN; Photographers: Patricia Streasick; Lynette Szydlowski; Fran McIver; Craig Bridges; Technicians: Cheryl Stanley; Kristi Cumming, RN; Bobbie Lewis, RN; Mary Zajechowski; Past Participating Personnel: Principal Investigator: Raymond R. Margherio, MD†; Co-Investigators: Morton S. Cox, MD; Jane Camille Werner, MD; Photographers: Rachel Falk; Patricia Siedlak; Technician: Cheryl Neubert, RN;

Devers Eye Institute, Portland, Ore: Principal Investigator: Michael L. Klein, MD; Co-Investigators: J. Timothy Stout, MD, PhD; Adrian O'Malley, MD; Andreas K. Lauer, MD; Joseph E. Robertson, MD; David J. Wilson, MD; Clinic Coordinator: Carolyn Beardsley; Photographers: Hiroko Anderson; Patrick Wallace; Technicians: Garland Smith; Shannon Howard; Past Participating Personnel: Principal Investigator: Richard F. Dreyer, MD; Co-Investigators: Colin Ma, MD; Richard G. Chenoweth, MD; John D. Zilis, MD; Photographers: Milton Johnson; Patrick Rice; Howard Daniel; Technicians: Harold Crider; Sheryl Parker; Kathryn Sherman;

Emory University, Atlanta, Ga: Principal Investigator: Daniel F. Martin, MD; Co-Investigators: Thomas M. Aaberg Sr, MD; Paul Sternberg Jr, MD; Clinic Coordinators: Linda T. Curtis; Bora Ju; Photographers: James Gilman; Bob Myles; Sandra Strittman; Research Associates: Christina Gentry; Hannah Yi; Past Participating Personnel: Principal Investigators: Antonio Capone Jr, MD; Michael Lambert, MD; Travis Meredith, MD; Co-Investigators: Thomas M. Aaberg Jr, MD; David Saperstein, MD; Jennifer I. Lim, MD; Clinic Coordinator: Barbara Stribling; Photographers: Denise Armiger; Ray Swords;

Ingalls Memorial Hospital, Harvey, Ill: Principal Investigator: David H. Orth, MD; Co-Investigators: Timothy P. Flood, MD; Joseph Civantos, MD; Serge deBustros, MD; Kirk H. Packo, MD; Pauline T. Merrill, MD; Jack A. Cohen, MD; Clinic Coordinators: Celeste Figliulo; Chris Morrison; Photographers: Douglas A. Bryant; Don Doherty; Marian McVicker; Technician:Tana Drefcinski;

Massachusetts Eye and Ear Infirmary, Boston, Mass: Principal Investigator: Johanna M. Seddon, MD, ScM; Co-Investigator: Michael K. Pinnolis, MD; Clinic Coordinators: Nancy Davis; Ilene Burton, RN; Tatiana Taitsel; Photographers: David Walsh; Jennifer Dubois-Moran; Charlene Callahan; Technician: Claudia Evans, OD; Past Participating Personnel: Clinic Coordinators: Kristin K. Snow, MS; Desiree A. Jones-Devonish; Valerie D. Crouse, MS; N. Jennifer Rosenberg, RN, MPH;

National Eye Institute Clinical Center, Bethesda: Principal Investigator: Emily Y. Chew, MD; Co-Investigators: Karl Csaky, MD, PhD; Frederick L. Ferris III, MD; Clinic Coordinators: Katherine Hall Shimel, RN; Merria A. Woods; Photographers: Ernest M. Kuehl; Patrick F. Ciatto; Marilois Palmer; Technicians: Gloria Babilonia-Ayukawa, RN, MHCA; Guy E. Foster; Linda Goodman; Young Ja Kim, RN; Iris J. Kivitz; Dessie Koutsandreas; Antoinette LaReau; Richard F. Mercer; Roula Nashwinter; Past Participating Personnel: Clinic Coordinator: Sally A. McCarthy, RN, MSN; Technicians: Leanne M. Ayres; Patrick Lopez; Anne Randalls;

University of Pittsburgh, Pittsburgh, Pa: Principal Investigator: Thomas R. Friberg, MD, MS; Co-Investigators: Andrew W. Eller, MD; Michael B. Gorin, MD, PhD; Clinic Coordinators: Shannon Nixon; Barbara Mack; Photographers: Diane Y. Curtin; Phyllis P. Ostroska; Edward Fijewski; Past Participating Personnel: Clinic Coordinator: Jane Alexander; Technicians: Melissa K. Paine; Patricia S. Corbin; Photographer: Joseph Warnicki;

The Johns Hopkins Medical Institutions, Baltimore: Principal Investigator: Susan B. Bressler, MD; Co-Investigators: Neil M. Bressler, MD; Gary Cassel, MD; Daniel Finkelstein, MD; Morton Goldberg, MD; Julia A. Haller, MD; Lois Ratner, MD; Andrew P. Schachat, MD; Steven H. Sherman, MD; Janet S. Sunness, MD; Clinic Coordinators: Sherrie Schenning; Catherine Sackett, RN; Photographers: Dennis Cain; David Emmert; Mark Herring; Jacquelyn McDonald; Rachel Falk; Technician: Stacy Wheeler; Past Participating Personnel: Clinic Coordinator: Mary Mcmillan; Photographer: Terry George;

Elman Retina Group, PA, Baltimore: Principal Investigator: Michael J. Elman, MD; Co-Investigators: Rex Ballinger, OD; Arturo Betancourt, MD; David Glasser, MD; Michael Herr, MD; Dahlia Hirsh, MD; Daniel Kilingsworth, MD; Paul Kohlhepp, MD; Joyce Lammlein, MD; Robert Z. Raden, MD; Ronald Seff, MD;Martin Shuman, MD; Clinic Coordinators: JoAnn Starr; Anita Carrigan; Photographers: Peter Sotirakos; Theresa Cain; Technician: Terri Mathews; Past Participating Personnel: Clinic Coordinator: Christine Ringrose;

University of Wisconsin–Madison: Principal Investigators: Suresh R. Chandra, MD; Justin L. Gottlieb, MD; Co-Investigators: Michael S. Ip, MD; Ronald Klein, MD, MPH; T. Michael Nork, MD, MS; Thomas S. Stevens, MD; Barbara A. Blodi, MD; Michael Altaweel, MD; Barbara E. K. Klein, MD; Clinic Coordinators: Michelle Olson; Barbara Soderling; Margo Blatz; Jennie R. Perry-Raymond; Kathryn Burke; Photographers: Gene Knutson; John Peterson; Denise Krolnik; Technicians: Robert Harrison; Guy Somers, RN; Past Participating Personnel: Principal Investigator: Frank L. Myers, MD; Co-Investigators: Ingolf Wallow, MD; Timothy W. Olsen, MD; George Bresnik, MD; G. De Venecia, MD; Clinic Coordinators: Tracy Perkins, MPH; Wendy Walker; Jennifer L. Miller; Photographers: Michael Neider; Hugh D. Wabers; Greg Weber; Technician: Helen E. Lyngaas Myers;

University of Wisconsin Reading Center, Madison: Principal Investigators: Matthew D. Davis, MD; Barbara E. K. Klein, MD; Ronald Klein, MD, MPH; Co-Investigator: Larry Hubbard, MA; Photography Protocol Monitors: Michael Neider; Hugh D. Wabers; Senior Photography Graders: Yvonne L. Magli; Sarah Ansay; Jane Armstrong; Photography Graders: Kristine Lang; Darlene Badal; Patricia L. Geithman; Kathleen D.Miner; Kristi L. Dohm; Barbara Esser; Cynthia Hurtenbach; Shirley Craanen; Mary Webster; Julee Elledge; Susan Reed; Wendy Benz; James Reimers; Statisticians: Marian R. Fisher, PhD; Ronald Gangnon, PhD; William King, MS; Chunyang Gai, PhD; Computer staff: James Baliker; Alistair Carr; Kurt Osterby; Data Manager: Linda Kastorff; Research Program Manager: Nancy Robinson; Administration Program Specialist: James Onofrey; Coordination staff: Kathleen E. Glander; Judith Brickbauer;

Centers for Disease Control and Prevention, Central Laboratory, Atlanta: Dayton Miller, PhD; Anne Sowell, PhD; Elaine Gunter, MT; Past Participating Personnel: Barbara Bowman, PhD;

Coordinating Center–The EMMES Corporation, Rockville: Principal Investigators: Anne S. Lindblad, PhD; Roy C. Milton, PhD; Co-Investigators: Traci E. Clemons, PhD; Fred Ederer, MA, FACE; Gary Gensler, MS; Genetics Monitor: Alice Henning, MS; Protocol Monitors: Gary Entler; Wendy McBee, MA; Kiana Roberts; Elaine Stine; Computer Analyst: Stuart H. Berlin; Administration: Kate Tomlin; Past Participating Personnel: Administration: Sophia Pallas; Phyllis R. Scholl; Susan A. Mengers; Co-Investigator: Ravinder Anand, PhD;

National Eye Institute Project Office, Bethesda: Study Chairman and Principal Investigator: Frederick L. Ferris III, MD; Co-Investigators: Robert D. Sperduto, MD; Natalie Kurinij, PhD; Emily Y. Chew, MD.
