# Supplementary material for: Relationships Between Diet and Geographic Atrophy Progression in the Age-Related Eye Diseases Studies 1 and 2
Source: Nutrients. 2025 Feb 22;17(5):771. doi: 10.3390/nu17050771 (PMC11901604; doi:10.3390/nu17050771)
Supplement: Supplementary file 1 [file nutrients-17-00771-s001.zip › Supplementary material.pdf]

Supplement

Supplementary Text

Statistical Methods for Analyses of Change in the Geographic Atrophy Variables over Time

Mixed-model repeated-measures regression was performed with the geographic atrophy (GA) variable as the outcome measure. The models included the exposure variable of interest (i.e., aMedi, component, or nutrient quantiles), years from first time-point with GA (to account for the repeated measures), and their interaction term. The models also included terms for age, sex, smoking status, total calorie intake, and GA variable (i.e., (i) square root of GA area or (ii) GA proximity) at first time-point with GA, to account for differences among participants. Total calorie intake was included to decrease confounding and reduce extraneous variation from factors like physical activity and metabolic efficiency [1,2]. In analyses of each of the nine aMedi components, the models also included a term for the component-specific aMedi (i.e., modified to exclude that component). In all area analyses that included both central and non-central GA, the models included a term for presence/absence of central involvement at first time-point with GA. In the Age-Related Eye Diseases Study (AREDS) area analyses, the models also included a term for presence/absence of GA in fellow eye at first time-point with GA; this term was not included in the AREDS2 analyses, as the information was not available for all eyes. In the AREDS2 area analyses, the models also included a term for GA configuration at first time-point with GA; this term was not included in the AREDS analyses, as the information was not available for all eyes. To account for the correlation between both eyes of the same participant and between different visits of the same eye, an unstructured and a first-order autoregressive covariance structure (UN@AR(1)), respectively, was specified [3].

For the proximity analyses, on the rare occasions that proximity reached zero during follow-up, all subsequent time-points were censored. For both outcome measures, in the primary analyses, all longitudinal data were used. In separate supplementary analyses of the AREDS, data were used only for the duration of the 5-year clinical trial.

Statistical Methods for Analyses of Change in the Best-Corrected Visual Acuity over Time

Specifically, the models included the exposure variable of interest (i.e., aMedi, component, or nutrient quantiles), years from first time-point, and their interaction term. The models also included terms for age, sex, smoking status, total calorie intake, best-corrected visual acuity (BCVA) at first time-point with GA, and GA proximity at first time-point with GA. In analyses of each of the nine aMedi components, the models also included a term for the component-specific aMedi. In analyses that included both central and non-central GA, the models also included a term for presence/absence of central involvement at first time-point with GA. In the AREDS2 analyses, the models also included a term for GA configuration at first time-point with GA. An unstructured and first-order autoregressive covariance structure (UN@AR(1)) was specified.

References

1. Rhee JJ, Cho E, Willett WC. Energy adjustment of nutrient intakes is preferable to adjustment using body weight and physical activity in epidemiological analyses. *Public Health Nutr*. May 2014;17(5):1054-60.
2. Willett WC, Howe GR, Kushi LH. Adjustment for total energy intake in epidemiologic studies. *Am J Clin Nutr*. Apr 1997;65(4 Suppl):1220S-1228S; discussion 1229S-1231S.
3. Galecki AT. General class of covariance structures for two or more repeated factors in longitudinal data analysis. *Communications in Statistics - Theory and Methods*. 1994/01/01 1994;23(11):3105-3119.

Table S1. Estimates of Daily Nutrient Intake from Foods in the Study Populations.

	AREDS				AREDS2			
	GA Area Study Population (n = 657 eyes of 508 participants)		GA Proximity Study Population (n = 390 eyes of 328 participants)		GA Area Study Population (n = 1179 eyes of 867 participants)		GA Proximity Study Population (n = 826 eyes of 652 participants)	
	Mean (SD)	Median	Mean (SD)	Median	Mean (SD)	Median	Mean (SD)	Median
Calories, kcal	1528.9 (649.5)	1440.6	1508.9 (672.6)	1382.6	1699.0 (605.4)	1652.6	1699.2 (603.6)	1653.1
Vitamin A, IU	6773.8 (3859.3)	5910.0	6711.2 (3992.5)	5758.5	8713.6 (5245.2)	7419.0	8569.8 (5093.6)	7393.2
Vitamin A, RAE	.	.	.	.	820.7 (427.0)	735.4	818.2 (435.9)	727.3
Retinol, mcg	597.6 (363.9)	529.0	593.0 (377.2)	523.3	448.9 (323.6)	387.9	455.6 (343.4)	387.3
Vitamin D, mcg	5.8 (3.6)	5.1	5.8 (3.9)	5.0	4.7 (3.4)	3.9	4.6 (3.1)	4.0
Vitamin E, mg	10.5 (6.5)	9.3	10.2 (6.6)	8.6	7.2 (3.6)	6.6	7.2 (3.6)	6.5
Vitamin C, mg	100.7 (56.4)	96.2	100.5 (56.5)	96.8	108.3 (63.4)	101.2	106.1 (60.8)	99.4
Thiamine, mg	1.3 (0.6)	1.3	1.3 (0.6)	1.2	1.4 (0.6)	1.4	1.4 (0.5)	1.4
Riboflavin, mg	1.6 (0.8)	1.6	1.6 (0.9)	1.5	2.0 (0.8)	1.9	2.0 (0.8)	1.9
Niacin, mg	16.9 (7.7)	15.9	16.5 (8.0)	15.3	21.2 (8.0)	20.4	21.1 (7.9)	20.3
Vitamin B6, mg	1.6 (0.8)	1.5	1.6 (0.8)	1.4	1.9 (0.8)	1.8	1.9 (0.8)	1.8
Folate, mcg	345.1 (165.6)	314.7	339.2 (172.8)	305.8	400.6 (172.0)	377.8	392.5 (159.8)	373.8
Natural food folate, mcg	.	.	.	.	267.7 (114.3)	255.0	263.7 (107.4)	253.0
Folic acid, mcg	.	.	.	.	373.7 (278.4)	428.0	376.4 (275.9)	436.0
Vitamin B12, mcg	4.6 (2.9)	4.0	4.6 (3.2)	4.0	6.3 (3.8)	5.6	6.3 (4.0)	5.5
Beta-carotene, mcg	2533.0 (1824.1)	2057.8	2508.9 (1892.0)	2022.2	3923.1 (2750.8)	3223.1	3831.0 (2651.1)	3186.3
Beta-carotene equivalents, mcg	2868.1 (2048.3)	2329.9	2839.7 (2108.6)	2283.8	.	.	.	.
Alpha-carotene, mcg	534.9 (508.8)	378.1	525.5 (498.8)	373.4	686.9 (630.3)	480.6	673.0 (631.1)	477.7
Beta-cryptoxanthin, mcg	135.2 (86.8)	126.1	136.0 (87.0)	126.9	136.4 (108.9)	110.0	135.4 (110.5)	107.7
Lutein and zeaxanthin, mcg	1573.8 (1120.6)	1283.3	1542.8 (1101.0)	1209.3	3293.1 (2761.1)	2557.8	3229.3 (2703.7)	2561.9
Lycopene, mcg	4438.4 (3134.7)	3748.1	4363.9 (3028.7)	3559.4	5565.1 (4421.7)	4438.1	5449.1 (4141.1)	4414.7
Calcium, mg	765.7 (445.8)	688.4	763.8 (481.3)	673.7	703.4 (331.2)	646.2	706.6 (329.0)	652.9
Magnesium, mg	244.4 (98.0)	234.2	241.8 (100.7)	232.8	298.3 (111.0)	293.0	297.3 (108.3)	292.1
Iron, mg	11.5 (6.2)	10.1	11.2 (6.4)	9.8	13.2 (5.6)	12.4	13.1 (5.6)	12.4
Zinc, mg	10.2 (5.9)	8.8	10.2 (6.5)	8.7	10.7 (4.5)	9.9	10.8 (4.6)	10.1
Copper, mg	0.9 (0.4)	0.9	0.9 (0.4)	0.8	1.4 (0.7)	1.3	1.4 (0.7)	1.3
Selenium, mcg	81.1 (35.5)	77.2	80.2 (37.4)	73.3	14.6 (31.6)	0.0	14.6 (30.7)	0.0
Saturated fat, % kcal	11.7 (3.6)	11.4	11.8 (3.8)	11.4	10.9 (2.6)	10.8	235.5 (103.9)	225.1
Monounsaturated fat, % kcal	13.3 (3.5)	13.5	13.2 (3.6)	13.4	13.4 (3.2)	13.1	11.0 (2.6)	10.8
Cholesterol, mg	174.9 (108.8)	150.4	171.2 (106.8)	149.5	234.4 (107.0)	222.5	13.4 (3.2)	13.1
Oleic acid, mg per 1000 kcal	13956.9 (3808.3)	14211.3	13805.4 (3882.3)	13927.8	13711.0 (3400.2)	13349.5	13761.4 (3434.4)	13332.5
Linoleic acid, mg per 1000 kcal	8656.0 (2679.6)	8577.4	8515.4 (2615.0)	8438.7	6449.0 (2019.2)	6193.5	6480.2 (2096.1)	6196.9
a-Linolenic acid, mg per 1000 kcal	825.2 (287.0)	786.9	824.1 (282.3)	802.8	708.2 (420.3)	650.7	716.2 (470.7)	651.1
EPA, mg per 1000 kcal	20.9 (20.6)	15.8	21.8 (21.4)	16.4	72.3 (129.8)	36.6	72.6 (141.2)	35.1
DPA, mg per 1000 kcal	.	.	.	.	15.1 (15.1)	11.7	15.0 (15.8)	11.7
DHA, mg per 1000 kcal	32.9 (24.2)	27.7	34.0 (25.2)	28.4	100.3 (106.0)	72.7	98.7 (109.0)	72.5
EPA+DHA, mg per 1000 kcal	53.9 (44.3)	43.8	55.8 (46.1)	45.2	172.9 (231.8)	111.0	171.5 (246.5)	107.2
EPA+DPA+DHA, mg per 1000 kcal	.	.	.	.	132.6 (108.0)	107.6	127.7 (100.8)	106.3
Arachidonic acid, mg per 1000 kcal	48.7 (23.7)	46.0	48.5 (23.7)	46.6	75.1 (33.7)	70.6	75.2 (33.6)	71.2
Galactose, gm	0.2 (0.4)	0.1	0.2 (0.4)	0.1	.	.	.	.
Lactose, gm	18.3 (14.7)	15.5	18.5 (16.0)	15.3	13.3 (10.6)	10.5	13.6 (10.9)	10.9
Alcohol, gm	7.0 (14.1)	0.9	6.7 (14.2)	0.6	7.8 (13.6)	1.5	7.9 (14.0)	1.5
Soluble Dietary Fiber, gm	5.4 (2.4)	5.1	5.3 (2.4)	4.9	.	.	.	.
Insoluble Dietary Fiber, gm	9.4 (4.5)	8.8	9.3 (4.4)	8.8	.	.	.	.
Fiber, gm	.	.	.	.	18.8 (8.5)	17.8	18.6 (8.0)	17.6
Total choline, gm	.	.	.	.	306.8 (111.4)	302.3	307.3 (108.9)	303.1
Free choline, gm	.	.	.	.	68.2 (24.9)	66.1	67.9 (24.5)	66.2
Glycemic index	.	.	.	.	51.9 (3.8)	52.2	51.9 (3.7)	52.1
Glycemic load	.	.	.	.	105.2 (44.0)	101.2	104.7 (44.1)	99.5

Abbreviations: AREDS=Age-Related Eye Diseases Study; DHA=docosahexaenoic acid; DPA=docosapentaenoic acid; EPA=eicosapentaenoic acid; GA=geographic atrophy; IU=international units; RAE=retinol activity equivalents; SD=standard deviation

Table S2. Geographic Atrophy Area-Based Progression Rates, according to Quantiles of the Alternative Mediterranean Dietary Index and its Components, in the Age-Related Eye Diseases Studies 1 and 2.

	AREDS (n = 657 eyes of 508 participants)			AREDS2 (n = 1179 eyes of 867 participants)		
	Estimate, mm/year (CI)	Pairwise P Value	Interaction P Value	Estimate, mm/year (CI)	Pairwise P Value	Interaction P Value
aMedi						
Tertile 1	0.260 (0.245,0.275)	.	0.2488	0.297 (0.277,0.316)	.	0.0345
Tertile 2	0.259 (0.244,0.275)	0.9562	.	0.280 (0.257,0.302)	0.2694	.
Tertile 3	0.242 (0.223,0.260)	0.1258	.	0.259 (0.238,0.279)	0.0095	.
Whole fruit						
Quartile 1	0.281 (0.263,0.298)	.	0.0004	0.308 (0.284,0.333)	.	0.0071
Quartile 2	0.254 (0.235,0.274)	0.0505	.	0.279 (0.255,0.303)	0.0953	.
Quartile 3	0.227 (0.210,0.245)	<.0001	.	0.281 (0.258,0.305)	0.1164	.
Quartile 4	0.260 (0.240,0.279)	0.1197	.	0.247 (0.223,0.272)	0.0005	.
Vegetables						
Quartile 1	0.278 (0.261,0.295)	.	0.0043	0.268 (0.244,0.292)	.	0.3387
Quartile 2	0.255 (0.236,0.273)	0.0631	.	0.283 (0.260,0.306)	0.3566	.
Quartile 3	0.249 (0.231,0.268)	0.0245	.	0.296 (0.271,0.321)	0.1086	.
Quartile 4	0.231 (0.211,0.251)	0.0004	.	0.269 (0.245,0.294)	0.9254	.
Whole grains						
Quartile 1	0.274 (0.257,0.291)	.	0.0232	0.281 (0.255,0.307)	.	0.8471
Quartile 2	0.255 (0.237,0.273)	0.1371	.	0.274 (0.251,0.297)	0.6929	.
Quartile 3	0.234 (0.215,0.253)	0.0021	.	0.287 (0.263,0.310)	0.7482	.
Quartile 4	0.256 (0.236,0.276)	0.1800	.	0.274 (0.249,0.298)	0.6757	.
Nuts						
Quartile 1	0.263 (0.246,0.281)	.	0.0856	0.287 (0.263,0.310)	.	0.1880
Quartile 2	0.257 (0.235,0.278)	0.6357	.	0.269 (0.244,0.294)	0.3098	.
Quartile 3	0.236 (0.218,0.254)	0.0307	.	0.296 (0.272,0.319)	0.5908	.
Quartile 4	0.265 (0.247,0.283)	0.8919	.	0.262 (0.238,0.287)	0.1546	.
Legumes						
Quartile 1	0.269 (0.250,0.287)	.	0.0281	0.279 (0.251,0.307)	.	0.7747
Quartile 2	0.267 (0.250,0.284)	0.8969	.	0.273 (0.248,0.298)	0.7634	.
Quartile 3	0.234 (0.216,0.253)	0.0102	.	0.287 (0.267,0.307)	0.6432	.
Quartile 4	0.250 (0.230,0.269)	0.1699	.	0.272 (0.246,0.298)	0.7209	.
Red meat*						
Quartile 1	0.233 (0.216,0.250)	.	0.0005	0.314 (0.291,0.337)	.	0.0002
Quartile 2	0.284 (0.266,0.302)	<.0001	.	0.292 (0.268,0.315)	0.1868	.
Quartile 3	0.247 (0.228,0.265)	0.2867	.	0.257 (0.231,0.282)	0.0011	.
Quartile 4	0.263 (0.243,0.284)	0.0269	.	0.248 (0.224,0.272)	<.0001	.
Fish						
Quartile 1	0.262 (0.244,0.281)	.	0.1131	0.279 (0.249,0.308)	.	0.3844
Quartile 2	0.261 (0.244,0.277)	0.9225	.	0.270 (0.250,0.289)	0.6216	.
Quartile 3	0.261 (0.242,0.280)	0.9420	.	0.296 (0.273,0.319)	0.3630	.
Quartile 4	0.233 (0.213,0.253)	0.0376	.	0.275 (0.246,0.303)	0.8611	.
MUFA: SFA						
Quartile 1	0.230 (0.211,0.249)	.	0.0004	0.302 (0.277,0.326)	.	0.0291
Quartile 2	0.246 (0.227,0.265)	0.2455	.	0.286 (0.264,0.308)	0.3468	.
Quartile 3	0.286 (0.268,0.305)	<.0001	.	0.275 (0.249,0.300)	0.1371	.
Quartile 4	0.257 (0.239,0.274)	0.0459	.	0.249 (0.224,0.275)	0.0037	.
Alcohol						
In interval	0.253 (0.244,0.263)	.	0.1898	0.239 (0.205,0.273)	.	.

Not in interval	0.274 (0.244,0.304)	0.1898	.	0.285 (0.272,0.298)	0.0130	0.0130

Abbreviations: aMedi=Alternative Mediterranean Diet Index; AREDS=Age-Related Eye Diseases Study; CI=confidence interval; MUFA: SFA=monounsaturated fatty acid: saturated fatty acid

* For red meat, higher quartiles refer to lower levels of intake, which is more adherent to the Alternative Mediterranean Diet Index

Table S3. Geographic Atrophy Area-Based Progression Rates, according to Quantiles of Nutrient Intake, in the Age-Related Eye Diseases Studies 1 and 2.

	AREDS (n = 657 eyes of 508 participants)			AREDS2 (n = 1179 eyes of 867 participants)		
Nutrient	Estimate, mm/year (CI)	Pairwise P Value	Interaction P Value	Estimate, mm/year (CI)	Pairwise P Value	Interaction P Value
Vitamin A, IU						
Tertile 1	0.253 (0.239,0.268)	.	<.0001	0.293 (0.272,0.314)	.	0.1736
Tertile 2	0.284 (0.267,0.301)	0.0064	.	0.278 (0.258,0.298)	0.3204	.
Tertile 3	0.229 (0.212,0.246)	0.0296	.	0.264 (0.242,0.286)	0.0616	.
Vitamin A, RAE						
Tertile 1	.	.	.	0.292 (0.272,0.313)	.	0.2772
Tertile 2	.	.	.	0.274 (0.253,0.295)	0.2188	.
Tertile 3	.	.	.	0.270 (0.249,0.291)	0.1357	.
Retinol, mcg						
Tertile 1	0.235 (0.220,0.251)	.	0.0051	0.281 (0.259,0.303)	.	0.7429
Tertile 2	0.271 (0.256,0.287)	0.0014	.	0.283 (0.263,0.304)	0.8632	.
Tertile 3	0.260 (0.243,0.276)	0.0364	.	0.272 (0.251,0.293)	0.5840	.
Vitamin D, mcg						
Tertile 1	0.244 (0.229,0.259)	.	0.0438	0.285 (0.264,0.306)	.	0.1225
Tertile 2	0.252 (0.236,0.268)	0.4880	.	0.290 (0.269,0.310)	0.7552	.
Tertile 3	0.272 (0.256,0.288)	0.0144	.	0.260 (0.238,0.282)	0.1071	.
Vitamin E, mg						
Tertile 1	0.252 (0.236,0.269)	.	0.1113	0.308 (0.288,0.329)	.	<.0001
Tertile 2	0.268 (0.253,0.284)	0.1703	.	0.286 (0.266,0.307)	0.1368	.
Tertile 3	0.245 (0.230,0.261)	0.5273	.	0.240 (0.219,0.261)	<.0001	.
Vitamin C, mg						
Tertile 1	0.269 (0.254,0.284)	.	0.0833	0.303 (0.282,0.324)	.	0.0088
Tertile 2	0.249 (0.234,0.265)	0.0792	.	0.277 (0.257,0.297)	0.0788	.
Tertile 3	0.245 (0.227,0.262)	0.0435	.	0.256 (0.234,0.278)	0.0022	.
Thiamine, mg						
Tertile 1	0.257 (0.242,0.272)	.	0.0749	0.283 (0.262,0.304)	.	0.3526
Tertile 2	0.269 (0.251,0.287)	0.2996	.	0.287 (0.266,0.309)	0.7784	.
Tertile 3	0.242 (0.226,0.258)	0.1641	.	0.267 (0.246,0.288)	0.2742	.
Riboflavin, mg						
Tertile 1	0.244 (0.229,0.259)	.	0.0015	0.285 (0.264,0.306)	.	0.7471
Tertile 2	0.280 (0.264,0.296)	0.0017	.	0.274 (0.253,0.295)	0.4534	.
Tertile 3	0.242 (0.226,0.259)	0.8688	.	0.278 (0.256,0.299)	0.6211	.
Niacin, mg						
Tertile 1	0.251 (0.236,0.266)	.	0.0334	0.289 (0.269,0.309)	.	0.4321
Tertile 2	0.273 (0.256,0.289)	0.0607	.	0.276 (0.255,0.297)	0.3714	.
Tertile 3	0.243 (0.226,0.259)	0.4420	.	0.270 (0.248,0.292)	0.2113	.
Vitamin B6, mg						
Tertile 1	0.254 (0.239,0.269)	.	0.0705	0.296 (0.275,0.317)	.	0.0001
Tertile 2	0.270 (0.253,0.287)	0.1472	.	0.297 (0.277,0.317)	0.9227	.
Tertile 3	0.243 (0.226,0.259)	0.3352	.	0.240 (0.219,0.262)	0.0003	.
Folate, mcg						
Tertile 1	0.257 (0.242,0.271)	.	0.8021	0.308 (0.288,0.329)	.	<.0001
Tertile 2	0.251 (0.234,0.267)	0.6120	.	0.288 (0.268,0.308)	0.1588	.
Tertile 3	0.259 (0.241,0.276)	0.8570	.	0.237 (0.215,0.258)	<.0001	.
Natural Food Folate						
Tertile 1	.	.	.	0.302 (0.281,0.323)	.	0.0108
Tertile 2	.	.	.	0.278 (0.257,0.298)	0.1013	.
Tertile 3	.	.	.	0.257 (0.235,0.278)	0.0026	.

Folic Acid						
Tertile 1	.	.	.	0.302 (0.282,0.323)	.	0.0059
Tertile 2	.	.	.	0.279 (0.257,0.300)	0.1152	.
Tertile 3	.	.	.	0.255 (0.235,0.276)	0.0013	.
Vitamin B12, mcg						
Tertile 1	0.245 (0.228,0.261)	.	0.2364	0.294 (0.274,0.315)	.	0.0664
Tertile 2	0.264 (0.249,0.279)	0.0899	.	0.283 (0.262,0.303)	0.4291	.
Tertile 3	0.256 (0.239,0.273)	0.3411	.	0.260 (0.239,0.281)	0.0218	.
Beta-carotene, mcg						
Tertile 1	0.260 (0.245,0.274)	.	0.0043	0.291 (0.271,0.311)	.	0.0160
Tertile 2	0.270 (0.254,0.287)	0.3398	.	0.291 (0.270,0.312)	0.9984	.
Tertile 3	0.232 (0.214,0.249)	0.0148	.	0.253 (0.231,0.274)	0.0118	.
Beta-carotene equivalents, mcg						
Tertile 1	0.257 (0.242,0.271)	.	<.0001	.	.	.
Tertile 2	0.284 (0.267,0.300)	0.0164	.	.	.	.
Tertile 3	0.224 (0.207,0.240)	0.0035	.	.	.	.
Alpha-carotene, mcg						
Tertile 1	0.249 (0.233,0.265)	.	0.0005	0.288 (0.267,0.309)	.	0.1163
Tertile 2	0.280 (0.264,0.295)	0.0072	.	0.287 (0.267,0.306)	0.9362	.
Tertile 3	0.236 (0.220,0.252)	0.2836	.	0.259 (0.237,0.281)	0.0678	.
Beta-cryptoxanthin, mcg						
Tertile 1	0.253 (0.238,0.267)	.	0.8614	0.298 (0.276,0.319)	.	0.0913
Tertile 2	0.256 (0.240,0.272)	0.7661	.	0.278 (0.258,0.298)	0.1882	.
Tertile 3	0.259 (0.241,0.276)	0.5883	.	0.264 (0.243,0.285)	0.0293	.
Lutein and zeaxanthin, mcg						
Tertile 1	0.254 (0.240,0.269)	.	0.0161	0.277 (0.257,0.297)	.	0.7496
Tertile 2	0.269 (0.254,0.285)	0.1634	.	0.286 (0.264,0.307)	0.5676	.
Tertile 3	0.233 (0.214,0.252)	0.0836	.	0.275 (0.254,0.296)	0.8728	.
Lycopene, mcg						
Tertile 1	0.275 (0.260,0.290)	.	0.0056	0.269 (0.248,0.291)	.	0.2632
Tertile 2	0.245 (0.230,0.261)	0.0076	.	0.292 (0.272,0.312)	0.1322	.
Tertile 3	0.242 (0.225,0.259)	0.0046	.	0.273 (0.252,0.294)	0.7961	.
Calcium, mg						
Tertile 1	0.256 (0.241,0.272)	.	0.8405	0.303 (0.282,0.324)	.	0.0008
Tertile 2	0.258 (0.242,0.274)	0.8946	.	0.284 (0.264,0.304)	0.1928	.
Tertile 3	0.251 (0.235,0.268)	0.6610	.	0.246 (0.224,0.268)	0.0002	.
Magnesium, mg						
Tertile 1	0.252 (0.237,0.266)	.	0.7593	0.278 (0.257,0.300)	.	0.4458
Tertile 2	0.256 (0.239,0.272)	0.7185	.	0.288 (0.268,0.308)	0.5126	.
Tertile 3	0.260 (0.243,0.278)	0.4591	.	0.269 (0.248,0.290)	0.5533	.
Iron, mg						
Tertile 1	0.257 (0.242,0.272)	.	0.8920	0.304 (0.283,0.325)	.	0.0127
Tertile 2	0.257 (0.240,0.273)	0.9921	.	0.273 (0.252,0.293)	0.0362	.
Tertile 3	0.252 (0.235,0.269)	0.6640	.	0.261 (0.240,0.282)	0.0043	.
Zinc, mg						
Tertile 1	0.254 (0.239,0.269)	.	0.5609	0.283 (0.262,0.303)	.	0.0960
Tertile 2	0.263 (0.246,0.280)	0.4609	.	0.293 (0.272,0.314)	0.4960	.
Tertile 3	0.250 (0.234,0.266)	0.7201	.	0.261 (0.240,0.282)	0.1421	.
Copper, mg						
Tertile 1	0.255 (0.240,0.270)	.	0.4227	0.296 (0.274,0.318)	.	0.0783
Tertile 2	0.263 (0.247,0.279)	0.4819	.	0.281 (0.261,0.301)	0.3232	.
Tertile 3	0.247 (0.229,0.264)	0.4937	.	0.261 (0.240,0.282)	0.0248	.
Selenium, mcg						
Tertile 1	0.259 (0.243,0.274)	.	0.0801	0.269 (0.253,0.285)	.	0.1482

Tertile 2	0.242 (0.227,0.258)	0.1407	.	0.289 (0.243,0.334)	0.4251	.
Tertile 3	0.268 (0.251,0.285)	0.4331	.	0.294 (0.274,0.314)	0.0564	.
Saturated fat, % kcal						
Tertile 1	0.259 (0.242,0.276)	.	0.5255	0.249 (0.228,0.271)	.	0.0032
Tertile 2	0.248 (0.232,0.264)	0.3286	.	0.283 (0.263,0.304)	0.0255	.
Tertile 3	0.259 (0.244,0.274)	0.9666	.	0.301 (0.280,0.321)	0.0008	.
Monounsaturated fat, % kcal						
Tertile 1	0.243 (0.226,0.260)	.	0.2195	0.275 (0.254,0.297)	.	0.8603
Tertile 2	0.258 (0.241,0.274)	0.2131	.	0.283 (0.263,0.304)	0.5955	.
Tertile 3	0.262 (0.248,0.277)	0.0891	.	0.278 (0.257,0.299)	0.8802	.
Cholesterol, mg						
Tertile 1	0.258 (0.242,0.274)	.	0.3701	0.268 (0.247,0.289)	.	0.0351
Tertile 2	0.245 (0.228,0.262)	0.2719	.	0.267 (0.246,0.288)	0.9686	.
Tertile 3	0.261 (0.246,0.276)	0.8276	.	0.301 (0.280,0.322)	0.0270	.
Oleic acid, mg per 1000 kcal						
Tertile 1	0.243 (0.226,0.261)	.	0.2345	0.280 (0.259,0.302)	.	0.5248
Tertile 2	0.256 (0.240,0.272)	0.3037	.	0.287 (0.266,0.307)	0.6743	.
Tertile 3	0.263 (0.248,0.278)	0.0890	.	0.270 (0.249,0.291)	0.4907	.
Linoleic acid, mg per 1000 kcal						
Tertile 1	0.260 (0.243,0.278)	.	0.6401	0.268 (0.246,0.289)	.	0.0087
Tertile 2	0.257 (0.241,0.273)	0.8013	.	0.305 (0.284,0.325)	0.0146	.
Tertile 3	0.250 (0.235,0.265)	0.3723	.	0.263 (0.243,0.284)	0.7689	.
a-Linolenic acid, mg per 1000 kcal						
Tertile 1	0.254 (0.237,0.271)	.	0.3787	0.280 (0.258,0.301)	.	0.0152
Tertile 2	0.265 (0.248,0.282)	0.3656	.	0.302 (0.281,0.324)	0.1481	.
Tertile 3	0.249 (0.234,0.264)	0.6656	.	0.259 (0.240,0.279)	0.1658	.
EPA, mg per 1000 kcal						
Tertile 1	0.264 (0.249,0.278)	.	0.1929	0.277 (0.257,0.298)	.	0.1899
Tertile 2	0.244 (0.228,0.260)	0.0722	.	0.293 (0.272,0.314)	0.2828	.
Tertile 3	0.257 (0.239,0.275)	0.5703	.	0.266 (0.244,0.287)	0.4428	.
DHA, mg per 1000 kcal						
Tertile 1	0.263 (0.248,0.278)	.	0.2708	0.273 (0.252,0.294)	.	0.0663
Tertile 2	0.256 (0.240,0.271)	0.4964	.	0.296 (0.277,0.316)	0.1105	.
Tertile 3	0.244 (0.226,0.262)	0.1063	.	0.263 (0.240,0.285)	0.4981	.
DPA, mg per 1000 kcal						
Tertile 1	.	.	.	0.277 (0.257,0.298)	.	0.4308
Tertile 2	.	.	.	0.289 (0.268,0.309)	0.4448	.
Tertile 3	.	.	.	0.269 (0.248,0.291)	0.5931	.
EPA+DHA, mg per 1000 kcal						
Tertile 1	0.263 (0.248,0.278)	.	0.4057	0.276 (0.255,0.297)	.	0.2010
Tertile 2	0.248 (0.233,0.264)	0.1888	.	0.293 (0.272,0.313)	0.2670	.
Tertile 3	0.253 (0.235,0.271)	0.4080	.	0.266 (0.244,0.288)	0.5065	.
EPA+DPA+DHA, mg per 1000 kcal						
Tertile 1	.	.	.	0.276 (0.255,0.296)	.	0.0410
Tertile 2	.	.	.	0.297 (0.277,0.316)	0.1392	.
Tertile 3	.	.	.	0.258 (0.235,0.281)	0.2695	.
Arachidonic acid, mg per 1000 kcal						
Tertile 1	0.261 (0.246,0.277)	.	0.5161	0.265 (0.245,0.286)	.	0.2415
Tertile 2	0.248 (0.231,0.264)	0.2513	.	0.283 (0.262,0.303)	0.2429	.
Tertile 3	0.256 (0.240,0.272)	0.6388	.	0.290 (0.268,0.312)	0.1033	.
Galactose, gm						
Tertile 1	0.262 (0.247,0.277)	.	0.2146	.	.	.
Tertile 2	0.258 (0.242,0.274)	0.7047	.	.	.	.
Tertile 3	0.242 (0.225,0.260)	0.0892	.	.	.	.

Lactose, gm						
Tertile 1	0.258 (0.242,0.273)	.	0.9196	0.269 (0.247,0.291)	.	0.5353
Tertile 2	0.253 (0.237,0.269)	0.6832	.	0.285 (0.265,0.306)	0.2793	.
Tertile 3	0.255 (0.239,0.271)	0.8238	.	0.281 (0.261,0.302)	0.4163	.
Alcohol, gm						
Tertile 1	0.272 (0.258,0.286)	.	0.0067	0.309 (0.288,0.330)	.	0.0025
Tertile 2	0.244 (0.226,0.261)	0.0126	.	0.259 (0.239,0.279)	0.0008	.
Tertile 3	0.241 (0.223,0.258)	0.0056	.	0.271 (0.250,0.293)	0.0143	.
Soluble Dietary Fiber, gm						
Tertile 1	0.270 (0.255,0.285)	.	0.0100	.	.	.
Tertile 2	0.255 (0.240,0.270)	0.1739	.	.	.	.
Tertile 3	0.233 (0.215,0.251)	0.0024	.	.	.	.
Insoluble Dietary Fiber, gm						
Tertile 1	0.260 (0.246,0.275)	.	0.0657	.	.	.
Tertile 2	0.241 (0.226,0.257)	0.0787	.	.	.	.
Tertile 3	0.268 (0.249,0.287)	0.5220	.	.	.	.
Fiber (Soluble or Insoluble), gm						
Tertile 1	.	.	.	0.298 (0.276,0.319)	.	0.0120
Tertile 2	.	.	.	0.285 (0.265,0.306)	0.4094	.
Tertile 3	.	.	.	0.254 (0.233,0.275)	0.0040	.
Total Choline						
Tertile 1	.	.	.	0.265 (0.245,0.286)	.	0.2253
Tertile 2	.	.	.	0.291 (0.270,0.312)	0.0883	.
Tertile 3	.	.	.	0.281 (0.260,0.303)	0.2868	.
Free Choline						
Tertile 1	.	.	.	0.300 (0.279,0.321)	.	0.0414
Tertile 2	.	.	.	0.272 (0.252,0.291)	0.0510	.
Tertile 3	.	.	.	0.264 (0.241,0.286)	0.0186	.
Glycemic Index						
Tertile 1	.	.	.	0.284 (0.264,0.304)	.	0.2192
Tertile 2	.	.	.	0.289 (0.267,0.310)	0.7623	.
Tertile 3	.	.	.	0.264 (0.242,0.285)	0.1736	.
Glycemic Load						
Tertile 1	.	.	.	0.283 (0.263,0.304)	.	0.5170
Tertile 2	.	.	.	0.285 (0.263,0.306)	0.9386	.
Tertile 3	.	.	.	0.269 (0.249,0.290)	0.3404	.

Abbreviations: AREDS=Age-Related Eye Diseases Study; CI=confidence interval; DHA=docosahexaenoic acid; DPA=docosapentaenoic acid; EPA=eicosapentaenoic acid; IU=international units; RAE=retinol activity equivalents

Table S4. Geographic Atrophy Area-Based Progression Rates, according to Quantiles of the Alternative Mediterranean Dietary Index and its Components, in the Geographic Atrophy Proximity Study Populations of the Age-Related Eye Diseases Studies 1 and 2.

	AREDS (n = 390 eyes of 328 participants)			AREDS2 (n = 826 eyes of 652 participants)		
	Estimate, mm/year (CI)	Pairwise P Value	Interaction P Value	Estimate, mm/year (CI)	Pairwise P Value	Interaction P Value
aMedi						
Tertile 1	0.296 (0.275,0.316)	.	0.0924	0.329 (0.304,0.354)	.	0.0713
Tertile 2	0.284 (0.262,0.307)	0.4714	.	0.320 (0.292,0.348)	0.6483	.
Tertile 3	0.259 (0.233,0.285)	0.0301	.	0.287 (0.260,0.314)	0.0271	.
Whole fruit						
Quartile 1	0.305 (0.280,0.330)	.	0.1400	0.367 (0.335,0.399)	.	0.0002
Quartile 2	0.285 (0.258,0.311)	0.2783	.	0.306 (0.276,0.335)	0.0053	.
Quartile 3	0.265 (0.238,0.291)	0.0289	.	0.314 (0.285,0.344)	0.0165	.
Quartile 4	0.273 (0.246,0.299)	0.0817	.	0.265 (0.233,0.298)	<.0001	.
Vegetables						
Quartile 1	0.312 (0.289,0.335)	.	0.0140	0.295 (0.263,0.326)	.	0.2854
Quartile 2	0.275 (0.248,0.302)	0.0416	.	0.314 (0.286,0.342)	0.3706	.
Quartile 3	0.272 (0.246,0.298)	0.0239	.	0.336 (0.305,0.367)	0.0652	.
Quartile 4	0.255 (0.225,0.285)	0.0028	.	0.304 (0.271,0.336)	0.6982	.
Whole grains						
Quartile 1	0.312 (0.287,0.337)	.	0.0479	0.312 (0.279,0.345)	.	0.9578
Quartile 2	0.278 (0.252,0.304)	0.0664	.	0.310 (0.281,0.339)	0.9259	.
Quartile 3	0.263 (0.237,0.289)	0.0082	.	0.319 (0.290,0.349)	0.7536	.
Quartile 4	0.274 (0.247,0.301)	0.0418	.	0.308 (0.277,0.339)	0.8568	.
Nuts						
Quartile 1	0.292 (0.268,0.316)	.	0.6005	0.317 (0.287,0.346)	.	0.6535
Quartile 2	0.292 (0.260,0.324)	0.9902	.	0.305 (0.274,0.337)	0.6128	.
Quartile 3	0.271 (0.246,0.296)	0.2350	.	0.326 (0.296,0.356)	0.6498	.
Quartile 4	0.278 (0.253,0.303)	0.4457	.	0.301 (0.269,0.332)	0.4691	.
Legumes						
Quartile 1	0.305 (0.279,0.332)	.	0.0027	0.328 (0.292,0.364)	.	0.3799
Quartile 2	0.304 (0.279,0.329)	0.9472	.	0.302 (0.270,0.334)	0.2995	.
Quartile 3	0.247 (0.223,0.272)	0.0015	.	0.323 (0.298,0.349)	0.8413	.
Quartile 4	0.274 (0.245,0.302)	0.1106	.	0.294 (0.262,0.326)	0.1662	.
Red meat*						
Quartile 1	0.252 (0.228,0.276)	.	0.0311	0.352 (0.323,0.380)	.	0.0003
Quartile 2	0.292 (0.267,0.317)	0.0247	.	0.331 (0.301,0.361)	0.3339	.
Quartile 3	0.296 (0.270,0.323)	0.0153	.	0.264 (0.231,0.296)	<.0001	.
Quartile 4	0.299 (0.270,0.328)	0.0158	.	0.291 (0.260,0.321)	0.0044	.
Fish						
Quartile 1	0.296 (0.270,0.322)	.	0.0030	0.306 (0.268,0.344)	.	0.6274
Quartile 2	0.309 (0.286,0.332)	0.4663	.	0.305 (0.280,0.329)	0.9506	.
Quartile 3	0.257 (0.230,0.283)	0.0354	.	0.329 (0.300,0.358)	0.3435	.
Quartile 4	0.252 (0.222,0.282)	0.0274	.	0.314 (0.276,0.351)	0.7798	.
MUFA: SFA						
Quartile 1	0.259 (0.233,0.284)	.	0.0008	0.336 (0.305,0.367)	.	0.0115
Quartile 2	0.259 (0.233,0.284)	0.9933	.	0.320 (0.292,0.348)	0.4517	.
Quartile 3	0.325 (0.299,0.351)	0.0004	.	0.325 (0.293,0.357)	0.6298	.
Quartile 4	0.290 (0.263,0.316)	0.0992	.	0.266 (0.234,0.298)	0.0022	.
Alcohol						
In interval	0.282 (0.268,0.295)	.	0.7463	0.246 (0.203,0.289)	.	.

Not in interval	0.289 (0.247,0.331)	0.7463	.	0.322 (0.306,0.339)	0.0011	0.0011

Abbreviations: aMedi=Alternative Mediterranean Diet Index; AREDS=Age-Related Eye Diseases Study; CI=confidence interval; MUFA: SFA=monounsaturated fatty acid: saturated fatty acid

* For red meat, higher quartiles refer to lower levels of intake, which is more adherent to the Alternative Mediterranean Diet Index

Table S5. Geographic Atrophy Area-Based Progression Rates, according to Quantiles of Nutrient Intake, in the Geographic Atrophy Proximity Study Populations of the Age-Related Eye Diseases Studies 1 and 2.

	AREDS (n = 390 eyes of 328 participants)			AREDS2 (n = 826 eyes of 652 participants)		
Nutrient	Estimate, mm/year (CI)	Pairwise P Value	Interaction P Value	Estimate, mm/year (CI)	Pairwise P Value	Interaction P Value
Vitamin A, IU						
Tertile 1	0.287 (0.266,0.307)	.	0.0061	0.328 (0.302,0.355)	.	0.2101
Tertile 2	0.308 (0.284,0.332)	0.1932	.	0.314 (0.288,0.339)	0.4315	.
Tertile 3	0.254 (0.230,0.277)	0.0375	.	0.294 (0.266,0.322)	0.0778	.
Vitamin A, RAE						
Tertile 1	.	.	.	0.325 (0.300,0.351)	.	0.4903
Tertile 2	.	.	.	0.305 (0.279,0.332)	0.2905	.
Tertile 3	.	.	.	0.306 (0.279,0.334)	0.3197	.
Retinol, mcg						
Tertile 1	0.253 (0.231,0.276)	.	0.0087	0.306 (0.278,0.335)	.	0.5088
Tertile 2	0.297 (0.275,0.319)	0.0067	.	0.324 (0.299,0.350)	0.3498	.
Tertile 3	0.297 (0.274,0.319)	0.0084	.	0.305 (0.279,0.331)	0.9397	.
Vitamin D, mcg						
Tertile 1	0.274 (0.252,0.296)	.	0.0286	0.319 (0.293,0.346)	.	0.3784
Tertile 2	0.266 (0.243,0.289)	0.6002	.	0.320 (0.295,0.345)	0.9684	.
Tertile 3	0.307 (0.285,0.329)	0.0417	.	0.296 (0.268,0.324)	0.2328	.
Vitamin E, mg						
Tertile 1	0.288 (0.264,0.311)	.	0.2738	0.346 (0.320,0.372)	.	<.0001
Tertile 2	0.292 (0.270,0.315)	0.7727	.	0.323 (0.297,0.349)	0.2257	.
Tertile 3	0.268 (0.246,0.290)	0.2329	.	0.264 (0.237,0.291)	<.0001	.
Vitamin C, mg						
Tertile 1	0.291 (0.269,0.312)	.	0.6494	0.337 (0.310,0.363)	.	0.0799
Tertile 2	0.278 (0.255,0.301)	0.4313	.	0.307 (0.282,0.332)	0.1150	.
Tertile 3	0.278 (0.254,0.301)	0.4239	.	0.294 (0.266,0.322)	0.0295	.
Thiamine, mg						
Tertile 1	0.290 (0.269,0.310)	.	0.4312	0.313 (0.286,0.340)	.	0.9905
Tertile 2	0.287 (0.262,0.311)	0.8517	.	0.313 (0.287,0.339)	0.9948	.
Tertile 3	0.270 (0.247,0.293)	0.2159	.	0.311 (0.284,0.338)	0.9084	.
Riboflavin, mg						
Tertile 1	0.275 (0.253,0.297)	.	0.6535	0.320 (0.294,0.346)	.	0.7503
Tertile 2	0.290 (0.268,0.312)	0.3565	.	0.312 (0.285,0.339)	0.6895	.
Tertile 3	0.282 (0.258,0.306)	0.6714	.	0.305 (0.279,0.332)	0.4489	.
Niacin, mg						
Tertile 1	0.284 (0.263,0.305)	.	0.0606	0.321 (0.295,0.347)	.	0.6168
Tertile 2	0.300 (0.278,0.323)	0.3002	.	0.314 (0.288,0.340)	0.7221	.
Tertile 3	0.260 (0.236,0.284)	0.1507	.	0.302 (0.274,0.329)	0.3298	.
Vitamin B6, mg						
Tertile 1	0.276 (0.255,0.298)	.	0.3597	0.325 (0.298,0.351)	.	0.0039
Tertile 2	0.297 (0.273,0.321)	0.2006	.	0.334 (0.308,0.359)	0.6290	.
Tertile 3	0.276 (0.253,0.299)	0.9902	.	0.273 (0.245,0.301)	0.0086	.
Folate, mcg						
Tertile 1	0.293 (0.273,0.314)	.	0.1786	0.357 (0.330,0.383)	.	<.0001
Tertile 2	0.266 (0.243,0.288)	0.0745	.	0.307 (0.282,0.332)	0.0073	.
Tertile 3	0.289 (0.263,0.314)	0.7657	.	0.272 (0.243,0.300)	<.0001	.
Natural Food Folate						

Tertile 1	.	.	.	0.343 (0.316,0.370)	.	0.0153
Tertile 2	.	.	.	0.309 (0.284,0.335)	0.0724	.
Tertile 3	.	.	.	0.287 (0.260,0.314)	0.0041	.
Folic Acid						
Tertile 1	.	.	.	0.339 (0.314,0.365)	.	0.0222
Tertile 2	.	.	.	0.310 (0.282,0.338)	0.1258	.
Tertile 3	.	.	.	0.288 (0.262,0.314)	0.0060	.
Vitamin B12, mcg						
Tertile 1	0.267 (0.244,0.290)	.	0.2247	0.321 (0.294,0.347)	.	0.5225
Tertile 2	0.295 (0.273,0.317)	0.0841	.	0.317 (0.290,0.343)	0.8295	.
Tertile 3	0.283 (0.260,0.305)	0.3445	.	0.300 (0.273,0.327)	0.2812	.
Beta-carotene, mcg						
Tertile 1	0.294 (0.273,0.314)	.	0.0060	0.325 (0.299,0.351)	.	0.0283
Tertile 2	0.300 (0.277,0.323)	0.6857	.	0.328 (0.302,0.354)	0.8568	.
Tertile 3	0.251 (0.227,0.274)	0.0076	.	0.282 (0.254,0.309)	0.0249	.
Beta-carotene equivalents, mcg						
Tertile 1	0.292 (0.271,0.313)	.	0.0035			
Tertile 2	0.303 (0.280,0.326)	0.4819	.			
Tertile 3	0.249 (0.226,0.273)	0.0086	.			
Alpha-carotene, mcg						
Tertile 1	0.278 (0.255,0.302)	.	0.0006	0.322 (0.295,0.349)	.	0.3586
Tertile 2	0.315 (0.293,0.337)	0.0250	.	0.317 (0.293,0.341)	0.7839	.
Tertile 3	0.254 (0.232,0.276)	0.1300	.	0.295 (0.266,0.324)	0.1765	.
Beta-cryptoxanthin, mcg						
Tertile 1	0.279 (0.259,0.300)	.	0.9055	0.335 (0.308,0.363)	.	0.1544
Tertile 2	0.283 (0.259,0.306)	0.8447	.	0.305 (0.280,0.331)	0.1143	.
Tertile 3	0.287 (0.263,0.311)	0.6559	.	0.300 (0.274,0.327)	0.0748	.
Lutein and zeaxanthin, mcg						
Tertile 1	0.297 (0.277,0.317)	.	0.0020	0.310 (0.284,0.336)	.	0.7461
Tertile 2	0.294 (0.271,0.316)	0.8248	.	0.321 (0.294,0.348)	0.5559	.
Tertile 3	0.242 (0.216,0.268)	0.0010	.	0.307 (0.281,0.334)	0.8869	.
Lycopene, mcg						
Tertile 1	0.320 (0.298,0.342)	.	0.0001	0.288 (0.260,0.316)	.	0.0819
Tertile 2	0.266 (0.244,0.288)	0.0006	.	0.331 (0.305,0.356)	0.0257	.
Tertile 3	0.257 (0.233,0.281)	0.0001	.	0.314 (0.288,0.340)	0.1821	.
Calcium, mg						
Tertile 1	0.286 (0.263,0.309)	.	0.8272	0.337 (0.311,0.364)	.	0.0414
Tertile 2	0.284 (0.263,0.306)	0.9199	.	0.309 (0.284,0.335)	0.1337	.
Tertile 3	0.277 (0.253,0.300)	0.5651	.	0.288 (0.260,0.316)	0.0123	.
Magnesium, mg						
Tertile 1	0.277 (0.256,0.298)	.	0.7244	0.316 (0.288,0.343)	.	0.2126
Tertile 2	0.289 (0.267,0.312)	0.4246	.	0.326 (0.301,0.352)	0.5753	.
Tertile 3	0.282 (0.257,0.306)	0.7777	.	0.293 (0.266,0.320)	0.2569	.
Iron, mg						
Tertile 1	0.308 (0.287,0.329)	.	0.0041	0.342 (0.315,0.368)	.	0.0314
Tertile 2	0.256 (0.234,0.278)	0.0009	.	0.302 (0.276,0.328)	0.0370	.
Tertile 3	0.280 (0.256,0.305)	0.0917	.	0.295 (0.268,0.322)	0.0147	.
Zinc, mg						
Tertile 1	0.283 (0.262,0.305)	.	0.0263	0.318 (0.291,0.345)	.	0.0757
Tertile 2	0.307 (0.282,0.331)	0.1511	.	0.330 (0.304,0.356)	0.5113	.
Tertile 3	0.262 (0.240,0.284)	0.1706	.	0.288 (0.261,0.315)	0.1217	.
Copper, mg						
Tertile 1	0.290 (0.269,0.311)	.	0.5334	0.320 (0.293,0.348)	.	0.2436
Tertile 2	0.284 (0.261,0.307)	0.7056	.	0.323 (0.297,0.348)	0.8917	.

Tertile 3	0.272 (0.248,0.296)	0.2663	.	0.294 (0.267,0.321)	0.1744	.
Selenium, mcg						
Tertile 1	0.308 (0.287,0.330)	.	0.0023	0.301 (0.281,0.321)	.	0.2270
Tertile 2	0.255 (0.234,0.276)	0.0005	.	0.321 (0.259,0.383)	0.5458	.
Tertile 3	0.287 (0.261,0.312)	0.2037	.	0.329 (0.304,0.354)	0.0893	.
Saturated fat, % kcal						
Tertile 1	0.291 (0.268,0.314)	.	0.5660	0.281 (0.253,0.309)	.	0.0184
Tertile 2	0.273 (0.250,0.296)	0.2876	.	0.316 (0.290,0.342)	0.0701	.
Tertile 3	0.283 (0.262,0.305)	0.6275	.	0.335 (0.310,0.361)	0.0050	.
Monounsaturated fat, % kcal						
Tertile 1	0.263 (0.240,0.286)	.	0.1053	0.316 (0.289,0.343)	.	0.9560
Tertile 2	0.297 (0.274,0.321)	0.0398	.	0.310 (0.284,0.336)	0.7653	.
Tertile 3	0.287 (0.265,0.308)	0.1348	.	0.312 (0.285,0.339)	0.8584	.
Cholesterol, mg						
Tertile 1	0.294 (0.272,0.317)	.	0.0380	0.306 (0.279,0.333)	.	0.2692
Tertile 2	0.258 (0.234,0.281)	0.0243	.	0.301 (0.273,0.328)	0.7724	.
Tertile 3	0.293 (0.271,0.316)	0.9513	.	0.330 (0.304,0.355)	0.2170	.
Oleic acid, mg per 1000 kcal						
Tertile 1	0.260 (0.237,0.283)	.	0.0589	0.320 (0.292,0.347)	.	0.6898
Tertile 2	0.298 (0.275,0.321)	0.0227	.	0.315 (0.289,0.341)	0.8080	.
Tertile 3	0.288 (0.267,0.310)	0.0771	.	0.303 (0.276,0.330)	0.4050	.
Linoleic acid, mg per 1000 kcal						
Tertile 1	0.287 (0.263,0.311)	.	0.6560	0.303 (0.274,0.331)	.	0.0161
Tertile 2	0.288 (0.264,0.312)	0.9538	.	0.343 (0.317,0.369)	0.0400	.
Tertile 3	0.275 (0.254,0.296)	0.4538	.	0.292 (0.267,0.317)	0.5801	.
a-Linolenic acid, mg per 1000 kcal						
Tertile 1	0.277 (0.254,0.301)	.	0.1170	0.314 (0.287,0.342)	.	0.0049
Tertile 2	0.304 (0.280,0.328)	0.1212	.	0.347 (0.319,0.375)	0.1010	.
Tertile 3	0.271 (0.251,0.292)	0.7118	.	0.285 (0.260,0.310)	0.1147	.
EPA, mg per 1000 kcal						
Tertile 1	0.287 (0.266,0.308)	.	0.7592	0.313 (0.287,0.339)	.	0.3015
Tertile 2	0.276 (0.253,0.298)	0.4681	.	0.327 (0.300,0.353)	0.4728	.
Tertile 3	0.284 (0.259,0.309)	0.8582	.	0.297 (0.270,0.324)	0.3984	.
DHA, mg per 1000 kcal						
Tertile 1	0.295 (0.274,0.316)	.	0.0728	0.307 (0.280,0.333)	.	0.0857
Tertile 2	0.287 (0.265,0.308)	0.5811	.	0.333 (0.308,0.357)	0.1607	.
Tertile 3	0.257 (0.232,0.283)	0.0259	.	0.291 (0.262,0.320)	0.4341	.
DPA, mg per 1000 kcal						
Tertile 1	.	.	.	0.320 (0.293,0.347)	.	0.4679
Tertile 2	.	.	.	0.318 (0.293,0.343)	0.9228	.
Tertile 3	.	.	.	0.298 (0.270,0.326)	0.2671	.
EPA+DHA, mg per 1000 kcal						
Tertile 1	0.291 (0.271,0.312)	.	0.5193	0.313 (0.286,0.340)	.	0.3768
Tertile 2	0.278 (0.256,0.301)	0.4034	.	0.324 (0.299,0.350)	0.5572	.
Tertile 3	0.273 (0.248,0.299)	0.2859	.	0.298 (0.270,0.325)	0.4259	.
EPA+DPA+DHA, mg per 1000 kcal						
Tertile 1	.	.	.	0.308 (0.282,0.334)	.	0.2544
Tertile 2	.	.	.	0.328 (0.303,0.352)	0.2770	.
Tertile 3	.	.	.	0.296 (0.266,0.326)	0.5547	.
Arachidonic acid, mg per 1000 kcal						
Tertile 1	0.304 (0.282,0.325)	.	0.0119	0.301 (0.276,0.327)	.	0.5737
Tertile 2	0.256 (0.232,0.279)	0.0029	.	0.317 (0.290,0.344)	0.4106	.
Tertile 3	0.282 (0.258,0.305)	0.1738	.	0.320 (0.293,0.347)	0.3307	.
Galactose, gm						

Tertile 1	0.295 (0.274,0.316)	.	0.2059	.	.	.
Tertile 2	0.282 (0.260,0.305)	0.4337	.	.	.	.
Tertile 3	0.266 (0.242,0.290)	0.0755	.	.	.	.
Lactose, gm						
Tertile 1	0.287 (0.264,0.310)	.	0.6870	0.296 (0.268,0.323)	.	0.1245
Tertile 2	0.274 (0.252,0.297)	0.4416	.	0.334 (0.308,0.360)	0.0504	.
Tertile 3	0.286 (0.264,0.309)	0.9667	.	0.306 (0.280,0.332)	0.5930	.
Alcohol, gm						
Tertile 1	0.297 (0.278,0.316)	.	0.0886	0.351 (0.324,0.378)	.	0.0033
Tertile 2	0.264 (0.237,0.291)	0.0462	.	0.292 (0.266,0.317)	0.0016	.
Tertile 3	0.272 (0.248,0.297)	0.1190	.	0.299 (0.272,0.326)	0.0073	.
Soluble Dietary Fiber, gm						
Tertile 1	0.293 (0.271,0.314)	.	0.1413	.	.	.
Tertile 2	0.286 (0.265,0.307)	0.6676	.	.	.	.
Tertile 3	0.259 (0.233,0.286)	0.0552	.	.	.	.
Insoluble Dietary Fiber, gm						
Tertile 1	0.290 (0.269,0.311)	.	0.6897	.	.	.
Tertile 2	0.278 (0.257,0.300)	0.4598	.	.	.	.
Tertile 3	0.277 (0.252,0.303)	0.4688	.	.	.	.
Fiber (Soluble or Insoluble), gm						
Tertile 1	.	.	.	0.326 (0.299,0.352)	.	0.3264
Tertile 2	.	.	.	0.314 (0.289,0.340)	0.5552	.
Tertile 3	.	.	.	0.296 (0.268,0.324)	0.1374	.
Total Choline						
Tertile 1	.	.	.	0.300 (0.274,0.327)	.	0.2403
Tertile 2	.	.	.	0.331 (0.304,0.358)	0.1077	.
Tertile 3	.	.	.	0.307 (0.280,0.334)	0.7391	.
Free Choline						
Tertile 1	.	.	.	0.334 (0.308,0.361)	.	0.1168
Tertile 2	.	.	.	0.304 (0.279,0.330)	0.1050	.
Tertile 3	.	.	.	0.296 (0.268,0.325)	0.0551	.
Glycemic Index						
Tertile 1	.	.	.	0.313 (0.287,0.339)	.	0.3346
Tertile 2	.	.	.	0.326 (0.300,0.353)	0.4808	.
Tertile 3	.	.	.	0.298 (0.270,0.325)	0.4214	.
Glycemic Load						
Tertile 1	.	.	.	0.318 (0.292,0.344)	.	0.8119
Tertile 2	.	.	.	0.313 (0.286,0.340)	0.8033	.
Tertile 3	.	.	.	0.306 (0.279,0.333)	0.5213	.

Abbreviations: AREDS=Age-Related Eye Diseases Study; CI=confidence interval; DHA=docosahexaenoic acid; DPA=docosapentaenoic acid; EPA=eicosapentaenoic acid; IU=international units; RAE=retinol activity equivalents

Table S6. Geographic Atrophy Proximity-Based Progression Rates, according to Quantiles of the Alternative Mediterranean Dietary Index and its Components, in the Age-Related Eye Diseases Studies 1 and 2.

	AREDS (n = 390 eyes of 328 participants)			AREDS2 (n = 826 eyes of 652 participants)		
	Estimate, µm/year (CI)	Pairwise P Value	Interaction P Value	Estimate, µm/year (CI)	Pairwise P Value	Interaction P Value
aMedi						
Tertile 1	54.5 (46.4,62.7)	.	0.0235	93.7 (81.2,106.2)	.	0.2090
Tertile 2	54.9 (47.1,62.6)	0.9539	.	99.2 (85.8,112.7)	0.5565	.
Tertile 3	39.9 (31.0,48.9)	0.0182	.	82.3 (68.6,95.9)	0.2252	.
Whole fruit					.	.
Quartile 1	51.9 (41.9,61.9)	.	0.0001	98.0 (82.0,114.1)	.	0.6965
Quartile 2	67.8 (58.3,77.3)	0.0245	.	85.5 (71.5,99.5)	0.2469	.
Quartile 3	38.5 (29.7,47.3)	0.0478	.	93.5 (78.4,108.7)	0.6896	.
Quartile 4	46.5 (36.9,56.0)	0.4371	.	93.0 (77.1,108.9)	0.6632	.
Vegetables					.	.
Quartile 1	61.8 (53.0,70.5)	.	<.0001	90.5 (75.2,105.9)	.	0.9561
Quartile 2	62.0 (52.3,71.7)	0.9670	.	91.5 (77.0,105.9)	0.9319	.
Quartile 3	35.6 (26.7,44.5)	<.0001	.	95.5 (80.6,110.5)	0.6474	.
Quartile 4	41.8 (31.0,52.6)	0.0049	.	89.9 (73.6,106.2)	0.9527	.
Whole grains					.	.
Quartile 1	51.7 (42.2,61.2)	.	0.0142	99.2 (82.1,116.3)	.	0.2395
Quartile 2	39.6 (30.1,49.0)	0.0759	.	98.2 (83.9,112.4)	0.9262	.
Quartile 3	63.3 (52.5,74.0)	0.1130	.	79.4 (64.7,94.2)	0.0855	.
Quartile 4	50.7 (42.1,59.3)	0.8795	.	92.5 (77.3,107.7)	0.5639	.
Nuts					.	.
Quartile 1	56.1 (46.9,65.3)	.	0.0329	89.4 (75.1,103.7)	.	0.0496
Quartile 2	63.2 (49.9,76.6)	0.3875	.	81.7 (65.8,97.7)	0.4836	.
Quartile 3	42.0 (33.4,50.6)	0.0281	.	109.9 (95.0,124.9)	0.0515	.
Quartile 4	49.3 (40.6,57.9)	0.2869	.	85.9 (70.2,101.5)	0.7472	.
Legumes					.	.
Quartile 1	54.1 (44.5,63.7)	.	0.0092	89.5 (71.5,107.4)	.	0.0676
Quartile 2	63.0 (53.2,72.9)	0.2027	.	102.7 (87.4,118.0)	0.2712	.
Quartile 3	44.2 (35.8,52.6)	0.1268	.	96.6 (83.9,109.3)	0.5238	.
Quartile 4	41.9 (31.3,52.5)	0.0938	.	74.0 (57.6,90.3)	0.2107	.
Red meat*					.	.
Quartile 1	51.2 (42.9,59.5)	.	0.0335	101.6 (87.1,116.2)	.	0.2461
Quartile 2	46.8 (37.9,55.8)	0.4813	.	94.0 (78.9,109.1)	0.4768	.
Quartile 3	65.4 (53.8,76.9)	0.0509	.	79.4 (63.2,95.5)	0.0450	.
Quartile 4	44.0 (34.0,53.9)	0.2707	.	90.2 (75.1,105.3)	0.2850	.
Fish					.	.
Quartile 1	46.3 (36.6,55.9)	.	0.0016	86.3 (69.2,103.4)	.	0.3325
Quartile 2	67.6 (57.8,77.4)	0.0023	.	86.2 (73.6,98.9)	0.9948	.
Quartile 3	44.0 (36.1,51.9)	0.7252	.	103.0 (88.2,117.9)	0.1476	.
Quartile 4	48.0 (36.4,59.6)	0.8198	.	94.4 (76.5,112.3)	0.5225	.
MUFA: SFA					.	.
Quartile 1	33.9 (24.0,43.7)	.	0.0004	99.2 (83.8,114.5)	.	0.1567
Quartile 2	57.6 (47.7,67.5)	0.0009	.	89.2 (74.9,103.5)	0.3508	.
Quartile 3	62.5 (52.6,72.4)	<.0001	.	102.0 (86.1,118.0)	0.7992	.
Quartile 4	49.4 (41.0,57.9)	0.0188	.	79.2 (63.9,94.4)	0.0704	.
Alcohol					.	.
In interval	41.1 (23.9,58.2)	0.2508	.	57.1 (34.6,79.5)	.	.

Not in interval	51.5 (46.6,56.5)	.	0.2508	96.3 (88.3,104.4)	0.0013	0.0013

Abbreviations: aMedi=Alternative Mediterranean Diet Index; AREDS=Age-Related Eye Diseases Study; CI=confidence interval; MUFA: SFA=monounsaturated fatty acid: saturated fatty acid

* For red meat, higher quartiles refer to lower levels of intake, which is more adherent to the Alternative Mediterranean Diet Index

Table S7. Geographic Atrophy Proximity-Based Progression Rates, according to Quantiles of Nutrient Intake, in the Age-Related Eye Diseases Studies 1 and 2.

	AREDS (n = 390 eyes of 328 participants)			AREDS2 (n = 826 eyes of 652 participants)		
Nutrient	Estimate, µm/year (CI)	Pairwise P Value	Interaction P Value	Estimate, µm/year (CI)	Pairwise P Value	Interaction P Value
Vitamin A, IU						
Tertile 1	56.9 (49.7,64.2)	.	0.0036	90.8 (77.9,103.7)	.	0.1786
Tertile 2	55.9 (46.1,65.7)	0.8648	.	100.9 (87.9,113.9)	0.2771	.
Tertile 3	39.5 (31.4,47.6)	0.0017	.	83.2 (69.5,96.9)	0.4290	.
Vitamin A, RAE						
Tertile 1	.	.	.	85.5 (73.0,98.0)	.	0.0770
Tertile 2	.	.	.	105.1 (91.4,118.8)	0.0386	.
Tertile 3	.	.	.	86.8 (73.4,100.2)	0.8928	.
Retinol, mcg						
Tertile 1	43.0 (35.3,50.8)	.	0.0321	88.8 (74.4,103.3)	.	0.6583
Tertile 2	53.0 (45.5,60.6)	0.0699	.	90.2 (78.4,102.0)	0.8852	.
Tertile 3	59.0 (49.2,68.8)	0.0123	.	97.3 (83.5,111.1)	0.4073	.
Vitamin D, mcg						
Tertile 1	50.2 (42.3,58.0)	.	0.6442	98.0 (84.9,111.1)	.	0.5279
Tertile 2	48.2 (40.0,56.4)	0.7358	.	89.4 (76.8,102.1)	0.3535	.
Tertile 3	53.9 (45.1,62.6)	0.5348	.	88.1 (74.2,102.0)	0.3080	.
Vitamin E, mg						
Tertile 1	50.2 (41.8,58.6)	.	0.9924	96.3 (83.8,108.8)	.	0.0228
Tertile 2	50.9 (43.0,58.8)	0.9030	.	103.2 (89.3,117.2)	0.4657	.
Tertile 3	50.7 (42.2,59.2)	0.9367	.	77.7 (64.6,90.7)	0.0435	.
Vitamin C, mg						
Tertile 1	51.6 (43.6,59.6)	.	0.8067	87.6 (74.2,101.0)	.	0.6599
Tertile 2	48.4 (40.4,56.5)	0.5903	.	96.0 (83.9,108.1)	0.3650	.
Tertile 3	52.0 (43.3,60.7)	0.9429	.	91.3 (77.1,105.6)	0.7088	.
Thiamine, mg						
Tertile 1	46.0 (38.8,53.2)	.	0.2332	101.8 (87.8,115.8)	.	0.1303
Tertile 2	53.5 (43.9,63.2)	0.2211	.	93.3 (80.6,106.0)	0.3771	.
Tertile 3	54.9 (46.5,63.2)	0.1164	.	82.4 (69.5,95.3)	0.0451	.
Riboflavin, mg						
Tertile 1	43.3 (35.7,50.9)	.	0.0296	97.3 (83.8,110.9)	.	0.1436
Tertile 2	53.2 (45.6,60.8)	0.0704	.	97.6 (84.7,110.5)	0.9748	.
Tertile 3	59.5 (49.4,69.7)	0.0116	.	81.4 (68.4,94.4)	0.0963	.
Niacin, mg						
Tertile 1	45.6 (38.4,52.8)	.	0.0670	89.8 (76.4,103.1)	.	0.2141
Tertile 2	50.2 (41.6,58.7)	0.4270	.	100.7 (87.8,113.5)	0.2488	.
Tertile 3	59.7 (50.3,69.1)	0.0203	.	84.5 (71.2,97.8)	0.5830	.
Vitamin B6, mg						
Tertile 1	42.4 (35.2,49.6)	.	0.0057	94.6 (81.7,107.6)	.	0.0264
Tertile 2	52.8 (43.8,61.7)	0.0764	.	103.5 (90.1,116.9)	0.3531	.
Tertile 3	60.9 (52.0,69.8)	0.0016	.	78.1 (65.0,91.3)	0.0789	.
Folate, mcg						
Tertile 1	43.8 (36.1,51.5)	.	0.0687	99.8 (86.5,113.2)	.	0.3578
Tertile 2	53.2 (45.3,61.1)	0.0954	.	87.1 (74.7,99.6)	0.1714	.
Tertile 3	57.2 (47.9,66.6)	0.0295	.	89.4 (75.6,103.2)	0.2860	.
Natural Food Folate						
Tertile 1	.	.	.	89.9 (76.4,103.3)	.	0.3440
Tertile 2	.	.	.	99.4 (86.5,112.3)	0.3148	.
Tertile 3	.	.	.	86.1 (72.9,99.3)	0.6968	.

Folic Acid						
Tertile 1	.	.	.	99.3 (85.4,113.1)	.	0.4151
Tertile 2	.	.	.	86.4 (73.1,99.7)	0.1897	.
Tertile 3	.	.	.	91.0 (78.6,103.4)	0.3846	.
Vitamin B12, mcg						
Tertile 1	39.9 (32.1,47.7)	.	0.0030	103.1 (89.6,116.6)	.	0.0520
Tertile 2	55.7 (48.0,63.3)	0.0048	.	80.5 (67.9,93.1)	0.0166	.
Tertile 3	58.7 (49.2,68.2)	0.0028	.	94.4 (81.0,107.8)	0.3718	.
Beta-carotene, mcg						
Tertile 1	58.6 (50.8,66.4)	.	0.0107	92.3 (79.8,104.8)	.	0.9018
Tertile 2	51.4 (42.8,60.0)	0.2243	.	93.8 (80.4,107.2)	0.8768	.
Tertile 3	41.1 (32.9,49.4)	0.0026	.	89.4 (75.7,103.1)	0.7575	.
Beta-carotene equivalents, mcg						
Tertile 1	60.3 (52.5,68.2)	.	0.0022	.	.	.
Tertile 2	50.7 (42.1,59.2)	0.1016	.	.	.	.
Tertile 3	40.0 (31.7,48.3)	0.0005	.	.	.	.
Alpha-carotene, mcg						
Tertile 1	67.2 (59.0,75.4)	.	<.0001	93.3 (79.9,106.6)	.	0.1594
Tertile 2	42.2 (33.6,50.7)	<.0001	.	99.1 (86.8,111.3)	0.5295	.
Tertile 3	43.1 (35.3,50.9)	<.0001	.	81.0 (66.9,95.1)	0.2151	.
Beta-cryptoxanthin, mcg						
Tertile 1	50.3 (42.2,58.4)	.	0.9927	105.7 (92.3,119.1)	.	0.0175
Tertile 2	50.5 (42.5,58.6)	0.9691	.	79.2 (66.6,91.7)	0.0045	.
Tertile 3	51.0 (42.4,59.7)	0.9055	.	92.8 (79.3,106.4)	0.1857	.
Lutein and zeaxanthin, mcg						
Tertile 1	55.1 (47.8,62.4)	.	0.0172	84.0 (71.6,96.5)	.	0.1306
Tertile 2	54.2 (45.7,62.7)	0.8701	.	90.5 (76.9,104.1)	0.4947	.
Tertile 3	39.1 (29.8,48.3)	0.0076	.	102.8 (89.3,116.3)	0.0455	.
Lycopene, mcg						
Tertile 1	64.5 (56.0,73.0)	.	0.0005	83.3 (69.0,97.5)	.	0.1043
Tertile 2	42.4 (35.0,49.8)	0.0001	.	102.2 (89.8,114.7)	0.0492	.
Tertile 3	47.5 (38.6,56.4)	0.0069	.	87.4 (74.5,100.4)	0.6717	.
Calcium, mg						
Tertile 1	48.7 (40.8,56.6)	.	0.0578	100.6 (87.5,113.7)	.	0.2667
Tertile 2	57.7 (49.9,65.6)	0.1104	.	88.7 (75.7,101.8)	0.2080	.
Tertile 3	43.6 (34.4,52.7)	0.4027	.	86.1 (72.8,99.5)	0.1292	.
Magnesium, mg						
Tertile 1	44.8 (37.6,52.0)	.	0.0350	103.1 (89.2,117.0)	.	0.1615
Tertile 2	59.7 (51.0,68.4)	0.0099	.	88.8 (76.9,100.8)	0.1278	.
Tertile 3	49.6 (40.4,58.8)	0.4194	.	85.1 (71.2,99.0)	0.0736	.
Iron, mg						
Tertile 1	47.9 (40.0,55.7)	.	0.1812	97.6 (84.1,111.1)	.	0.4886
Tertile 2	48.2 (40.6,55.8)	0.9533	.	92.5 (79.5,105.5)	0.5946	.
Tertile 3	58.5 (48.8,68.1)	0.0947	.	86.1 (73.1,99.2)	0.2331	.
Zinc, mg						
Tertile 1	48.4 (41.4,55.5)	.	0.5361	93.7 (80.0,107.4)	.	0.0017
Tertile 2	49.8 (40.0,59.5)	0.8283	.	106.4 (93.9,118.9)	0.1776	.
Tertile 3	54.6 (46.1,63.2)	0.2726	.	73.4 (60.2,86.7)	0.0373	.
Copper, mg						
Tertile 1	48.1 (40.5,55.8)	.	0.7178	80.5 (67.0,94.1)	.	0.0006
Tertile 2	52.2 (43.7,60.7)	0.4861	.	111.8 (99.2,124.4)	0.0009	.
Tertile 3	52.2 (43.5,60.8)	0.4931	.	80.8 (67.6,94.0)	0.9784	.
Selenium, mcg						
Tertile 1	39.8 (32.4,47.2)	.	0.0001	89.4 (79.0,99.8)	.	0.7679

Tertile 2	63.5 (55.6,71.3)	<.0001	.	96.2 (64.1,128.2)	0.6924	.
Tertile 3	49.6 (39.9,59.4)	0.1168	.	94.8 (82.9,106.7)	0.4983	.
Saturated fat, % kcal						
Tertile 1	46.7 (39.0,54.4)	.	0.0043	87.2 (73.7,100.6)	.	0.4106
Tertile 2	62.7 (54.1,71.3)	0.0067	.	89.4 (76.4,102.5)	0.8134	.
Tertile 3	44.0 (35.6,52.4)	0.6449	.	99.1 (86.1,112.1)	0.2128	.
Monounsaturated fat, % kcal						
Tertile 1	48.1 (39.9,56.3)	.	0.0020	92.8 (79.8,105.9)	.	0.9124
Tertile 2	64.6 (55.3,73.8)	0.0089	.	93.4 (80.0,106.8)	0.9528	.
Tertile 3	43.7 (36.2,51.2)	0.4363	.	89.6 (76.5,102.7)	0.7341	.
Cholesterol, mg						
Tertile 1	53.8 (45.8,61.7)	.	0.2812	91.6 (78.4,104.8)	.	0.1271
Tertile 2	45.5 (37.6,53.5)	0.1500	.	82.1 (68.8,95.5)	0.3238	.
Tertile 3	53.3 (44.3,62.3)	0.9434	.	101.4 (88.5,114.3)	0.3004	.
Oleic acid, mg per 1000 kcal						
Tertile 1	45.1 (36.7,53.5)	.	<.0001	93.9 (80.9,107.0)	.	0.6092
Tertile 2	67.3 (58.5,76.0)	0.0003	.	95.4 (82.0,108.7)	0.8791	.
Tertile 3	43.0 (35.5,50.6)	0.7257	.	86.6 (73.5,99.7)	0.4351	.
Linoleic acid, mg per 1000 kcal						
Tertile 1	52.1 (43.5,60.6)	.	0.5856	89.7 (75.5,103.9)	.	0.3406
Tertile 2	53.3 (44.3,62.3)	0.8450	.	99.8 (86.7,112.9)	0.3059	.
Tertile 3	47.7 (40.2,55.1)	0.4441	.	86.7 (74.3,99.1)	0.7583	.
a-Linolenic acid, mg per 1000 kcal						
Tertile 1	51.6 (43.7,59.5)	.	<.0001	100.9 (87.5,114.4)	.	<.0001
Tertile 2	67.6 (58.3,76.8)	0.0102	.	110.4 (96.1,124.6)	0.3447	.
Tertile 3	38.5 (30.9,46.0)	0.0190	.	72.0 (60.1,84.0)	0.0017	.
EPA, mg per 1000 kcal						
Tertile 1	57.4 (50.1,64.8)	.	0.0131	84.0 (71.3,96.6)	.	0.0034
Tertile 2	41.2 (33.2,49.2)	0.0035	.	110.1 (97.1,123.2)	0.0048	.
Tertile 3	52.3 (42.5,62.1)	0.4163	.	81.2 (67.4,95.0)	0.7709	.
DHA, mg per 1000 kcal						
Tertile 1	50.5 (42.2,58.7)	.	0.3192	82.5 (69.5,95.4)	.	0.0008
Tertile 2	54.1 (46.7,61.5)	0.5180	.	110.5 (98.2,122.9)	0.0021	.
Tertile 3	44.9 (35.4,54.3)	0.3803	.	78.2 (63.9,92.6)	0.6683	.
DPA, mg per 1000 kcal						
Tertile 1	.	.	.	88.8 (75.6,102.0)	.	0.0597
Tertile 2	.	.	.	104.1 (91.1,117.0)	0.1058	.
Tertile 3	.	.	.	82.2 (68.9,95.5)	0.4912	.
EPA+DHA, mg per 1000 kcal						
Tertile 1	58.4 (50.2,66.6)	.	0.0351	80.8 (67.9,93.7)	.	0.0008
Tertile 2	44.0 (36.7,51.2)	0.0098	.	111.5 (98.8,124.2)	0.0009	.
Tertile 3	51.3 (41.6,61.1)	0.2768	.	81.4 (67.4,95.3)	0.9536	.
EPA+DPA+DHA, mg per 1000 kcal						
Tertile 1	.	.	.	79.4 (67.0,91.9)	.	0.0010
Tertile 2	.	.	.	110.4 (98.1,122.8)	0.0005	.
Tertile 3	.	.	.	82.6 (67.5,97.7)	0.7526	.
Arachidonic acid, mg per 1000 kcal						
Tertile 1	53.3 (45.2,61.4)	.	0.5124	93.2 (80.1,106.4)	.	0.0030
Tertile 2	47.1 (39.4,54.8)	0.2790	.	76.0 (63.1,88.8)	0.0659	.
Tertile 3	52.2 (43.1,61.4)	0.8689	.	108.3 (94.9,121.7)	0.1162	.
Galactose, gm						
Tertile 1	57.8 (50.3,65.4)	.	0.0178	.	.	.
Tertile 2	51.1 (42.2,59.9)	0.2544	.	.	.	.
Tertile 3	41.5 (33.2,49.9)	0.0046	.	.	.	.

Lactose, gm						
Tertile 1	53.4 (45.4,61.4)	.	0.0733	94.2 (80.4,107.9)	.	0.5830
Tertile 2	54.8 (46.9,62.6)	0.8133	.	95.2 (82.5,107.9)	0.9130	.
Tertile 3	41.9 (33.0,50.8)	0.0595	.	86.2 (73.1,99.4)	0.4129	.
Alcohol, gm						
Tertile 1	48.9 (41.9,55.8)	.	0.2599	104.9 (91.5,118.4)	.	0.0342
Tertile 2	47.3 (38.2,56.5)	0.7968	.	90.9 (78.6,103.2)	0.1305	.
Tertile 3	57.3 (47.9,66.7)	0.1553	.	79.4 (65.6,93.3)	0.0096	.
Soluble Dietary Fiber, gm						
Tertile 1	45.3 (36.9,53.6)	.	0.0681	.	.	.
Tertile 2	57.9 (50.0,65.8)	0.0308	.	.	.	.
Tertile 3	47.6 (39.1,56.0)	0.7046	.	.	.	.
Insoluble Dietary Fiber, gm						
Tertile 1	44.0 (35.8,52.2)	.	0.0222	.	.	.
Tertile 2	58.9 (51.3,66.6)	0.0092	.	.	.	.
Tertile 3	47.1 (38.2,56.0)	0.6204	.	.	.	.
Fiber (Soluble or Insoluble), gm						
Tertile 1	.	.	.	88.4 (75.4,101.4)	.	0.0922
Tertile 2	.	.	.	103.4 (90.4,116.5)	0.1108	.
Tertile 3	.	.	.	83.5 (70.1,96.9)	0.6040	.
Total Choline						
Tertile 1	.	.	.	87.6 (74.3,100.8)	.	0.2703
Tertile 2	.	.	.	100.5 (87.7,113.3)	0.1685	.
Tertile 3	.	.	.	87.2 (73.7,100.7)	0.9689	.
Free Choline						
Tertile 1	.	.	.	104.1 (90.8,117.3)	.	0.0442
Tertile 2	.	.	.	91.1 (78.5,103.8)	0.1659	.
Tertile 3	.	.	.	80.0 (66.4,93.5)	0.0127	.
Glycemic Index						
Tertile 1	.	.	.	90.6 (77.6,103.6)	.	0.8797
Tertile 2	.	.	.	94.6 (81.7,107.4)	0.6729	.
Tertile 3	.	.	.	90.3 (76.6,104.0)	0.9701	.
Glycemic Load						
Tertile 1	.	.	.	84.5 (71.9,97.1)	.	0.2500
Tertile 2	.	.	.	92.0 (77.9,106.0)	0.4372	.
Tertile 3	.	.	.	99.8 (86.9,112.8)	0.0959	.

Abbreviations: AREDS=Age-Related Eye Diseases Study; CI=confidence interval; DHA=docosahexaenoic acid; DPA=docosapentaenoic acid; EPA=eicosapentaenoic acid; IU=international units; RAE=retinol activity equivalents

Table S8. Geographic Atrophy Proximity-Based Progression Rates, according to Quantiles of the Alternative Mediterranean Dietary Index and its Components, separately according to Randomized Oral Supplement Assignments, in the Age-Related Eye Diseases Studies 1 and 2.

	AREDS						AREDS2					
	Antioxidants			No Antioxidants			Lutein/Zeaxanthin			No Lutein/Zeaxanthin		
	Estimate, µm/year (CI)	Pairwise P Value	Interaction P Value	Estimate, µm/year (CI)	Pairwise P Value	Interaction P Value	Estimate, µm/year (CI)	Pairwise P Value	Interaction P Value	Estimate, µm/year (CI)	Pairwise P Value	Interaction P Value
aMedi												
Tertile 1	53.4 (36.1,70.7)	.	0.8654	91.7 (73.5,110.0)	.	0.0014	79.5 (60.6,98.4)	.	0.9321	103.8 (87.1,120.5)	.	0.0182
Tertile 2	46.6 (28.4,64.7)	0.5914	.	54.8 (35.7,73.9)	0.0063	.	82.0 (63.2,100.9)	0.8513	.	118.9 (99.6,138.1)	0.2455	.
Tertile 3	49.8 (16.0,83.5)	0.8515	.	32.9 (2.10,63.6)	0.0013	.	84.6 (65.8,103.3)	0.7077	.	79.1 (59.2,99.1)	0.0632	.
Whole fruit												
Quartile 1	40.5 (19.5,61.4)	.	<.0001	75.6 (53.4,97.7)	.	0.0204	93.5 (69.5,117.5)	.	0.0739	100.9 (79.3,122.5)	.	0.6095
Quartile 2	67.3 (43.6,91.1)	0.0956	.	85.4 (63.7,107.1)	0.5314	.	62.6 (42.3,82.9)	0.0538	.	104.7 (85.2,124.1)	0.8013	.
Quartile 3	20.5 (0.0,41.1)	0.1810	.	66.9 (37.0,96.7)	0.6452	.	99.0 (77.7,120.4)	0.7361	.	89.9 (68.4,111.4)	0.4767	.
Quartile 4	94.9 (69.6,120.2)	0.0013	.	29.5 (0.90,58.0)	0.0127	.	78.6 (57.1,100.1)	0.3649	.	110.9 (87.1,134.7)	0.5444	.
Vegetables												
Quartile 1	66.8 (45.2,88.5)	.	0.0577	99.8 (78.4,121.2)	.	0.0015	88.4 (65.9,110.9)	.	0.8188	91.0 (70.0,112.1)	.	0.6866
Quartile 2	47.6 (22.6,72.7)	0.2541	.	56.8 (34.4,79.3)	0.0067	.	82.6 (62.5,102.6)	0.7042	.	102.2 (81.1,123.3)	0.4627	.
Quartile 3	35.6 (17.3,53.8)	0.0305	.	68.7 (41.4,95.9)	0.0778	.	84.8 (62.4,107.2)	0.8253	.	104.2 (84.0,124.4)	0.3764	.
Quartile 4	82.4 (44.5,120.3)	0.4827	.	32.2 (4.3,60.0)	0.0002	.	73.5 (51.2,95.8)	0.3573	.	109.7 (85.8,133.7)	0.2495	.
Whole grains												
Quartile 1	41.1 (18.4,63.8)	.	0.6015	86.1 (59.7,112.5)	.	0.0018	80.9 (55.6,106.2)	.	0.0502	114.9 (91.6,138.1)	.	0.2937
Quartile 2	61.8 (39.1,84.6)	0.2046	.	60.0 (35.9,84.2)	0.1507	.	91.2 (69.2,113.1)	0.5470	.	102.5 (83.8,121.2)	0.4147	.
Quartile 3	52.0 (29.3,74.7)	0.5047	.	90.9 (68.9,112.8)	0.7835	.	61.3 (41.9,80.7)	0.2276	.	105.1 (82.3,127.9)	0.5548	.
Quartile 4	44.2 (17.8,70.7)	0.8606	.	27.4 (0.7,54.2)	0.0023	.	100.0 (78.8,121.2)	0.2566	.	84.5 (62.8,106.3)	0.0618	.
Nuts												
Quartile 1	43.9 (26.2,61.6)	.	0.1383	106.3 (81.4,131.3)	.	0.0012	81.7 (62.8,100.6)	.	0.1761	99.2 (77.1,121.4)	.	0.0124
Quartile 2	86.5 (53.9,119.2)	0.0246	.	83.6 (49.8,117.4)	0.2882	.	62.7 (40.3,85.1)	0.2036	.	101.5 (78.8,124.2)	0.8890	.
Quartile 3	50.0 (21.6,78.3)	0.7226	.	49.7 (30.8,68.5)	0.0004	.	90.3 (68.3,112.3)	0.5575	.	127.0 (106.5,147.5)	0.0719	.
Quartile 4	44.4 (21.8,67.0)	0.9753	.	48.2 (24.4,72.0)	0.0010	.	97.7 (72.9,122.6)	0.3132	.	78.8 (58.7,98.8)	0.1794	.
Legumes												
Quartile 1	51.5 (31.3,71.7)	.	0.9874	67.5 (44.2,90.8)	.	<.0001	56.8 (31.1,82.5)	.	0.0755	120.5 (95.1,145.9)	.	0.0657
Quartile 2	51.0 (30.0,72.0)	0.9721	.	114.0 (92.9,135.1)	0.0039	.	96.2 (72.9,119.4)	0.0260	.	108.1 (87.6,128.6)	0.4549	.
Quartile 3	44.9 (13.6,76.2)	0.7257	.	23.6 (5.3,41.8)	0.0038	.	92.2 (73.9,110.5)	0.0277	.	100.7 (83.1,118.3)	0.2089	.
Quartile 4	50.2 (24.3,76.1)	0.9357	.	136.4 (99.9,173.0)	0.0019	.	73.7 (52.1,95.3)	0.3242	.	74.0 (49.1,99.0)	0.0106	.
Red meat*												
Quartile 1	57.1 (35.1,79.1)	.	0.0060	61.9 (43.4,80.5)	.	0.6945	86.2 (62.8,109.7)	.	0.7372	111.1 (92.5,129.7)	.	0.0051
Quartile 2	41.2 (21.8,60.7)	0.2873	.	65.1 (39.3,91.0)	0.8420	.	74.0 (54.4,93.7)	0.4331	.	124.3 (100.5,148.2)	0.3923	.
Quartile 3	78.1 (54.7,101.4)	0.1985	.	77.3 (47.0,107.5)	0.3946	.	90.3 (67.0,113.7)	0.8077	.	68.4 (45.8,90.9)	0.0043	.
Quartile 4	13.9 (-15.2,43.0)	0.0204	.	81.3 (47.9,114.7)	0.3183	.	80.8 (59.4,102.2)	0.7353	.	99.7 (78.4,120.9)	0.4266	.
Fish												
Quartile 1	58.3 (31.7,84.9)	.	0.1940	105.3 (80.4,130.2)	.	0.0002	74.0 (47.6,100.5)	.	0.1944	94.7 (72.2,117.2)	.	0.7559
Quartile 2	50.4 (31.9,68.8)	0.6292	.	84.8 (61.4,108.2)	0.2379	.	68.7 (50.1,87.3)	0.7471	.	102.4 (85.1,119.7)	0.5954	.
Quartile 3	32.1 (8.8,55.5)	0.1459	.	46.7 (23.6,69.8)	0.0008	.	95.7 (74.5,117.0)	0.2096	.	109.7 (88.8,130.6)	0.3379	.
Quartile 4	70.8 (42.5,99.1)	0.5269	.	37.4 (13.8,61.0)	0.0001	.	92.2 (69.6,114.8)	0.3046	.	94.2 (64.2,124.1)	0.9766	.
MUFA: SFA												
Quartile 1	29.6 (10.6,48.5)	.	0.0171	59.9 (30.0,89.9)	.	0.7727	85.6 (64.2,107.1)	.	0.9434	112.7 (90.7,134.8)	.	0.0062
Quartile 2	55.6 (25.3,86.0)	0.1523	.	70.7 (49.3,92.1)	0.5651	.	79.3 (58.7,99.9)	0.6775	.	97.6 (77.5,117.6)	0.3180	.
Quartile 3	83.0 (55.5,110.5)	0.0018	.	78.1 (50.6,105.6)	0.3791	.	77.7 (54.5,100.8)	0.6206	.	125.8 (103.5,148.0)	0.4127	.
Quartile 4	54.1 (33.7,74.6)	0.0828	.	62.0 (37.9,86.0)	0.9177	.	85.1 (63.0,107.2)	0.9726	.	73.7 (52.7,94.8)	0.0122	.
Alcohol												
In interval	47.3 (-8.9,103.5)	0.9136	.	113.7 (64.0,163.4)	0.0655	.	49.9 (21.2,78.6)	0.0007	.	69.8 (32.9,106.7)	0.0002	.

Not in interval	50.5 (38.5,62.4)	.	0.9136	65.5 (52.6,78.3)	.	0.0655	87.3 (75.6,99.0)	<.0001	0.0179	104.2 (93.0,115.3)	<.0001	0.0810

Abbreviations: aMedi=Alternative Mediterranean Diet Index; AREDS=Age-Related Eye Diseases Study; CI=confidence interval; MUFA: SFA=monounsaturated fatty acid: saturated fatty acid

* For red meat, higher quartiles refer to lower levels of intake, which is more adherent to the Alternative Mediterranean Diet Index

Table S9. Geographic Atrophy Proximity-Based Progression Rates, according to Quantiles of Nutrient Intake, separately according to Randomized Oral Supplement Assignments, in the Age-Related Eye Diseases Studies 1 and 2.

	AREDS						AREDS2					
	Antioxidants			No Antioxidants			Lutein/Zeaxanthin			No Lutein/Zeaxanthin		
Nutrient	Estimate, µm/year (CI)	Pairwise P Value	Interaction P Value	Estimate, µm/year (CI)	Pairwise P Value	Interaction P Value	Estimate, µm/year (CI)	Pairwise P Value	Interaction P Value	Estimate, µm/year (CI)	Pairwise P Value	Interaction P Value
Vitamin A, IU												
Tertile 1	49.6 (30.5,68.6)	.	0.3450	71.1 (53.9,88.3)	.	0.0039	85.5 (67.6,103.5)	.	0.1259	95.5 (76.8,114.3)	.	0.5962
Tertile 2	61.2 (40.5,81.9)	0.4180	.	101.8 (74.8,128.8)	0.0597	.	92.6 (74.2,111.0)	0.5909	.	108.7 (90.5,127.0)	0.3216	.
Tertile 3	39.3 (18.3,60.3)	0.4747	.	41.8 (19.1,64.4)	0.0430	.	65.1 (44.9,85.2)	0.1367	.	99.7 (81.0,118.4)	0.7597	.
Vitamin A, RAE												
Tertile 1	.	.	.	.	.	.	76.3 (58.0,94.6)	.	0.3566	93.3 (76.1,110.5)	.	0.1517
Tertile 2	.	.	.	.	.	.	93.1 (74.5,111.7)	0.2079	.	118.4 (98.1,138.8)	0.0648	.
Tertile 3	.	.	.	.	.	.	76.3 (56.6,95.9)	0.9962	.	96.9 (78.5,115.3)	0.7825	.
Retinol, mcg												
Tertile 1	53.1 (33.6,72.6)	.	0.0819	37.3 (16.8,57.8)	.	0.0010	77.6 (56.6,98.6)	.	0.8726	98.9 (78.9,118.8)	.	0.7161
Tertile 2	38.1 (21.0,55.2)	0.2548	.	88.6 (66.4,110.7)	0.0009	.	83.0 (66.6,99.3)	0.6926	.	98.1 (81.0,115.1)	0.9527	.
Tertile 3	73.9 (47.2,100.7)	0.2165	.	83.0 (62.4,103.7)	0.0022	.	85.1 (64.9,105.4)	0.6135	.	108.0 (89.0,127.0)	0.5171	.
Vitamin D, mcg												
Tertile 1	47.7 (27.2,68.3)	.	0.1010	75.8 (55.2,96.4)	.	0.0216	87.0 (66.5,107.6)	.	0.8582	106.2 (89.2,123.3)	.	0.7594
Tertile 2	35.3 (14.9,55.7)	0.3968	.	45.9 (25.1,66.6)	0.0447	.	80.4 (62.1,98.7)	0.6376	.	97.0 (79.5,114.4)	0.4588	.
Tertile 3	65.6 (46.4,84.9)	0.2118	.	87.4 (64.7,110.1)	0.4548	.	80.0 (62.1,97.9)	0.6139	.	101.2 (78.7,123.7)	0.7292	.
Vitamin E, mg												
Tertile 1	38.5 (15.9,61.0)	.	0.0402	71.5 (49.2,93.9)	.	0.9559	69.1 (51.2,87.0)	.	0.0018	122.2 (104.7,139.6)	.	0.0124
Tertile 2	41.4 (23.3,59.5)	0.8411	.	68.2 (48.1,88.3)	0.8255	.	110.8 (91.8,129.8)	0.0018	.	92.7 (72.1,113.3)	0.0325	.
Tertile 3	72.0 (51.9,92.1)	0.0297	.	66.8 (43.7,89.9)	0.7729	.	68.8 (49.5,88.0)	0.9786	.	86.6 (68.9,104.4)	0.0052	.
Vitamin C, mg												
Tertile 1	61.5 (38.4,84.5)	.	0.3765	88.8 (69.4,108.3)	.	0.0009	83.4 (62.0,104.8)	.	0.9859	88.3 (71.0,105.5)	.	0.1576
Tertile 2	41.0 (22.7,59.3)	0.1709	.	36.5 (15.6,57.3)	0.0004	.	82.0 (64.8,99.2)	0.9178	.	110.8 (93.5,128.0)	0.0705	.
Tertile 3	52.4 (32.2,72.5)	0.5575	.	79.6 (56.9,102.3)	0.5415	.	81.0 (62.3,99.6)	0.8664	.	108.0 (85.5,130.5)	0.1718	.
Thiamine, mg												
Tertile 1	47.7 (26.0,69.3)	.	0.9304	60.7 (42.9,78.6)	.	0.0455	91.0 (69.4,112.6)	.	0.6312	109.7 (91.2,128.2)	.	0.1705
Tertile 2	52.7 (34.3,71.1)	0.7290	.	99.9 (72.9,126.9)	0.0180	.	77.5 (58.9,96.2)	0.3538	.	106.1 (88.8,123.4)	0.7779	.
Tertile 3	48.6 (27.2,70.0)	0.9532	.	62.2 (40.0,84.4)	0.9178	.	80.5 (63.5,97.5)	0.4545	.	85.3 (65.3,105.3)	0.0792	.
Riboflavin, mg												
Tertile 1	40.1 (21.5,58.7)	.	0.1247	75.1 (54.5,95.7)	.	0.6529	89.9 (69.3,110.6)	.	0.6851	103.4 (85.4,121.4)	.	0.0973
Tertile 2	46.6 (26.8,66.3)	0.6377	.	62.1 (42.7,81.5)	0.3678	.	79.1 (60.2,98.1)	0.4499	.	113.3 (95.5,131.1)	0.4401	.
Tertile 3	69.7 (47.2,92.2)	0.0465	.	71.4 (43.8,98.9)	0.8307	.	79.2 (61.9,96.5)	0.4344	.	84.2 (64.4,104.0)	0.1604	.
Niacin, mg												
Tertile 1	54.3 (36.6,71.9)	.	0.3249	66.8 (47.0,86.6)	.	0.2963	84.9 (64.5,105.2)	.	0.8825	92.9 (75.1,110.6)	.	0.0788
Tertile 2	34.1 (10.1,58.2)	0.1848	.	61.7 (42.2,81.1)	0.7157	.	83.3 (65.1,101.5)	0.9101	.	118.2 (100.0,136.4)	0.0509	.
Tertile 3	56.0 (35.8,76.2)	0.8977	.	88.8 (60.1,117.4)	0.2160	.	78.4 (60.2,96.5)	0.6402	.	91.7 (71.9,111.5)	0.9308	.
Vitamin B6, mg												
Tertile 1	36.5 (17.1,55.9)	.	0.1970	61.1 (41.8,80.5)	.	0.0395	88.8 (69.2,108.4)	.	0.0302	97.4 (80.1,114.6)	.	0.7734
Tertile 2	60.9 (39.9,81.9)	0.0937	.	59.4 (39.3,79.4)	0.9000	.	100.4 (79.7,121.1)	0.4262	.	106.3 (88.7,124.0)	0.4762	.
Tertile 3	56.1 (35.8,76.3)	0.1702	.	99.3 (72.6,126.0)	0.0232	.	66.1 (49.4,82.8)	0.0828	.	100.8 (79.2,122.3)	0.8082	.
Folate, mcg												
Tertile 1	54.6 (33.8,75.3)	.	0.8092	78.2 (59.0,97.5)	.	0.0161	92.8 (71.5,114.1)	.	0.4761	103.1 (85.8,120.4)	.	0.9516
Tertile 2	50.7 (31.9,69.5)	0.7855	.	48.7 (30.1,67.2)	0.0300	.	75.9 (58.6,93.2)	0.2268	.	99.2 (81.1,117.2)	0.7572	.
Tertile 3	44.7 (22.9,66.5)	0.5180	.	94.3 (64.5,124.1)	0.3725	.	80.9 (62.4,99.4)	0.4061	.	102.0 (81.1,122.9)	0.9350	.
Natural Food Folate												
Tertile 1	.	.	.	.	.	.	80.5 (58.9,102.1)	.	0.9517	95.1 (77.8,112.3)	.	0.2410

Tertile 2	.	.	.	.	.	.	84.4 (66.3,102.5)	0.7870	.	114.3 (95.8,132.7)	0.1360	.
Tertile 3	.	.	.	.	.	.	80.9 (63.5,98.4)	0.9748	.	94.5 (74.2,114.7)	0.9653	.
Folic Acid												
Tertile 1	.	.	.	.	.	.	100.5 (80.5,120.6)	.	0.0892	96.6 (77.3,115.8)	.	0.8351
Tertile 2	.	.	.	.	.	.	70.7 (52.2,89.1)	0.0319	.	102.7 (83.5,122.0)	0.6559	.
Tertile 3	.	.	.	.	.	.	78.9 (61.0,96.8)	0.1145	.	104.2 (86.8,121.5)	0.5643	.
Vitamin B12, mcg												
Tertile 1	34.2 (17.1,51.4)	.	0.0413	78.2 (52.5,103.9)	.	0.7117	86.0 (66.5,105.5)	.	0.5021	119.0 (100.2,137.9)	.	0.0442
Tertile 2	61.4 (40.4,82.4)	0.0496	.	66.1 (47.7,84.5)	0.4497	.	72.9 (54.2,91.7)	0.3417	.	86.7 (69.7,103.7)	0.0126	.
Tertile 3	67.1 (43.5,90.6)	0.0273	.	65.6 (42.6,88.5)	0.4714	.	87.2 (68.9,105.4)	0.9344	.	102.6 (82.7,122.6)	0.2406	.
Beta-carotene, mcg												
Tertile 1	60.0 (40.7,79.3)	.	0.2292	75.2 (56.5,93.8)	.	0.1503	83.1 (65.3,101.0)	.	0.1918	100.4 (82.7,118.0)	.	0.4673
Tertile 2	51.6 (32.3,70.9)	0.5427	.	78.1 (54.9,101.3)	0.8457	.	93.5 (74.5,112.5)	0.4344	.	93.6 (74.7,112.5)	0.6079	.
Tertile 3	34.5 (12.1,56.8)	0.0887	.	48.6 (24.5,72.7)	0.0860	.	68.3 (48.5,88.0)	0.2719	.	110.5 (91.3,129.7)	0.4478	.
Beta-carotene equivalents, mcg												
Tertile 1	63.7 (42.3,85.1)	.	0.1792	75.6 (57.0,94.2)	.	0.1530	.	.	.	.	.	.
Tertile 2	50.5 (32.8,68.1)	0.3495	.	77.2 (54.0,100.5)	0.9159	.	.	.	.	.	.	.
Tertile 3	34.5 (12.1,56.8)	0.0641	.	48.6 (24.5,72.6)	0.0802	.	.	.	.	.	.	.
Alpha-carotene, mcg												
Tertile 1	72.1 (48.9,95.2)	.	0.1062	74.0 (52.8,95.3)	.	0.6380	82.9 (63.4,102.5)	.	0.1780	101.5 (82.9,120.0)	.	0.7378
Tertile 2	42.4 (25.1,59.7)	0.0443	.	71.6 (50.4,92.9)	0.8746	.	92.1 (74.9,109.2)	0.4906	.	106.1 (88.5,123.6)	0.7235	.
Tertile 3	44.4 (23.0,65.8)	0.0850	.	59.8 (36.9,82.7)	0.3703	.	67.0 (46.9,87.2)	0.2668	.	95.5 (75.7,115.4)	0.6690	.
Beta-cryptoxanthin, mcg												
Tertile 1	63.9 (43.1,84.7)	.	0.0255	81.3 (62.0,100.5)	.	0.0335	104.9 (82.0,127.8)	.	0.0638	105.0 (88.5,121.5)	.	0.2495
Tertile 2	60.8 (40.7,80.9)	0.8332	.	43.6 (21.4,65.9)	0.0125	.	70.8 (53.8,87.9)	0.0192	.	88.8 (70.2,107.5)	0.2024	.
Tertile 3	29.0 (9.8,48.2)	0.0160	.	75.1 (52.6,97.7)	0.6846	.	81.2 (63.5,99.0)	0.1087	.	111.4 (90.1,132.7)	0.6436	.
Lutein and zeaxanthin, mcg												
Tertile 1	45.0 (27.4,62.5)	.	0.3639	74.1 (57.3,90.8)	.	0.6572	67.0 (48.1,86.0)	.	0.1628	96.9 (80.2,113.5)	.	0.1182
Tertile 2	62.6 (42.1,83.1)	0.1997	.	58.1 (27.3,88.8)	0.3678	.	90.3 (71.9,108.6)	0.0842	.	90.1 (69.7,110.5)	0.6154	.
Tertile 3	43.8 (19.5,68.0)	0.9392	.	68.0 (43.9,92.2)	0.6850	.	88.6 (69.5,107.7)	0.1166	.	117.6 (98.7,136.5)	0.1069	.
Lycopene, mcg												
Tertile 1	59.0 (39.0,79.0)	.	0.5464	93.9 (73.4,114.4)	.	0.0079	64.4 (43.4,85.4)	.	0.1224	99.3 (79.8,118.9)	.	0.3520
Tertile 2	47.4 (28.4,66.3)	0.4058	.	48.9 (29.0,68.9)	0.0021	.	93.1 (75.4,110.7)	0.0405	.	111.1 (93.4,128.7)	0.3804	.
Tertile 3	43.4 (21.3,65.5)	0.3016	.	63.5 (39.2,87.7)	0.0593	.	81.8 (63.6,100.0)	0.2199	.	92.5 (74.0,111.0)	0.6205	.
Calcium, mg												
Tertile 1	36.1 (16.8,55.5)	.	0.1182	67.8 (47.5,88.0)	.	0.8819	77.1 (56.6,97.6)	.	0.6021	116.6 (99.5,133.7)	.	0.0720
Tertile 2	51.2 (31.9,70.5)	0.2780	.	73.1 (51.1,95.0)	0.7257	.	89.7 (71.4,108.1)	0.3665	.	88.5 (70.0,107.1)	0.0292	.
Tertile 3	66.9 (44.9,88.9)	0.0394	.	65.1 (41.4,88.8)	0.8668	.	79.0 (61.1,96.8)	0.8903	.	95.2 (75.0,115.4)	0.1116	.
Magnesium, mg												
Tertile 1	35.6 (17.8,53.4)	.	0.0225	64.9 (45.1,84.6)	.	0.7718	94.4 (72.6,116.1)	.	0.3337	109.3 (91.1,127.5)	.	0.1796
Tertile 2	70.3 (51.9,88.7)	0.0082	.	66.3 (43.5,89.1)	0.9269	.	73.8 (57.0,90.7)	0.1427	.	105.9 (88.8,123.0)	0.7893	.
Tertile 3	40.8 (14.1,67.5)	0.7494	.	75.4 (52.5,98.2)	0.4939	.	84.0 (65.3,102.8)	0.4802	.	84.9 (64.1,105.6)	0.0825	.
Iron, mg												
Tertile 1	40.9 (23.3,58.4)	.	0.0081	98.7 (79.7,117.7)	.	<.0001	86.6 (65.3,107.9)	.	0.8933	104.3 (86.8,121.8)	.	0.7851
Tertile 2	88.8 (62.5,115.1)	0.0032	.	34.4 (16.9,52.0)	<.0001	.	80.9 (62.5,99.3)	0.6913	.	103.2 (84.9,121.6)	0.9351	.
Tertile 3	43.9 (25.3,62.4)	0.8165	.	95.1 (65.5,124.6)	0.8398	.	80.4 (62.9,97.8)	0.6573	.	95.5 (75.5,115.4)	0.5143	.
Zinc, mg												
Tertile 1	57.4 (38.9,75.8)	.	0.1658	54.0 (35.3,72.6)	.	0.0053	92.6 (72.5,112.7)	.	0.0566	94.7 (75.9,113.5)	.	0.0180
Tertile 2	34.1 (13.7,54.6)	0.0973	.	100.7 (77.8,123.6)	0.0020	.	90.8 (72.8,108.7)	0.8917	.	120.7 (103.2,138.2)	0.0472	.
Tertile 3	58.9 (37.0,80.9)	0.9142	.	58.9 (36.0,81.8)	0.7384	.	63.8 (45.4,82.2)	0.0381	.	84.7 (65.4,104.0)	0.4654	.
Copper, mg												

Tertile 1	33.8 (15.5,52.1)	.	0.0557	70.8 (52.1,89.6)	.	0.3383	69.7 (50.4,89.0)	.	0.0809	90.7 (71.5,109.9)	.	0.0050
Tertile 2	65.0 (47.2,82.8)	0.0170	.	79.9 (54.7,105.2)	0.5695	.	98.3 (80.2,116.4)	0.0340	.	124.5 (106.9,142.1)	0.0110	.
Tertile 3	53.6 (26.1,81.1)	0.2391	.	55.5 (33.4,77.7)	0.2999	.	76.3 (57.5,95.2)	0.6297	.	85.4 (66.8,104.1)	0.6977	.
Selenium, mcg												
Tertile 1	42.3 (23.2,61.5)	.	0.5892	95.0 (73.5,116.6)	.	0.0145	82.8 (67.7,97.9)	.	0.8873	95.1 (80.7,109.4)	.	0.4224
Tertile 2	54.6 (33.8,75.5)	0.3909	.	56.8 (39.8,73.8)	0.0065	.	89.7 (52.0,127.4)	0.7385	.	111.7 (49.5,174.0)	0.6085	.
Tertile 3	55.2 (34.3,76.2)	0.3705	.	53.5 (21.8,85.2)	0.0337	.	79.7 (62.5,96.9)	0.7913	.	109.3 (92.7,125.9)	0.2038	.
Saturated fat, % kcal												
Tertile 1	38.6 (19.0,58.2)	.	0.0137	59.8 (38.5,81.0)	.	0.0995	81.5 (63.7,99.4)	.	0.1105	97.3 (76.6,117.9)	.	0.6233
Tertile 2	77.4 (56.4,98.3)	0.0083	.	57.8 (35.8,79.8)	0.8997	.	69.9 (51.6,88.3)	0.3736	.	108.9 (90.2,127.6)	0.4120	.
Tertile 3	40.9 (21.6,60.2)	0.8698	.	87.4 (66.2,108.5)	0.0709	.	99.3 (78.8,119.8)	0.1990	.	97.9 (81.1,114.7)	0.9637	.
Monounsaturated fat, % kcal												
Tertile 1	55.6 (30.9,80.2)	.	0.2579	46.8 (25.1,68.4)	.	0.0556	81.4 (64.5,98.2)	.	0.6808	111.9 (91.0,132.8)	.	0.1543
Tertile 2	38.2 (19.5,56.9)	0.2699	.	82.0 (57.3,106.8)	0.0359	.	76.9 (57.8,96.0)	0.7295	.	109.1 (90.2,128.0)	0.8464	.
Tertile 3	59.4 (40.7,78.1)	0.8059	.	76.8 (57.9,95.7)	0.0398	.	89.6 (68.2,110.9)	0.5532	.	89.1 (72.6,105.6)	0.0938	.
Cholesterol, mg												
Tertile 1	45.9 (26.6,65.1)	.	0.6788	106.4 (83.5,129.3)	.	0.0001	88.6 (69.2,107.9)	.	0.0170	94.9 (76.7,113.1)	.	0.5962
Tertile 2	58.2 (36.6,79.8)	0.3999	.	40.7 (20.9,60.4)	<.0001	.	63.1 (45.8,80.5)	0.0552	.	109.2 (88.3,130.2)	0.3107	.
Tertile 3	48.1 (28.0,68.3)	0.8731	.	69.9 (49.4,90.3)	0.0198	.	99.9 (80.2,119.6)	0.4201	.	101.9 (84.8,119.0)	0.5803	.
Oleic acid, mg per 1000 kcal												
Tertile 1	54.4 (29.6,79.2)	.	0.1956	46.5 (25.1,68.0)	.	0.0093	83.5 (66.9,100.2)	.	0.4946	112.7 (91.5,134.0)	.	0.0254
Tertile 2	37.2 (18.4,55.9)	0.2756	.	96.1 (72.8,119.5)	0.0023	.	72.9 (53.1,92.7)	0.4204	.	114.3 (96.2,132.5)	0.9107	.
Tertile 3	60.9 (42.4,79.5)	0.6795	.	66.9 (47.6,86.3)	0.1638	.	90.0 (69.1,110.8)	0.6352	.	83.6 (66.8,100.4)	0.0353	.
Linoleic acid, mg per 1000 kcal												
Tertile 1	71.1 (47.1,95.1)	.	0.0778	42.3 (18.9,65.8)	.	<.0001	81.3 (62.1,100.4)	.	0.9192	101.2 (79.8,122.6)	.	0.2890
Tertile 2	35.9 (16.9,54.9)	0.0245	.	128.8 (105.0,152.6)	<.0001	.	85.1 (66.1,104.1)	0.7789	.	112.2 (94.1,130.4)	0.4406	.
Tertile 3	51.8 (33.4,70.2)	0.2111	.	53.5 (37.0,70.1)	0.4434	.	79.7 (61.5,98.0)	0.9100	.	92.3 (75.5,109.2)	0.5231	.
a-Linolenic acid, mg per 1000 kcal												
Tertile 1	63.8 (45.5,82.1)	.	0.1446	69.5 (47.6,91.3)	.	0.8178	85.3 (66.8,103.8)	.	0.1512	119.9 (100.1,139.7)	.	0.0001
Tertile 2	41.8 (13.7,70.0)	0.1977	.	62.9 (39.7,86.1)	0.6855	.	97.2 (75.8,118.6)	0.4074	.	121.1 (102.0,140.2)	0.9317	.
Tertile 3	39.4 (21.5,57.3)	0.0619	.	72.8 (52.3,93.4)	0.8255	.	70.5 (53.3,87.7)	0.2508	.	73.1 (56.4,89.9)	0.0004	.
EPA, mg per 1000 kcal												
Tertile 1	56.0 (35.2,76.8)	.	0.8068	72.3 (55.3,89.4)	.	0.4946	69.5 (50.4,88.5)	.	0.1451	94.9 (77.9,112.0)	.	0.0128
Tertile 2	47.7 (29.3,66.1)	0.5580	.	72.1 (47.4,96.7)	0.9857	.	97.0 (77.1,116.9)	0.0500	.	120.9 (103.6,138.2)	0.0363	.
Tertile 3	47.4 (25.1,69.7)	0.5813	.	53.6 (26.1,81.2)	0.2560	.	81.1 (63.4,98.8)	0.3814	.	80.6 (58.2,103.0)	0.3192	.
DHA, mg per 1000 kcal												
Tertile 1	46.3 (26.0,66.5)	.	0.1786	102.7 (81.9,123.5)	.	0.0007	60.8 (40.9,80.7)	.	0.0079	97.9 (80.9,115.0)	.	0.0170
Tertile 2	63.4 (44.6,82.2)	0.2221	.	51.5 (33.3,69.7)	0.0003	.	102.5 (84.8,120.3)	0.0022	.	118.5 (101.3,135.7)	0.0959	.
Tertile 3	37.2 (15.5,58.9)	0.5471	.	54.0 (28.4,79.7)	0.0041	.	77.7 (58.9,96.5)	0.2263	.	77.8 (55.2,100.4)	0.1628	.
DPA, mg per 1000 kcal												
Tertile 1	.	.	.	.	.	.	87.4 (66.0,108.7)	.	0.7315	90.1 (73.3,106.8)	.	0.0100
Tertile 2	.	.	.	.	.	.	84.1 (65.8,102.4)	0.8183	.	124.6 (106.3,142.9)	0.0065	.
Tertile 3	.	.	.	.	.	.	76.9 (59.4,94.4)	0.4551	.	89.1 (68.2,109.9)	0.9427	.
EPA+DHA, mg per 1000 kcal												
Tertile 1	50.7 (31.5,69.9)	.	0.4263	91.8 (73.5,110.0)	.	0.0040	67.0 (47.7,86.4)	.	0.0476	91.1 (73.8,108.4)	.	0.0142
Tertile 2	58.2 (38.7,77.7)	0.5880	.	47.4 (26.8,67.9)	0.0016	.	100.7 (81.5,119.8)	0.0153	.	121.0 (104.0,138.0)	0.0159	.

Tertile 3	38.6 (16.2,60.9)	0.4182	.	55.4 (28.7,82.1)	0.0274	.	78.8 (60.8,96.8)	0.3815	.	84.9 (62.4,107.4)	0.6661	.
EPA+DPA+DHA, mg per 1000 kcal												
Tertile 1	.	.	.	.	.	.	67.7 (49.1,86.3)	.	0.1682	88.3 (71.6,105.0)	.	0.0004
Tertile 2	.	.	.	.	.	.	91.8 (73.2,110.4)	0.0723	.	126.5 (109.9,143.0)	0.0015	.
Tertile 3	.	.	.	.	.	.	86.6 (67.4,105.9)	0.1649	.	73.5 (48.5,98.5)	0.3320	.
Arachidonic acid, mg per 1000 kcal												
Tertile 1	64.1 (41.2,87.0)	.	0.3090	84.5 (62.3,106.7)	.	0.2329	87.9 (68.4,107.4)	.	0.5468	98.1 (80.2,115.9)	.	0.0002
Tertile 2	40.2 (19.6,60.8)	0.1274	.	63.5 (43.3,83.8)	0.1701	.	74.3 (56.4,92.2)	0.3133	.	76.6 (58.1,95.0)	0.1013	.
Tertile 3	49.2 (31.2,67.3)	0.3152	.	59.1 (36.5,81.6)	0.1141	.	85.7 (66.5,104.8)	0.8723	.	131.6 (112.6,150.6)	0.0117	.
Galactose, gm												
Tertile 1	35.2 (15.6,54.9)	.	0.1002	62.4 (44.7,80.2)	.	0.6312	.	.	.	.	.	.
Tertile 2	66.1 (45.9,86.4)	0.0322	.	74.1 (49.2,98.9)	0.4528	.	.	.	.	.	.	.
Tertile 3	49.8 (29.5,70.0)	0.3108	.	75.0 (50.5,99.4)	0.4133	.	.	.	.	.	.	.
Lactose, gm												
Tertile 1	39.5 (21.1,57.8)	.	0.0996	74.7 (54.6,94.8)	.	0.1011	82.2 (61.6,102.8)	.	0.8517	103.9 (85.5,122.4)	.	0.5320
Tertile 2	43.7 (21.3,66.1)	0.7733	.	84.5 (60.1,108.8)	0.5426	.	85.4 (67.7,103.0)	0.8175	.	107.2 (88.7,125.8)	0.8042	.
Tertile 3	67.8 (47.8,87.8)	0.0409	.	51.2 (30.0,72.3)	0.1137	.	78.0 (59.4,96.6)	0.7660	.	92.9 (74.2,111.5)	0.4075	.
Alcohol, gm												
Tertile 1	43.0 (27.0,59.0)	.	0.0055	59.7 (40.6,78.8)	.	0.1080	82.2 (62.9,101.5)	.	0.0280	127.6 (108.9,146.3)	.	0.0023
Tertile 2	85.5 (61.2,109.8)	0.0044	.	89.1 (66.5,111.7)	0.0510	.	97.7 (80.2,115.2)	0.2418	.	82.8 (65.6,100.1)	0.0006	.
Tertile 3	35.2 (12.6,57.8)	0.5776	.	60.6 (36.7,84.4)	0.9560	.	61.7 (41.9,81.5)	0.1466	.	97.4 (78.0,116.8)	0.0282	.
Soluble Dietary Fiber, gm												
Tertile 1	57.3 (39.4,75.3)	.	0.5909	59.7 (37.5,82.0)	.	0.1033	.	.	.	.	.	.
Tertile 2	44.7 (26.5,62.8)	0.3275	.	62.7 (44.4,80.9)	0.8413	.	.	.	.	.	.	.
Tertile 3	46.1 (16.7,75.6)	0.5222	.	93.9 (67.4,120.4)	0.0525	.	.	.	.	.	.	.
Insoluble Dietary Fiber, gm												
Tertile 1	59.1 (41.1,77.0)	.	0.4250	59.8 (40.7,78.9)	.	0.0018	.	.	.	.	.	.
Tertile 2	42.3 (24.5,60.2)	0.1939	.	103.6 (80.9,126.3)	0.0038	.	.	.	.	.	.	.
Tertile 3	48.3 (18.2,78.4)	0.5468	.	49.3 (27.1,71.5)	0.4801	.	.	.	.	.	.	.
Fiber (Soluble or Insoluble), gm												
Tertile 1	.	.	.	.	.	.	69.4 (49.7,89.0)	.	0.0437	102.9 (85.5,120.3)	.	0.6578
Tertile 2	.	.	.	.	.	.	100.3 (82.3,118.2)	0.0228	.	106.5 (87.4,125.5)	0.7854	.
Tertile 3	.	.	.	.	.	.	74.0 (55.2,92.8)	0.7387	.	94.2 (74.8,113.5)	0.5116	.
Total Choline												
Tertile 1	.	.	.	.	.	.	73.3 (51.9,94.8)	.	0.4363	96.9 (80.0,113.7)	.	0.4546
Tertile 2	.	.	.	.	.	.	90.5 (73.3,107.6)	0.2216	.	111.8 (92.4,131.2)	0.2552	.
Tertile 3	.	.	.	.	.	.	79.1 (60.5,97.6)	0.6920	.	96.9 (77.2,116.7)	0.9973	.
Free Choline												
Tertile 1	.	.	.	.	.	.	90.6 (70.7,110.6)	.	0.4544	114.8 (97.0,132.6)	.	0.1528
Tertile 2	.	.	.	.	.	.	83.2 (64.7,101.7)	0.5930	.	97.5 (80.0,115.0)	0.1745	.
Tertile 3	.	.	.	.	.	.	73.6 (55.5,91.6)	0.2134	.	88.9 (68.2,109.6)	0.0634	.
Glycemic Index												
Tertile 1	.	.	.	.	.	.	77.7 (60.2,95.2)	.	0.6008	105.8 (86.4,125.2)	.	0.8246
Tertile 2	.	.	.	.	.	.	90.3 (70.9,109.8)	0.3446	.	97.6 (80.3,114.8)	0.5354	.
Tertile 3	.	.	.	.	.	.	79.1 (59.4,98.8)	0.9174	.	101.6 (82.4,120.9)	0.7657	.
Glycemic Load												
Tertile 1	.	.	.	.	.	.	76.4 (57.0,95.7)	.	0.7343	88.8 (72.1,105.4)	.	0.0891
Tertile 2	.	.	.	.	.	.	82.3 (62.8,101.9)	0.6699	.	102.5 (82.1,122.9)	0.3070	.
Tertile 3	.	.	.	.	.	.	86.9 (69.1,104.6)	0.4322	.	117.1 (98.0,136.3)	0.0282	.

Abbreviations: AREDS=Age-Related Eye Diseases Study; CI=confidence interval; DHA=docosahexaenoic acid; DPA=docosapentaenoic acid; EPA=eicosapentaenoic acid; IU=international units; RAE=retinol activity equivalents

Table S10. Rates of Decline in Best-Corrected Visual Acuity, according to Quantiles of the Alternative Mediterranean Dietary Index and its Components, in the Age-Related Eye Diseases Studies 1 and 2.

	AREDS (n = 657 eyes of 508 participants)			AREDS2 (n = 1179 eyes of 867 participants)		
	Estimate, ETDRS letters/year (CI)	Pairwise P Value	Interaction P Value	Estimate, ETDRS letters/year (CI)	Pairwise P Value	Interaction P Value
aMedi						
Tertile 1	2.9 (2.6,3.2)	.	0.9951	2.3 (2.0,2.7)	.	0.4881
Tertile 2	2.9 (2.5,3.2)	0.9215	.	2.1 (1.7,2.4)	0.2897	.
Tertile 3	2.9 (2.4,3.3)	0.9627	.	2.1 (1.7,2.4)	0.3270	.
Whole fruit						
Quartile 1	3.1 (2.7,3.5)	.	0.6672	2.5 (2.1,2.9)	.	0.1502
Quartile 2	2.7 (2.3,3.2)	0.2453	.	2.3 (1.9,2.7)	0.5363	.
Quartile 3	2.8 (2.4,3.2)	0.3445	.	2.1 (1.7,2.5)	0.1661	.
Quartile 4	2.9 (2.4,3.4)	0.5862	.	1.8 (1.4,2.2)	0.0305	.
Vegetables						
Quartile 1	2.8 (2.4,3.2)	.	0.8164	2.2 (1.8,2.6)	.	0.5752
Quartile 2	2.8 (2.4,3.2)	0.8756	.	2.4 (2.0,2.8)	0.4192	.
Quartile 3	2.9 (2.4,3.3)	0.9020	.	2.1 (1.7,2.6)	0.9562	.
Quartile 4	3.1 (2.6,3.5)	0.4272	.	2.0 (1.6,2.4)	0.5584	.
Whole grains						
Quartile 1	3.3 (2.9,3.7)	.	0.1022	2.8 (2.3,3.2)	.	0.0232
Quartile 2	2.6 (2.1,3.0)	0.0170	.	1.9 (1.5,2.3)	0.0037	.
Quartile 3	2.8 (2.4,3.2)	0.1259	.	2.0 (1.6,2.4)	0.0129	.
Quartile 4	2.8 (2.3,3.2)	0.0967	.	2.2 (1.7,2.6)	0.0508	.
Nuts						
Quartile 1	2.8 (2.4,3.2)	.	0.8210	2.2 (1.8,2.6)	.	0.5626
Quartile 2	2.8 (2.4,3.3)	0.9626	.	1.9 (1.5,2.3)	0.3246	.
Quartile 3	2.8 (2.4,3.2)	0.8658	.	2.3 (1.9,2.7)	0.7485	.
Quartile 4	3.1 (2.6,3.5)	0.4750	.	2.3 (1.9,2.7)	0.8123	.
Legumes						
Quartile 1	2.9 (2.4,3.3)	.	0.8796	2.0 (1.5,2.5)	.	0.0036
Quartile 2	2.8 (2.4,3.2)	0.8711	.	1.6 (1.2,2.0)	0.1639	.
Quartile 3	2.8 (2.4,3.2)	0.8522	.	2.4 (2.0,2.7)	0.2259	.
Quartile 4	3.0 (2.6,3.5)	0.5888	.	2.6 (2.2,3.0)	0.0875	.
Red meat*						
Quartile 1	2.8 (2.5,3.2)	.	0.0768	3.0 (2.6,3.4)	.	<.0001
Quartile 2	3.3 (2.9,3.8)	0.0857	.	2.0 (1.6,2.4)	0.0002	.
Quartile 3	2.6 (2.2,3.0)	0.4392	.	1.7 (1.3,2.1)	<.0001	.
Quartile 4	2.7 (2.2,3.1)	0.5763	.	1.9 (1.4,2.3)	<.0001	.
Fish						
Quartile 1	2.8 (2.4,3.3)	.	0.0605	2.1 (1.6,2.6)	.	0.1585
Quartile 2	3.3 (2.9,3.7)	0.1164	.	2.3 (1.9,2.6)	0.5223	.
Quartile 3	2.7 (2.2,3.1)	0.6340	.	2.4 (2.0,2.8)	0.3184	.
Quartile 4	2.5 (2.1,3.0)	0.3667	.	1.7 (1.2,2.2)	0.3216	.
MUFA: SFA						
Quartile 1	3.0 (2.6,3.5)	.	0.3781	2.8 (2.3,3.2)	.	0.0107
Quartile 2	3.0 (2.5,3.4)	0.7963	.	1.9 (1.5,2.2)	0.0014	.
Quartile 3	3.0 (2.6,3.4)	0.8300	.	2.2 (1.8,2.6)	0.0755	.
Quartile 4	2.6 (2.2,3.0)	0.1217	.	2.0 (1.5,2.4)	0.0117	.
Alcohol						
In interval	2.8 (2.6,3.0)	.	0.1096	2.8 (2.2,3.4)	.	.

Not in interval	3.4 (2.7,4.1)	0.1096	.	2.1 (1.9,2.3)	0.0233	0.0233

Abbreviations: aMedi=Alternative Mediterranean Diet Index; AREDS=Age-Related Eye Diseases Study; CI=confidence interval; ETDRS=Early Treatment Diabetic Retinopathy Study; MUFA: SFA=monounsaturated fatty acid: saturated fatty acid

* For red meat, higher quartiles refer to lower levels of intake, which is more adherent to the Alternative Mediterranean Diet Index

Table S11. Rates of Decline in Best-Corrected Visual Acuity, according to Quantiles of Nutrient Intake, in the Age-Related Eye Diseases Studies 1 and 2.

	AREDS (n = 657 eyes of 508 participants)			AREDS2 (n = 1179 eyes of 867 participants)		
Nutrient	Estimate, ETDRS letters/year (CI)	Pairwise P Value	Interaction P Value	Estimate, ETDRS letters/year (CI)	Pairwise P Value	Interaction P Value
Vitamin A, IU						
Tertile 1	3.0 (2.7,3.3)	.	0.0005	2.4 (2.1,2.8)	.	0.0372
Tertile 2	3.3 (2.9,3.7)	0.2809	.	2.2 (1.9,2.6)	0.3827	.
Tertile 3	2.3 (1.9,2.6)	0.0031	.	1.8 (1.4,2.2)	0.0115	.
Vitamin A, RAE						
Tertile 1	.	.	.	2.5 (2.1,2.8)	.	0.0021
Tertile 2	.	.	.	2.3 (2.0,2.7)	0.5502	.
Tertile 3	.	.	.	1.6 (1.3,2.0)	0.0009	.
Retinol, mcg						
Tertile 1	2.7 (2.3,3.1)	.	0.3592	2.8 (2.4,3.1)	.	<.0001
Tertile 2	3.1 (2.7,3.4)	0.1552	.	1.5 (1.2,1.9)	<.0001	.
Tertile 3	2.9 (2.5,3.2)	0.5819	.	2.3 (2.0,2.7)	0.0950	.
Vitamin D, mcg						
Tertile 1	2.8 (2.4,3.1)	.	0.6761	2.5 (2.2,2.9)	.	0.0182
Tertile 2	3.0 (2.6,3.3)	0.4360	.	1.8 (1.5,2.2)	0.0047	.
Tertile 3	2.9 (2.6,3.3)	0.4596	.	2.2 (1.8,2.5)	0.1344	.
Vitamin E, mg						
Tertile 1	2.6 (2.2,3.0)	.	0.0033	2.5 (2.2,2.9)	.	<.0001
Tertile 2	3.4 (3.0,3.8)	0.0026	.	2.4 (2.1,2.7)	0.5662	.
Tertile 3	2.7 (2.3,3.0)	0.8352	.	1.5 (1.1,1.9)	<.0001	.
Vitamin C, mg						
Tertile 1	3.3 (3.0,3.7)	.	0.0006	2.4 (2.0,2.7)	.	0.3039
Tertile 2	2.9 (2.6,3.3)	0.1122	.	2.2 (1.9,2.5)	0.4991	.
Tertile 3	2.3 (1.9,2.7)	0.0001	.	1.9 (1.6,2.3)	0.1240	.
Thiamine, mg						
Tertile 1	3.1 (2.8,3.5)	.	0.0051	2.1 (1.8,2.5)	.	0.7024
Tertile 2	3.1 (2.7,3.5)	0.9853	.	2.1 (1.7,2.4)	0.8181	.
Tertile 3	2.4 (2.0,2.8)	0.0031	.	2.3 (1.9,2.6)	0.5495	.
Riboflavin, mg						
Tertile 1	2.9 (2.6,3.3)	.	0.3766	2.2 (1.9,2.5)	.	0.7861
Tertile 2	3.0 (2.6,3.4)	0.7771	.	2.1 (1.7,2.4)	0.6147	.
Tertile 3	2.7 (2.3,3.0)	0.2780	.	2.2 (1.9,2.6)	0.8623	.
Niacin, mg						
Tertile 1	3.0 (2.6,3.3)	.	0.5917	2.3 (2.0,2.7)	.	0.2841
Tertile 2	2.9 (2.5,3.3)	0.7231	.	2.2 (1.9,2.6)	0.6126	.
Tertile 3	2.7 (2.4,3.1)	0.3090	.	1.9 (1.6,2.3)	0.1173	.
Vitamin B6, mg						
Tertile 1	2.9 (2.5,3.2)	.	0.1531	2.4 (2.1,2.8)	.	0.1971
Tertile 2	3.2 (2.8,3.5)	0.2797	.	2.0 (1.7,2.4)	0.1144	.
Tertile 3	2.6 (2.2,3.0)	0.3402	.	2.0 (1.7,2.4)	0.1212	.
Folate, mcg						
Tertile 1	3.1 (2.8,3.5)	.	0.0372	2.3 (1.9,2.6)	.	0.0011
Tertile 2	2.9 (2.5,3.3)	0.3413	.	2.5 (2.2,2.9)	0.2993	.
Tertile 3	2.5 (2.1,2.9)	0.0104	.	1.6 (1.2,2.0)	0.0101	.
Natural Food Folate						
Tertile 1	.	.	.	2.3 (2.0,2.7)	.	0.0018
Tertile 2	.	.	.	2.5 (2.2,2.9)	0.4273	.
Tertile 3	.	.	.	1.6 (1.3,2.0)	0.0089	.

Folic Acid						
Tertile 1	.	.	.	3.0 (2.6,3.3)	.	<.0001
Tertile 2	.	.	.	1.9 (1.5,2.2)	<.0001	.
Tertile 3	.	.	.	1.7 (1.3,2.0)	<.0001	.
Vitamin B12, mcg						
Tertile 1	2.5 (2.1,2.9)	.	0.0120	2.3 (1.9,2.6)	.	0.8341
Tertile 2	3.3 (2.9,3.6)	0.0041	.	2.1 (1.7,2.4)	0.5470	.
Tertile 3	2.7 (2.4,3.1)	0.3946	.	2.2 (1.8,2.5)	0.7613	.
Beta-carotene, mcg						
Tertile 1	3.0 (2.7,3.4)	.	0.2090	2.4 (2.0,2.7)	.	0.1123
Tertile 2	3.0 (2.6,3.3)	0.7635	.	2.2 (1.9,2.6)	0.5259	.
Tertile 3	2.6 (2.2,3.0)	0.0879	.	1.9 (1.5,2.2)	0.0401	.
Beta-carotene equivalents, mcg						
Tertile 1	3.0 (2.6,3.3)	.	0.2497	.	.	.
Tertile 2	3.0 (2.6,3.4)	0.7795	.	.	.	.
Tertile 3	2.6 (2.2,3.0)	0.1718	.	.	.	.
Alpha-carotene, mcg						
Tertile 1	2.8 (2.5,3.2)	.	0.0767	2.6 (2.2,2.9)	.	0.0003
Tertile 2	3.2 (2.8,3.6)	0.1576	.	2.3 (2.0,2.6)	0.2489	.
Tertile 3	2.6 (2.2,3.0)	0.4114	.	1.5 (1.1,1.9)	<.0001	.
Beta-cryptoxanthin, mcg						
Tertile 1	2.9 (2.6,3.2)	.	0.3823	2.4 (2.0,2.7)	.	0.4816
Tertile 2	3.1 (2.7,3.4)	0.5582	.	2.1 (1.7,2.4)	0.2431	.
Tertile 3	2.7 (2.3,3.1)	0.3792	.	2.1 (1.8,2.5)	0.3724	.
Lutein and zeaxanthin, mcg						
Tertile 1	2.8 (2.4,3.1)	.	0.0044	2.4 (2.1,2.8)	.	0.0599
Tertile 2	3.3 (2.9,3.6)	0.0296	.	1.8 (1.5,2.2)	0.0182	.
Tertile 3	2.4 (1.9,2.8)	0.1566	.	2.2 (1.9,2.6)	0.3480	.
Lycopene, mcg						
Tertile 1	3.0 (2.7,3.4)	.	0.4478	2.2 (1.8,2.6)	.	0.4167
Tertile 2	2.9 (2.5,3.3)	0.6411	.	2.0 (1.7,2.3)	0.3861	.
Tertile 3	2.7 (2.3,3.1)	0.2075	.	2.3 (2.0,2.7)	0.7130	.
Calcium, mg						
Tertile 1	3.1 (2.8,3.5)	.	0.1870	2.7 (2.4,3.0)	.	0.0007
Tertile 2	2.6 (2.3,3.0)	0.0672	.	2.0 (1.7,2.4)	0.0054	.
Tertile 3	2.9 (2.5,3.3)	0.3492	.	1.8 (1.4,2.1)	0.0003	.
Magnesium, mg						
Tertile 1	2.9 (2.5,3.2)	.	0.4490	2.5 (2.1,2.8)	.	0.1516
Tertile 2	3.1 (2.7,3.4)	0.4365	.	2.0 (1.7,2.3)	0.0566	.
Tertile 3	2.7 (2.3,3.1)	0.5678	.	2.1 (1.8,2.5)	0.1885	.
Iron, mg						
Tertile 1	2.9 (2.6,3.3)	.	0.8955	2.6 (2.3,3.0)	.	0.0100
Tertile 2	2.9 (2.5,3.3)	0.9939	.	1.9 (1.6,2.3)	0.0052	.
Tertile 3	2.8 (2.4,3.2)	0.6749	.	2.0 (1.6,2.4)	0.0161	.
Zinc, mg						
Tertile 1	2.7 (2.3,3.0)	.	0.4157	2.1 (1.8,2.4)	.	0.4710
Tertile 2	3.0 (2.6,3.4)	0.2653	.	2.4 (2.0,2.7)	0.3234	.
Tertile 3	3.0 (2.6,3.4)	0.2481	.	2.1 (1.7,2.4)	0.8746	.
Copper, mg						
Tertile 1	3.1 (2.8,3.5)	.	0.2460	2.2 (1.9,2.6)	.	0.0229
Tertile 2	2.8 (2.4,3.1)	0.1648	.	2.5 (2.1,2.8)	0.3896	.
Tertile 3	2.7 (2.3,3.1)	0.1445	.	1.8 (1.4,2.1)	0.0801	.
Selenium, mcg						
Tertile 1	2.8 (2.4,3.1)	.	0.5632	2.5 (2.2,2.7)	.	0.0034

Tertile 2	2.9 (2.5,3.2)	0.5907	.	1.6 (0.8,2.4)	0.0365	.
Tertile 3	3.0 (2.6,3.4)	0.2842	.	1.8 (1.5,2.1)	0.0026	.
Saturated fat, % kcal						
Tertile 1	2.5 (2.1,2.9)	.	0.0350	1.7 (1.4,2.1)	.	0.0203
Tertile 2	2.8 (2.4,3.2)	0.3317	.	2.4 (2.1,2.7)	0.0086	.
Tertile 3	3.2 (2.9,3.6)	0.0110	.	2.3 (2.0,2.7)	0.0263	.
Monounsaturated fat, % kcal						
Tertile 1	2.5 (2.1,2.9)	.	0.0260	2.3 (1.9,2.6)	.	0.7883
Tertile 2	3.2 (2.8,3.6)	0.0071	.	2.1 (1.7,2.4)	0.4905	.
Tertile 3	2.9 (2.6,3.2)	0.0927	.	2.2 (1.8,2.5)	0.7372	.
Cholesterol, mg						
Tertile 1	2.4 (2.0,2.8)	.	0.0043	2.1 (1.7,2.5)	.	0.3947
Tertile 2	3.1 (2.7,3.4)	0.0124	.	2.0 (1.7,2.4)	0.8209	.
Tertile 3	3.2 (2.8,3.6)	0.0019	.	2.4 (2.0,2.7)	0.3003	.
Oleic acid, mg per 1000 kcal						
Tertile 1	2.5 (2.1,2.9)	.	0.1142	2.2 (1.9,2.6)	.	0.8502
Tertile 2	3.1 (2.7,3.5)	0.0449	.	2.2 (1.9,2.5)	0.8953	.
Tertile 3	3.0 (2.6,3.3)	0.1098	.	2.1 (1.7,2.4)	0.5873	.
Linoleic acid, mg per 1000 kcal						
Tertile 1	2.5 (2.1,2.9)	.	0.1364	2.3 (2.0,2.7)	.	0.1746
Tertile 2	3.0 (2.7,3.4)	0.0743	.	2.3 (2.0,2.7)	0.9398	.
Tertile 3	3.0 (2.7,3.3)	0.0803	.	1.9 (1.6,2.3)	0.1090	.
a-Linolenic acid, mg per 1000 kcal						
Tertile 1	2.5 (2.1,2.9)	.	0.0507	2.5 (2.1,2.8)	.	0.0352
Tertile 2	2.9 (2.6,3.3)	0.1150	.	2.3 (1.9,2.7)	0.4991	.
Tertile 3	3.1 (2.8,3.5)	0.0159	.	1.9 (1.5,2.2)	0.0131	.
EPA, mg per 1000 kcal						
Tertile 1	2.9 (2.5,3.2)	.	0.6310	2.4 (2.1,2.8)	.	0.2049
Tertile 2	3.0 (2.6,3.4)	0.5719	.	2.1 (1.8,2.5)	0.2410	.
Tertile 3	2.7 (2.3,3.1)	0.6522	.	2.0 (1.6,2.3)	0.0816	.
DHA, mg per 1000 kcal						
Tertile 1	3.0 (2.7,3.4)	.	0.2284	2.5 (2.1,2.8)	.	0.0002
Tertile 2	3.0 (2.6,3.3)	0.8089	.	2.4 (2.1,2.7)	0.6850	.
Tertile 3	2.6 (2.2,3.0)	0.1056	.	1.5 (1.1,1.9)	0.0002	.
DPA, mg per 1000 kcal						
Tertile 1	.	.	.	2.6 (2.2,2.9)	.	0.0004
Tertile 2	.	.	.	2.3 (2.0,2.7)	0.3309	.
Tertile 3	.	.	.	1.6 (1.2,1.9)	0.0001	.
EPA+DHA, mg per 1000 kcal						
Tertile 1	2.9 (2.6,3.3)	.	0.6983	2.5 (2.2,2.9)	.	0.0297
Tertile 2	2.9 (2.6,3.3)	0.9312	.	2.2 (1.8,2.5)	0.1508	.
Tertile 3	2.7 (2.3,3.1)	0.4732	.	1.8 (1.5,2.2)	0.0081	.
EPA+DPA+DHA, mg per 1000 kcal						
Tertile 1	.	.	.	2.4 (2.0,2.7)	.	0.2519
Tertile 2	.	.	.	2.2 (1.8,2.5)	0.3603	.
Tertile 3	.	.	.	1.9 (1.5,2.3)	0.0983	.
Arachidonic acid, mg per 1000 kcal						
Tertile 1	2.8 (2.4,3.2)	.	0.1514	2.0 (1.7,2.4)	.	0.2015
Tertile 2	2.7 (2.3,3.0)	0.6186	.	2.4 (2.1,2.8)	0.1090	.
Tertile 3	3.2 (2.8,3.5)	0.1556	.	2.0 (1.7,2.4)	0.9256	.
Galactose, gm						
Tertile 1	3.1 (2.8,3.5)	.	0.0993	.	.	.
Tertile 2	2.9 (2.5,3.3)	0.3929	.	.	.	.
Tertile 3	2.5 (2.1,2.9)	0.0318	.	.	.	.

Lactose, gm						
Tertile 1	3.0 (2.7,3.4)	.	0.5021	2.4 (2.0,2.8)	.	0.0026
Tertile 2	2.7 (2.4,3.1)	0.2479	.	1.7 (1.4,2.0)	0.0064	.
Tertile 3	2.9 (2.5,3.2)	0.4631	.	2.5 (2.1,2.8)	0.7665	.
Alcohol, gm						
Tertile 1	3.0 (2.6,3.3)	.	0.3558	2.4 (2.0,2.7)	.	0.2869
Tertile 2	2.6 (2.2,3.0)	0.2309	.	2.0 (1.6,2.3)	0.1225	.
Tertile 3	3.0 (2.6,3.4)	0.7927	.	2.2 (1.9,2.6)	0.6093	.
Soluble Dietary Fiber, gm						
Tertile 1	3.1 (2.7,3.4)	.	0.2191	.	.	.
Tertile 2	2.9 (2.6,3.2)	0.5059	.	.	.	.
Tertile 3	2.6 (2.1,3.0)	0.0825	.	.	.	.
Insoluble Dietary Fiber, gm						
Tertile 1	2.9 (2.6,3.2)	.	0.9772	.	.	.
Tertile 2	2.9 (2.5,3.2)	0.8796	.	.	.	.
Tertile 3	2.9 (2.5,3.3)	0.9428	.	.	.	.
Fiber (Soluble or Insoluble), gm						
Tertile 1	.	.	.	2.4 (2.1,2.8)	.	0.0654
Tertile 2	.	.	.	2.3 (1.9,2.6)	0.5743	.
Tertile 3	.	.	.	1.8 (1.5,2.2)	0.0251	.
Total Choline						
Tertile 1	.	.	.	2.4 (2.1,2.7)	.	0.0062
Tertile 2	.	.	.	1.7 (1.3,2.1)	0.0049	.
Tertile 3	.	.	.	2.4 (2.0,2.8)	0.9944	.
Free Choline						
Tertile 1	.	.	.	2.4 (2.0,2.7)	.	0.3452
Tertile 2	.	.	.	2.1 (1.7,2.4)	0.1900	.
Tertile 3	.	.	.	2.1 (1.7,2.4)	0.2369	.
Glycemic Index						
Tertile 1	.	.	.	2.5 (2.1,2.8)	.	0.0702
Tertile 2	.	.	.	2.1 (1.7,2.4)	0.0871	.
Tertile 3	.	.	.	1.9 (1.6,2.3)	0.0296	.
Glycemic Load						
Tertile 1	.	.	.	2.2 (1.8,2.5)	.	0.2501
Tertile 2	.	.	.	2.4 (2.0,2.7)	0.4207	.
Tertile 3	.	.	.	2.0 (1.6,2.3)	0.3860	.

Abbreviations: AREDS=Age-Related Eye Diseases Study; CI=confidence interval; DHA=docosahexaenoic acid; DPA=docosapentaenoic acid; ETDRS=Early Treatment Diabetic Retinopathy Study; EPA=eicosapentaenoic acid; IU=international units; RAE=retinol activity

Table S12. Rates of Decline in Best-Corrected Visual Acuity, according to Quantiles of the Alternative Mediterranean Dietary Index and its Components, in the Proximity Study Population of the Age-Related Eye Diseases Studies 1 and 2.

	AREDS (n = 390 eyes of 328 participants)			AREDS2 (n = 826 eyes of 652 participants)		
	Estimate, ETDRS letters/year (CI)	Pairwise P Value	Interaction P Value	Estimate, ETDRS letters/year (CI)	Pairwise P Value	Interaction P Value
aMedi						
Tertile 1	3.3 (2.8,3.7)	.	0.3730	2.4 (2.0,2.8)	.	0.0628
Tertile 2	2.8 (2.3,3.3)	0.1628	.	2.2 (1.8,2.6)	0.4908	.
Tertile 3	3.0 (2.5,3.6)	0.4643	.	1.8 (1.4,2.2)	0.0209	.
Whole fruit						
Quartile 1	3.3 (2.8,3.8)	.	0.4333	2.7 (2.2,3.2)	.	<.0001
Quartile 2	3.2 (2.6,3.7)	0.7470	.	2.4 (2.0,2.8)	0.3876	.
Quartile 3	3.0 (2.4,3.5)	0.4341	.	2.3 (1.8,2.7)	0.2033	.
Quartile 4	2.7 (2.1,3.2)	0.1163	.	1.1 (0.7,1.6)	<.0001	.
Vegetables						
Quartile 1	3.3 (2.9,3.8)	.	0.0321	2.3 (1.8,2.7)	.	0.4262
Quartile 2	2.4 (1.8,2.9)	0.0110	.	2.1 (1.7,2.5)	0.5893	.
Quartile 3	3.0 (2.4,3.5)	0.3276	.	2.4 (2.0,2.8)	0.6645	.
Quartile 4	3.5 (2.9,4.1)	0.7291	.	1.9 (1.4,2.4)	0.2670	.
Whole grains						
Quartile 1	3.5 (2.9,4.0)	.	0.2443	2.4 (1.9,2.9)	.	0.6262
Quartile 2	2.7 (2.1,3.3)	0.0476	.	2.0 (1.5,2.4)	0.2027	.
Quartile 3	3.0 (2.5,3.5)	0.1974	.	2.2 (1.8,2.6)	0.5660	.
Quartile 4	3.0 (2.4,3.5)	0.1778	.	2.1 (1.6,2.6)	0.3764	.
Nuts						
Quartile 1	3.3 (2.8,3.8)	.	0.6851	2.4 (1.9,2.8)	.	0.6334
Quartile 2	3.0 (2.4,3.6)	0.4904	.	1.9 (1.5,2.4)	0.1917	.
Quartile 3	2.8 (2.3,3.4)	0.2274	.	2.1 (1.7,2.6)	0.5054	.
Quartile 4	3.0 (2.5,3.6)	0.5406	.	2.2 (1.7,2.6)	0.5859	.
Legumes						
Quartile 1	3.0 (2.5,3.6)	.	0.3875	2.2 (1.6,2.7)	.	0.0343
Quartile 2	3.2 (2.7,3.7)	0.6201	.	1.6 (1.1,2.0)	0.0885	.
Quartile 3	2.7 (2.2,3.2)	0.3913	.	2.3 (1.9,2.7)	0.6734	.
Quartile 4	3.3 (2.7,3.9)	0.4834	.	2.5 (2.0,2.9)	0.4590	.
Red meat*						
Quartile 1	2.9 (2.4,3.4)	.	0.6969	3.0 (2.6,3.4)	.	<.0001
Quartile 2	3.2 (2.7,3.7)	0.4533	.	2.1 (1.7,2.6)	0.0037	.
Quartile 3	3.2 (2.6,3.8)	0.5147	.	1.5 (1.0,2.0)	<.0001	.
Quartile 4	2.8 (2.2,3.4)	0.7371	.	1.7 (1.2,2.1)	<.0001	.
Fish						
Quartile 1	2.9 (2.3,3.4)	.	<.0001	2.1 (1.5,2.7)	.	0.5123
Quartile 2	4.0 (3.5,4.5)	0.0016	.	2.3 (1.9,2.7)	0.5606	.
Quartile 3	2.2 (1.7,2.8)	0.1198	.	2.2 (1.8,2.6)	0.7769	.
Quartile 4	2.6 (1.9,3.2)	0.4714	.	1.8 (1.2,2.3)	0.4633	.
MUFA: SFA						
Quartile 1	3.4 (2.9,4.0)	.	0.3528	2.5 (2.1,3.0)	.	0.1835
Quartile 2	2.9 (2.3,3.4)	0.1520	.	2.1 (1.7,2.5)	0.1570	.
Quartile 3	3.0 (2.5,3.6)	0.3071	.	2.2 (1.8,2.7)	0.4162	.
Quartile 4	2.8 (2.2,3.3)	0.0950	.	1.8 (1.3,2.3)	0.0327	.
Alcohol						
In interval	3.0 (2.7,3.3)	.	0.8138	2.6 (2.0,3.2)	.	.

Not in interval	3.1 (2.3,4.0)	0.8138	.	2.1 (1.9,2.3)	0.1506	0.1506

Abbreviations: aMedi=Alternative Mediterranean Diet Index; AREDS=Age-Related Eye Diseases Study; CI=confidence interval; ETDRS=Early Treatment Diabetic Retinopathy Study; MUFA: SFA=monounsaturated fatty acid: saturated fatty acid

* For red meat, higher quartiles refer to lower levels of intake, which is more adherent to the Alternative Mediterranean Diet Index

Table S13. Rates of Decline in Best-Corrected Visual Acuity, according to Quantiles of Nutrient Intake, in the Proximity Study Population of the Age-Related Eye Diseases Studies 1 and 2.

	AREDS (n = 390 eyes of 328 participants)			AREDS2 (n = 826 eyes of 652 participants)		
Nutrient	Estimate, ETDRS letters/year (CI)	Pairwise P Value	Interaction P Value	Estimate, ETDRS letters/year (CI)	Pairwise P Value	Interaction P Value
Vitamin A, IU						
Tertile 1	3.1 (2.7,3.6)	.	0.0037	2.3 (1.9,2.7)	.	0.3487
Tertile 2	3.6 (3.1,4.1)	0.1889	.	2.3 (1.9,2.6)	0.9982	.
Tertile 3	2.4 (1.9,2.9)	0.0271	.	1.9 (1.5,2.3)	0.2125	.
Vitamin A, RAE						
Tertile 1	.	.	.	2.4 (2.0,2.7)	.	0.0030
Tertile 2	.	.	.	2.5 (2.1,2.9)	0.6144	.
Tertile 3	.	.	.	1.6 (1.2,2.0)	0.0070	.
Retinol, mcg						
Tertile 1	2.7 (2.2,3.2)	.	0.1282	2.6 (2.2,3.0)	.	0.0110
Tertile 2	3.0 (2.6,3.5)	0.3190	.	1.8 (1.4,2.1)	0.0027	.
Tertile 3	3.4 (2.9,3.9)	0.0428	.	2.2 (1.8,2.5)	0.1061	.
Vitamin D, mcg						
Tertile 1	3.0 (2.5,3.4)	.	0.1379	2.5 (2.1,2.9)	.	0.1118
Tertile 2	2.8 (2.3,3.2)	0.5594	.	1.9 (1.5,2.3)	0.0420	.
Tertile 3	3.4 (3.0,3.9)	0.1821	.	2.1 (1.7,2.5)	0.1449	.
Vitamin E, mg						
Tertile 1	2.9 (2.4,3.4)	.	0.3799	2.4 (2.1,2.8)	.	<.0001
Tertile 2	3.3 (2.8,3.8)	0.2540	.	2.7 (2.3,3.0)	0.4288	.
Tertile 3	2.9 (2.4,3.4)	0.9436	.	1.3 (0.9,1.7)	<.0001	.
Vitamin C, mg						
Tertile 1	3.2 (2.8,3.7)	.	0.0488	2.3 (1.9,2.7)	.	0.6346
Tertile 2	3.3 (2.8,3.8)	0.8273	.	2.2 (1.8,2.5)	0.6647	.
Tertile 3	2.5 (2.0,3.0)	0.0406	.	2.0 (1.6,2.4)	0.3416	.
Thiamine, mg						
Tertile 1	3.0 (2.6,3.5)	.	0.0491	2.2 (1.8,2.6)	.	0.1465
Tertile 2	3.5 (3.0,4.1)	0.1397	.	1.9 (1.5,2.2)	0.2188	.
Tertile 3	2.6 (2.2,3.1)	0.2427	.	2.4 (2.0,2.8)	0.4891	.
Riboflavin, mg						
Tertile 1	3.0 (2.6,3.5)	.	0.9680	2.2 (1.8,2.6)	.	0.4456
Tertile 2	3.1 (2.6,3.5)	0.8794	.	2.3 (1.9,2.7)	0.7054	.
Tertile 3	3.0 (2.5,3.5)	0.9181	.	1.9 (1.5,2.3)	0.3825	.
Niacin, mg						
Tertile 1	3.0 (2.5,3.4)	.	0.6279	2.1 (1.8,2.5)	.	0.1345
Tertile 2	3.2 (2.8,3.7)	0.4024	.	2.4 (2.1,2.8)	0.2694	.
Tertile 3	2.9 (2.4,3.4)	0.9664	.	1.9 (1.5,2.3)	0.3427	.
Vitamin B6, mg						
Tertile 1	2.7 (2.3,3.2)	.	0.0645	2.2 (1.8,2.6)	.	0.0872
Tertile 2	3.5 (3.0,4.0)	0.0204	.	2.4 (2.1,2.8)	0.3738	.
Tertile 3	3.0 (2.5,3.5)	0.4251	.	1.8 (1.4,2.2)	0.1943	.
Folate, mcg						
Tertile 1	3.2 (2.8,3.7)	.	0.3692	2.2 (1.8,2.6)	.	0.0001
Tertile 2	3.1 (2.6,3.5)	0.5560	.	2.6 (2.3,3.0)	0.1344	.
Tertile 3	2.8 (2.2,3.3)	0.1583	.	1.4 (1.0,1.8)	0.0075	.
Natural Food Folate						
Tertile 1	.	.	.	2.2 (1.8,2.6)	.	<.0001
Tertile 2	.	.	.	2.8 (2.4,3.1)	0.0429	.
Tertile 3	.	.	.	1.5 (1.1,1.9)	0.0120	.

Folic Acid						
Tertile 1	.	.	.	2.6 (2.3,3.0)	.	0.0072
Tertile 2	.	.	.	2.0 (1.6,2.4)	0.0227	.
Tertile 3	.	.	.	1.8 (1.4,2.2)	0.0027	.
Vitamin B12, mcg						
Tertile 1	2.4 (1.9,2.9)	.	0.0107	2.1 (1.8,2.5)	.	0.9597
Tertile 2	3.4 (3.0,3.9)	0.0029	.	2.2 (1.8,2.6)	0.8768	.
Tertile 3	3.1 (2.6,3.6)	0.0450	.	2.1 (1.7,2.5)	0.8964	.
Beta-carotene, mcg						
Tertile 1	3.3 (2.8,3.7)	.	0.4267	2.3 (1.9,2.7)	.	0.6726
Tertile 2	3.0 (2.5,3.5)	0.4657	.	2.1 (1.7,2.5)	0.5695	.
Tertile 3	2.8 (2.3,3.3)	0.1962	.	2.0 (1.6,2.4)	0.3807	.
Beta-carotene equivalents, mcg						
Tertile 1	3.2 (2.7,3.6)	.	0.6326	.	.	.
Tertile 2	3.1 (2.6,3.6)	0.7748	.	.	.	.
Tertile 3	2.8 (2.3,3.3)	0.3446	.	.	.	.
Alpha-carotene, mcg						
Tertile 1	3.0 (2.5,3.4)	.	0.1749	2.4 (2.0,2.8)	.	0.0015
Tertile 2	3.4 (2.9,3.9)	0.1973	.	2.4 (2.0,2.7)	0.7802	.
Tertile 3	2.8 (2.3,3.2)	0.6133	.	1.5 (1.0,1.9)	0.0012	.
Beta-cryptoxanthin, mcg						
Tertile 1	2.9 (2.5,3.4)	.	0.1173	2.6 (2.2,3.0)	.	0.0040
Tertile 2	3.5 (3.0,3.9)	0.1255	.	1.7 (1.3,2.0)	0.0012	.
Tertile 3	2.8 (2.3,3.2)	0.5949	.	2.3 (1.9,2.7)	0.2782	.
Lutein and zeaxanthin, mcg						
Tertile 1	3.0 (2.6,3.4)	.	0.0069	2.2 (1.8,2.6)	.	0.0721
Tertile 2	3.6 (3.1,4.0)	0.0799	.	1.8 (1.4,2.2)	0.1233	.
Tertile 3	2.4 (1.8,2.9)	0.0911	.	2.4 (2.0,2.8)	0.4603	.
Lycopene, mcg						
Tertile 1	3.3 (2.8,3.7)	.	0.5151	2.1 (1.7,2.6)	.	0.3600
Tertile 2	2.9 (2.5,3.4)	0.3452	.	2.0 (1.6,2.3)	0.5511	.
Tertile 3	2.9 (2.4,3.4)	0.3023	.	2.4 (2.0,2.7)	0.4587	.
Calcium, mg						
Tertile 1	3.3 (2.8,3.8)	.	0.4040	2.8 (2.4,3.2)	.	<.0001
Tertile 2	2.8 (2.4,3.3)	0.1790	.	2.0 (1.7,2.4)	0.0033	.
Tertile 3	3.0 (2.5,3.5)	0.4467	.	1.6 (1.1,2.0)	<.0001	.
Magnesium, mg						
Tertile 1	2.7 (2.2,3.1)	.	0.0217	2.6 (2.2,3.0)	.	0.0371
Tertile 2	3.6 (3.1,4.0)	0.0070	.	2.0 (1.7,2.4)	0.0440	.
Tertile 3	2.9 (2.4,3.4)	0.4860	.	1.9 (1.5,2.3)	0.0155	.
Iron, mg						
Tertile 1	3.1 (2.7,3.6)	.	0.2157	2.7 (2.4,3.1)	.	0.0008
Tertile 2	2.7 (2.2,3.2)	0.2078	.	1.8 (1.4,2.1)	0.0004	.
Tertile 3	3.3 (2.8,3.8)	0.6202	.	1.9 (1.6,2.3)	0.0047	.
Zinc, mg						
Tertile 1	2.6 (2.1,3.0)	.	0.0120	2.0 (1.6,2.4)	.	0.2836
Tertile 2	3.6 (3.1,4.1)	0.0030	.	2.4 (2.0,2.8)	0.1297	.
Tertile 3	3.0 (2.6,3.5)	0.1763	.	2.1 (1.7,2.5)	0.7237	.
Copper, mg						
Tertile 1	3.2 (2.7,3.7)	.	0.6365	2.1 (1.7,2.6)	.	0.0760
Tertile 2	2.9 (2.4,3.4)	0.3480	.	2.4 (2.1,2.8)	0.2959	.
Tertile 3	3.0 (2.5,3.5)	0.5680	.	1.8 (1.4,2.2)	0.2504	.
Selenium, mcg						
Tertile 1	2.8 (2.4,3.3)	.	0.2382	2.3 (2.0,2.6)	.	0.3164

Tertile 2	3.0 (2.5,3.4)	0.6496	.	2.3 (1.4,3.3)	0.8924	.
Tertile 3	3.4 (2.9,4.0)	0.0976	.	1.9 (1.6,2.3)	0.1456	.
Saturated fat, % kcal						
Tertile 1	2.9 (2.4,3.4)	.	0.5358	1.7 (1.3,2.1)	.	0.0255
Tertile 2	2.9 (2.4,3.4)	0.9932	.	2.1 (1.8,2.5)	0.1448	.
Tertile 3	3.3 (2.8,3.7)	0.3428	.	2.5 (2.1,2.9)	0.0068	.
Monounsaturated fat, % kcal						
Tertile 1	2.9 (2.4,3.3)	.	0.3959	2.2 (1.8,2.6)	.	0.3593
Tertile 2	3.3 (2.8,3.8)	0.1883	.	1.9 (1.6,2.3)	0.3113	.
Tertile 3	3.0 (2.5,3.4)	0.7073	.	2.3 (1.9,2.7)	0.7328	.
Cholesterol, mg						
Tertile 1	2.7 (2.2,3.1)	.	0.0430	1.9 (1.5,2.3)	.	0.1700
Tertile 2	3.0 (2.6,3.5)	0.2522	.	2.2 (1.8,2.6)	0.2169	.
Tertile 3	3.5 (3.0,4.0)	0.0122	.	2.4 (2.0,2.7)	0.0639	.
Oleic acid, mg per 1000 kcal						
Tertile 1	2.9 (2.4,3.4)	.	0.5899	2.3 (1.9,2.7)	.	0.6634
Tertile 2	3.2 (2.8,3.7)	0.3200	.	2.0 (1.6,2.4)	0.3760	.
Tertile 3	3.0 (2.5,3.5)	0.7637	.	2.2 (1.8,2.6)	0.7654	.
Linoleic acid, mg per 1000 kcal						
Tertile 1	2.6 (2.1,3.1)	.	0.0759	2.1 (1.7,2.5)	.	0.1657
Tertile 2	3.4 (2.9,3.9)	0.0269	.	2.4 (2.1,2.8)	0.2783	.
Tertile 3	3.1 (2.7,3.6)	0.1111	.	1.9 (1.6,2.3)	0.5017	.
a-Linolenic acid, mg per 1000 kcal						
Tertile 1	2.8 (2.3,3.2)	.	0.3685	2.3 (1.9,2.7)	.	0.1389
Tertile 2	3.2 (2.7,3.7)	0.2379	.	2.4 (1.9,2.8)	0.9470	.
Tertile 3	3.2 (2.7,3.6)	0.2034	.	1.9 (1.5,2.2)	0.0971	.
EPA, mg per 1000 kcal						
Tertile 1	3.0 (2.5,3.4)	.	0.1911	2.1 (1.7,2.5)	.	0.6956
Tertile 2	3.4 (2.9,3.8)	0.2020	.	2.1 (1.7,2.5)	0.9801	.
Tertile 3	2.7 (2.2,3.3)	0.5478	.	2.3 (1.9,2.7)	0.4710	.
DHA, mg per 1000 kcal						
Tertile 1	3.2 (2.7,3.7)	.	0.0933	2.1 (1.7,2.5)	.	0.0180
Tertile 2	3.2 (2.8,3.7)	0.9309	.	2.5 (2.1,2.8)	0.1765	.
Tertile 3	2.5 (2.0,3.1)	0.0589	.	1.7 (1.3,2.1)	0.1510	.
DPA, mg per 1000 kcal						
Tertile 1	.	.	.	2.3 (1.9,2.7)	.	0.1530
Tertile 2	.	.	.	2.3 (2.0,2.7)	0.8102	.
Tertile 3	.	.	.	1.8 (1.4,2.2)	0.1297	.
EPA+DHA, mg per 1000 kcal						
Tertile 1	3.1 (2.7,3.6)	.	0.5319	2.1 (1.7,2.5)	.	0.9829
Tertile 2	3.1 (2.7,3.6)	0.9434	.	2.1 (1.8,2.5)	0.9814	.
Tertile 3	2.8 (2.2,3.3)	0.3284	.	2.2 (1.8,2.6)	0.8658	.
EPA+DPA+DHA, mg per 1000 kcal						
Tertile 1	.	.	.	2.2 (1.8,2.6)	.	0.4840
Tertile 2	.	.	.	2.2 (1.9,2.6)	0.9373	.
Tertile 3	.	.	.	1.9 (1.5,2.4)	0.3078	.
Arachidonic acid, mg per 1000 kcal						
Tertile 1	3.1 (2.7,3.5)	.	0.0226	1.8 (1.4,2.2)	.	0.0153
Tertile 2	2.6 (2.1,3.0)	0.1036	.	2.6 (2.2,3.0)	0.0042	.
Tertile 3	3.5 (3.0,4.1)	0.1984	.	2.1 (1.7,2.5)	0.2937	.
Galactose, gm						
Tertile 1	3.4 (3.0,3.9)	.	0.0247	.	.	.
Tertile 2	3.0 (2.5,3.5)	0.2018	.	.	.	.
Tertile 3	2.5 (2.0,3.0)	0.0066	.	.	.	.

Lactose, gm						
Tertile 1	3.1 (2.7,3.6)	.	0.8684	2.3 (1.9,2.8)	.	0.4720
Tertile 2	3.0 (2.6,3.5)	0.7980	.	2.0 (1.6,2.4)	0.2209	.
Tertile 3	3.0 (2.5,3.4)	0.5955	.	2.1 (1.8,2.5)	0.4781	.
Alcohol, gm						
Tertile 1	3.1 (2.7,3.5)	.	0.0800	2.2 (1.8,2.6)	.	0.9488
Tertile 2	2.5 (2.0,3.1)	0.1014	.	2.1 (1.8,2.5)	0.7485	.
Tertile 3	3.4 (2.9,3.9)	0.3752	.	2.1 (1.7,2.6)	0.8340	.
Soluble Dietary Fiber, gm						
Tertile 1	3.1 (2.6,3.5)	.	0.8042	.	.	.
Tertile 2	3.1 (2.7,3.6)	0.8718	.	.	.	.
Tertile 3	2.9 (2.3,3.4)	0.6220	.	.	.	.
Insoluble Dietary Fiber, gm						
Tertile 1	3.0 (2.6,3.5)	.	0.7284	.	.	.
Tertile 2	3.2 (2.7,3.6)	0.6378	.	.	.	.
Tertile 3	2.9 (2.4,3.4)	0.7271	.	.	.	.
Fiber (Soluble or Insoluble), gm						
Tertile 1	.	.	.	2.5 (2.1,2.9)	.	0.0034
Tertile 2	.	.	.	2.4 (2.0,2.7)	0.7046	.
Tertile 3	.	.	.	1.6 (1.2,2.0)	0.0020	.
Total Choline						
Tertile 1	.	.	.	2.2 (1.8,2.6)	.	0.3944
Tertile 2	.	.	.	1.9 (1.5,2.3)	0.3233	.
Tertile 3	.	.	.	2.3 (1.9,2.7)	0.7229	.
Free Choline						
Tertile 1	.	.	.	2.4 (2.0,2.8)	.	0.2259
Tertile 2	.	.	.	2.1 (1.7,2.5)	0.2542	.
Tertile 3	.	.	.	1.9 (1.5,2.3)	0.0933	.
Glycemic Index						
Tertile 1	.	.	.	2.3 (1.9,2.7)	.	0.5565
Tertile 2	.	.	.	2.0 (1.6,2.4)	0.2790	.
Tertile 3	.	.	.	2.2 (1.7,2.6)	0.5957	.
Glycemic Load						
Tertile 1	.	.	.	2.2 (1.8,2.6)	.	0.6391
Tertile 2	.	.	.	2.3 (1.9,2.7)	0.8483	.
Tertile 3	.	.	.	2.0 (1.6,2.4)	0.4718	.

Abbreviations: AREDS=Age-Related Eye Diseases Study; CI=confidence interval; DHA=docosahexaenoic acid; DPA=docosapentaenoic acid; ETDRS=Early Treatment Diabetic Retinopathy Study; EPA=eicosapentaenoic acid; IU=international units; RAE=retinol activity equivalents

Figure S1. Geographic Atrophy Area-Based Progression Rates, according to Quantiles of Dietary Index or Intake, in the Proximity Study Population of the Age-Related Eye Diseases Study: (A) Alternative Mediterranean Diet Index and its Nine Components; (B) Nutrients.

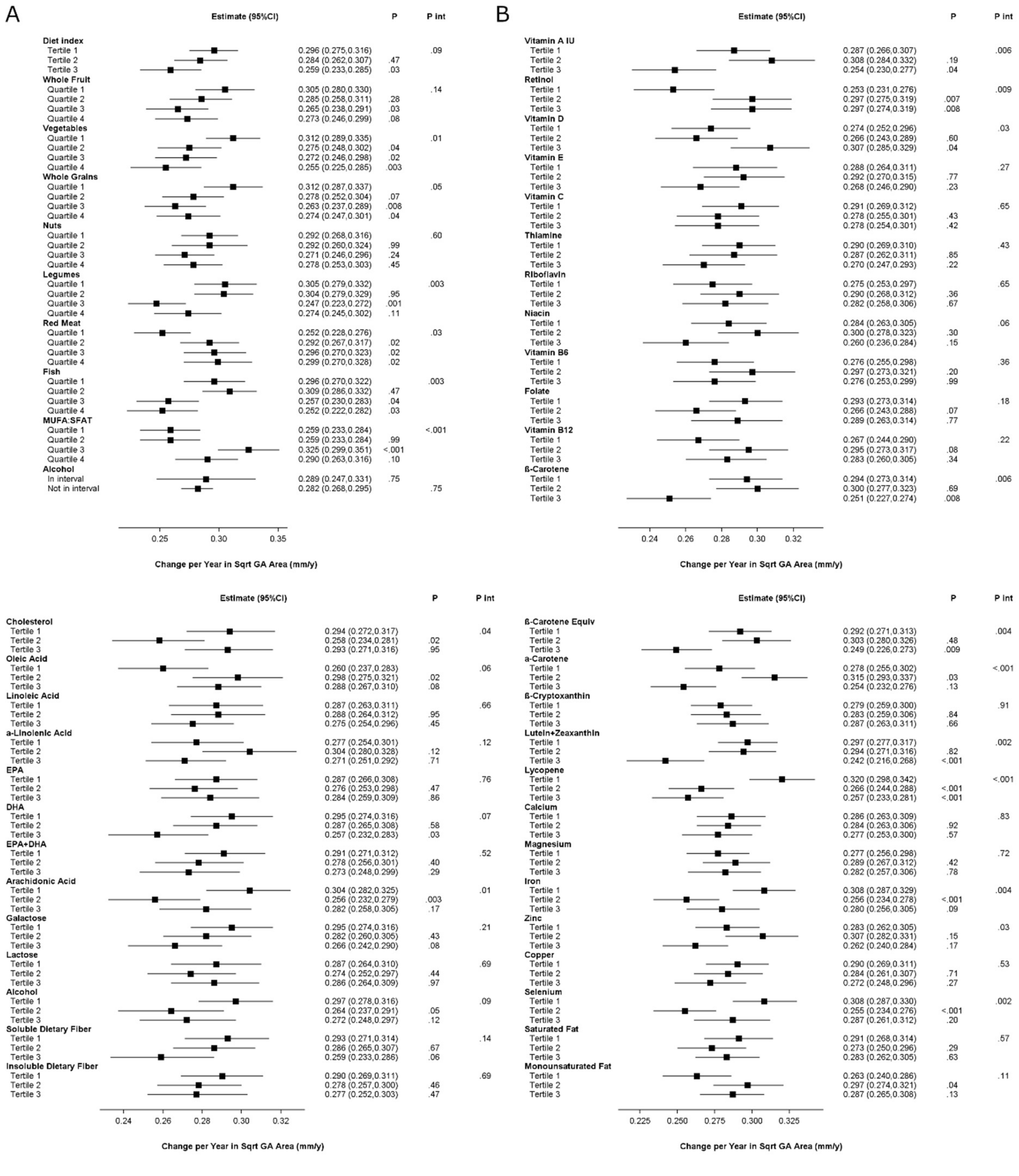

Abbreviations: CI=confidence interval; DHA=docosahexaenoic acid; DPA=docosapentaenoic acid; EPA=eicosapentaenoic acid; GA=geographic atrophy; IU=international units; MUFA: SFAT=monounsaturated fatty acid: saturated fatty acid ratio.

Figure S2. Geographic Atrophy Area-Based Progression Rates, according to Quantiles of Dietary Index or Intake, in the Proximity Study Population of the Age-Related Eye Diseases Study 2: (A) Alternative Mediterranean Diet Index and its Nine Components; (B) Nutrients.

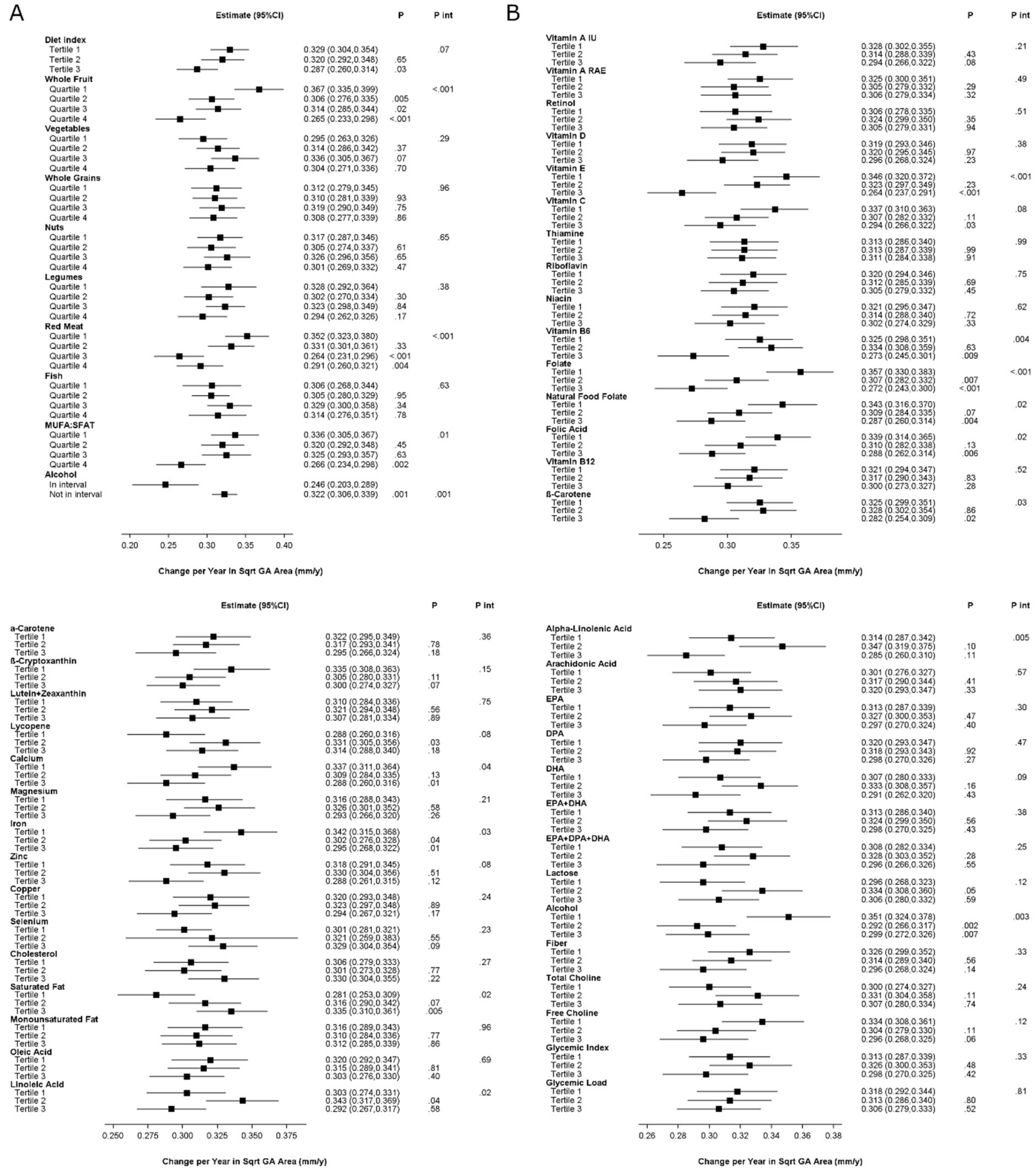

Abbreviations: CI=confidence interval; DHA=docosahexaenoic acid; DPA=docosapentaenoic acid; EPA=eicosapentaenoic acid; GA=geographic atrophy; IU=international units; MUFA: SFAT=monounsaturated fatty acid: saturated fatty acid ratio; RAE=retinol activity equivalents.

Figure S3. Geographic atrophy proximity-based progression rates, according to quantiles of dietary index or intake for the Alternative Mediterranean Diet Index and its nine components, in the Age-Related Eye Diseases Study: (A) in participants randomized to oral antioxidant supplementation; (B) in participants randomized to no oral antioxidant supplementation.

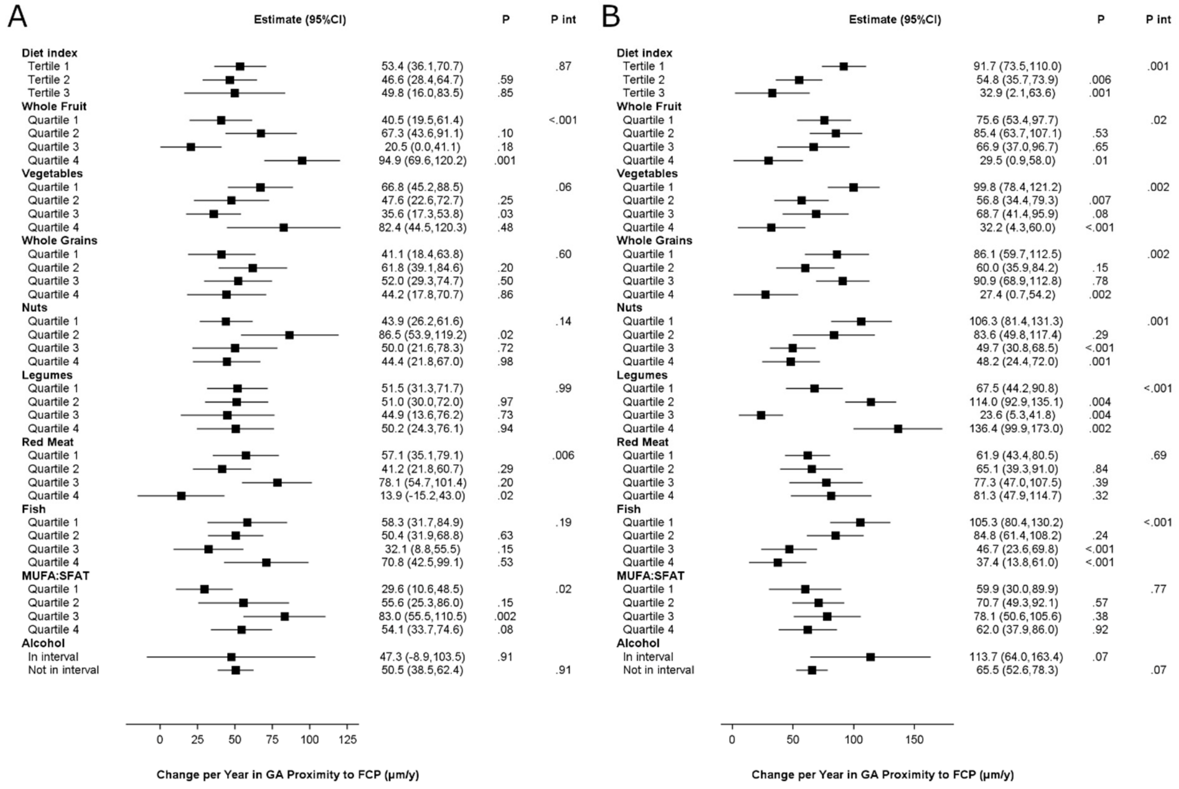

Higher quantiles indicate higher adherence to the Alternative Mediterranean Diet or higher intake of its components. The exceptions are red meat, where higher quantiles indicate lower intake, i.e., higher adherence to the Alternative Mediterranean Diet, and alcohol, where "in interval" refers to intake within the specified interval.

Abbreviations: CI=confidence interval; FCP=foveal center-point; GA=geographic atrophy; MUFA: SFAT=monounsaturated fatty acid: saturated fatty acid ratio.

Figure S4. Geographic atrophy proximity-based progression rates, according to quantiles of dietary index or intake for the Alternative Mediterranean Diet Index and its nine components, in the Age-Related Eye Diseases Study 2: (A) in participants randomized to oral lutein/zeaxanthin supplementation; (B) in participants randomized to no oral lutein/zeaxanthin supplementation.

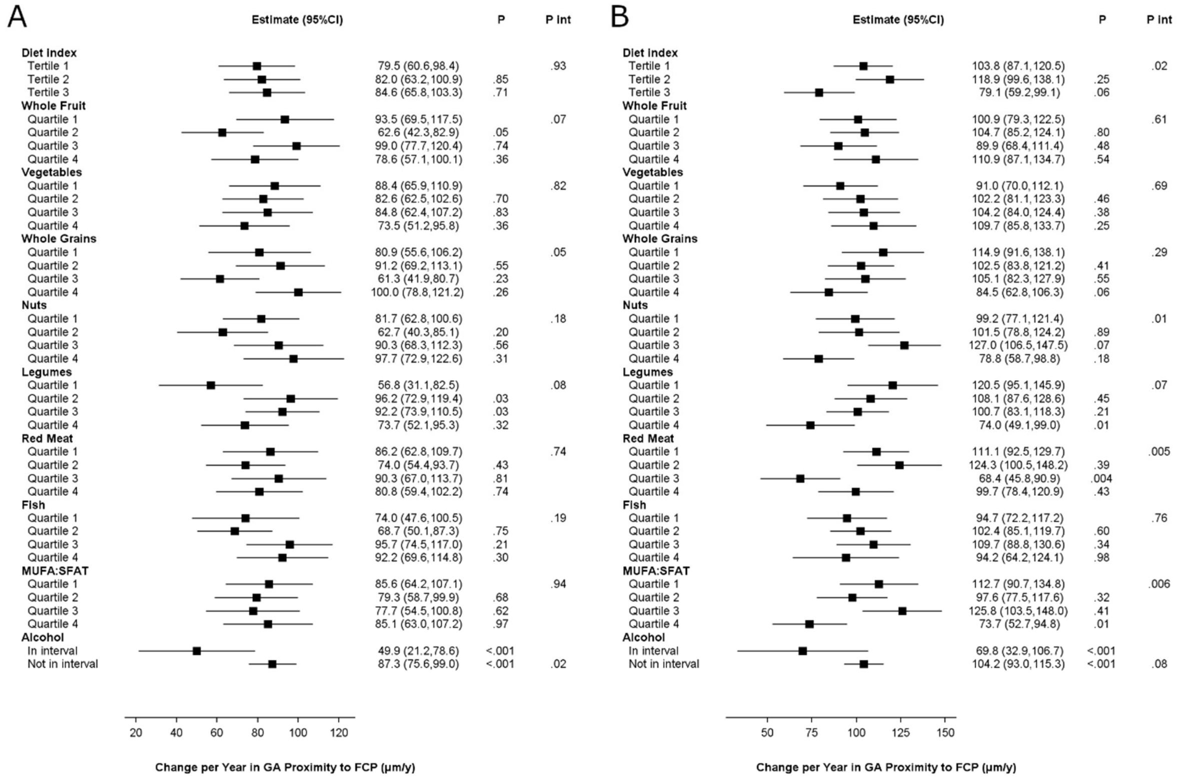

Higher quantiles indicate higher adherence to the Alternative Mediterranean Diet or higher intake of its components. The exceptions are red meat, where higher quantiles indicate lower intake, i.e., higher adherence to the Alternative Mediterranean Diet, and alcohol, where "in interval" refers to intake within the specified interval.

Abbreviations: CI=confidence interval; FCP=foveal center-point; GA=geographic atrophy; MUFA: SFAT=monounsaturated fatty acid: saturated fatty acid ratio.

Figure S5. Rates of Decline in Best-Corrected Visual Acuity, according to Quantiles of Dietary Index or Intake, in the Age-Related Eye Diseases Study: (A) Alternative Mediterranean Diet Index and its Nine Components; (B) Nutrients.

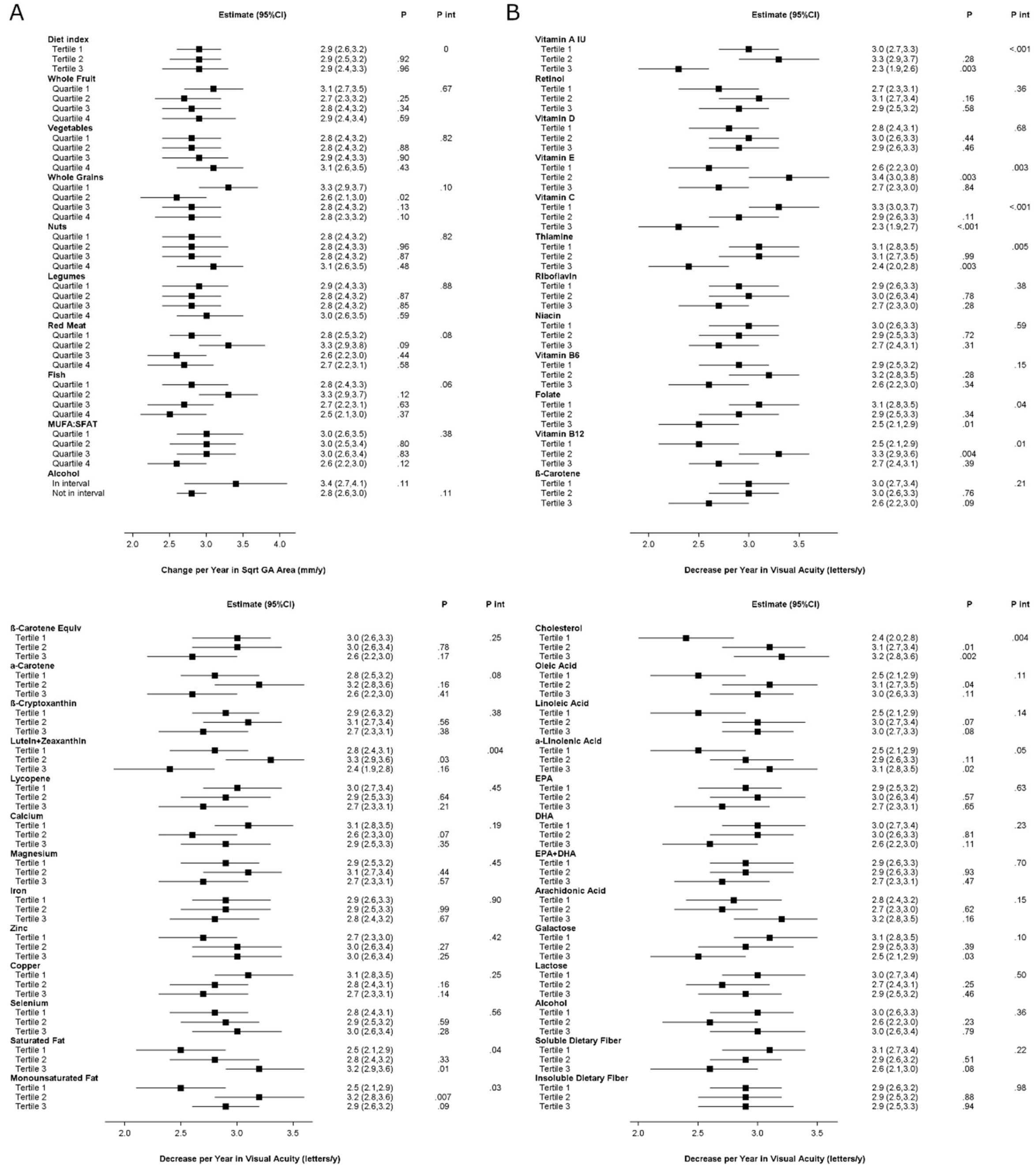

Abbreviations: CI=confidence interval; DHA=docosahexaenoic acid; DPA=docosapentaenoic acid; EPA=eicosapentaenoic acid; GA=geographic atrophy; IU=international units; MUFA: SFAT=monounsaturated fatty acid: saturated fatty acid ratio.

Figure S6. Rates of Decline in Best-Corrected Visual Acuity, according to Quantiles of Dietary Index or Intake, in the Age-Related Eye Diseases Study 2: (A) Alternative Mediterranean Diet Index and its Nine Components; (B) Nutrients.

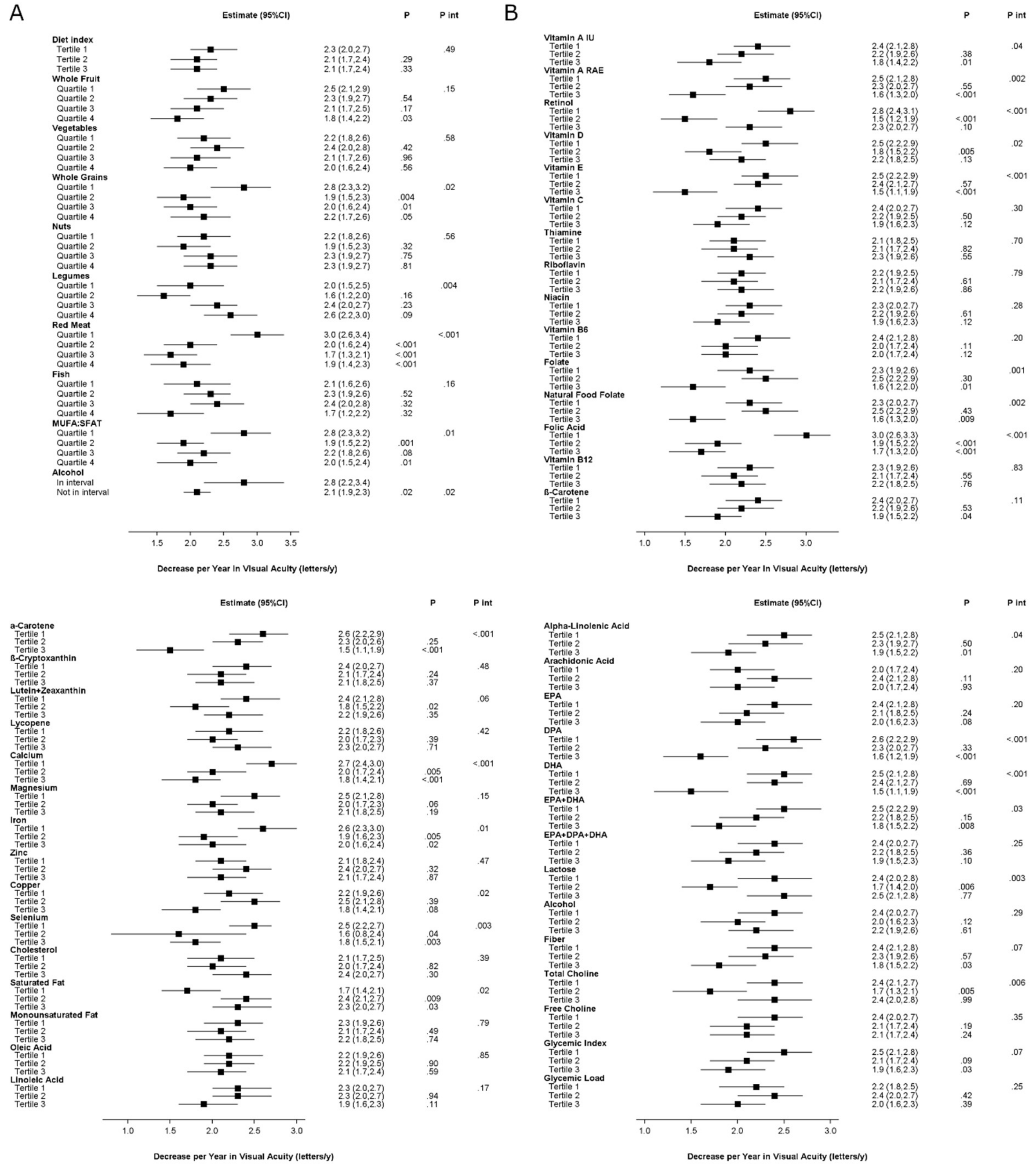

Abbreviations: CI=confidence interval; DHA=docosahexaenoic acid; DPA=docosapentaenoic acid; EPA=eicosapentaenoic acid; GA=geographic atrophy; IU=international units; MUFA: SFAT=monounsaturated fatty acid: saturated fatty acid ratio; RAE=retinol activity equivalents.

Figure S7. Rates of Decline in Best-Corrected Visual Acuity, according to Quantiles of Dietary Index or Intake, in the Proximity Study Population of the Age-Related Eye Diseases Study: (A) Alternative Mediterranean Diet Index and its Nine Components; (B) Nutrients.

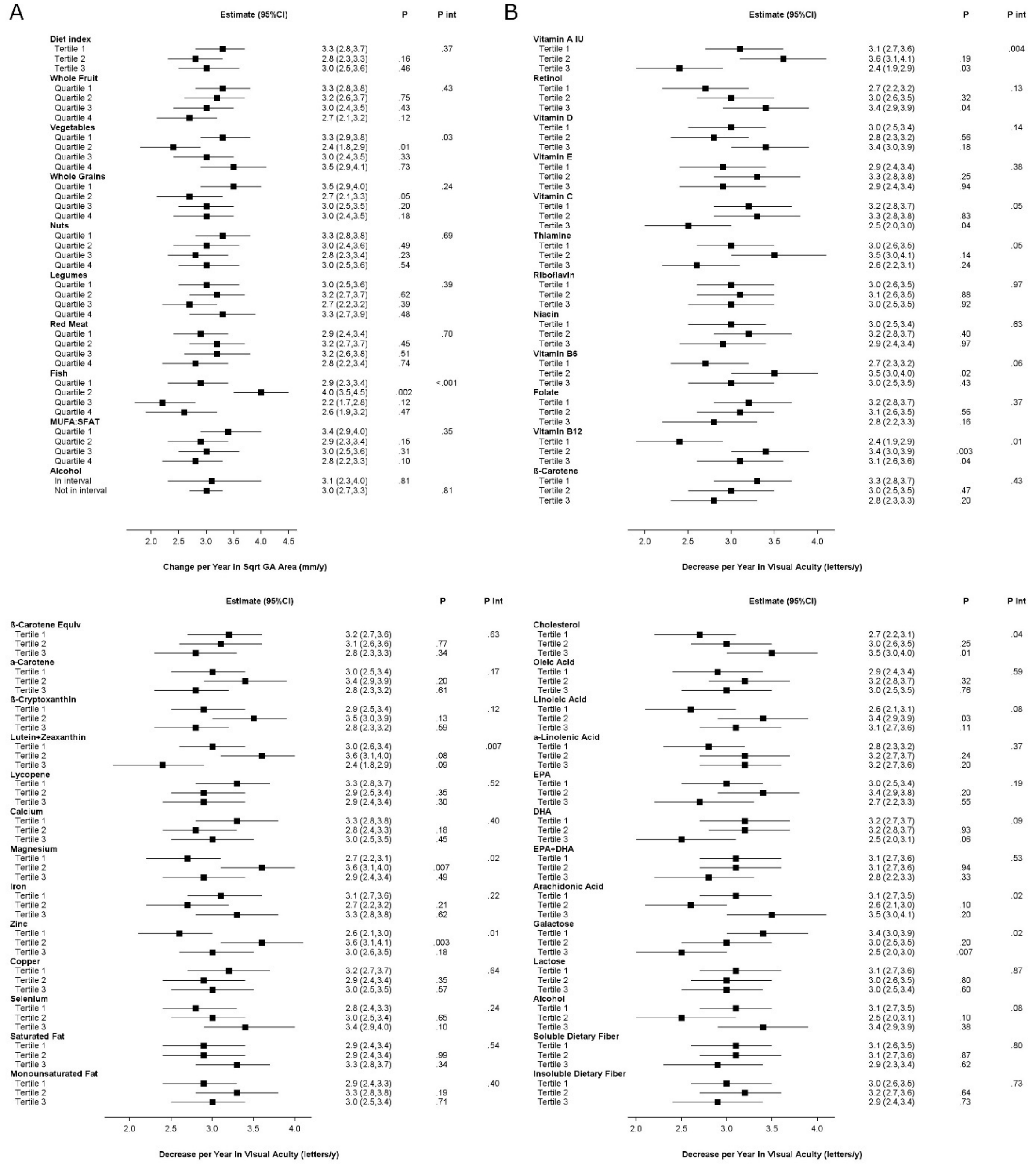

Abbreviations: CI=confidence interval; DHA=docosahexaenoic acid; DPA=docosapentaenoic acid; EPA=eicosapentaenoic acid; GA=geographic atrophy; IU=international units; MUFA: SFAT=monounsaturated fatty acid: saturated fatty acid ratio.

Figure S8. Rates of Decline in Best-Corrected Visual Acuity, according to Quantiles of Dietary Index or Intake, in the Proximity Study Population of the Age-Related Eye Diseases Study 2: (A) Alternative Mediterranean Diet Index and its Nine Components; (B) Nutrients.

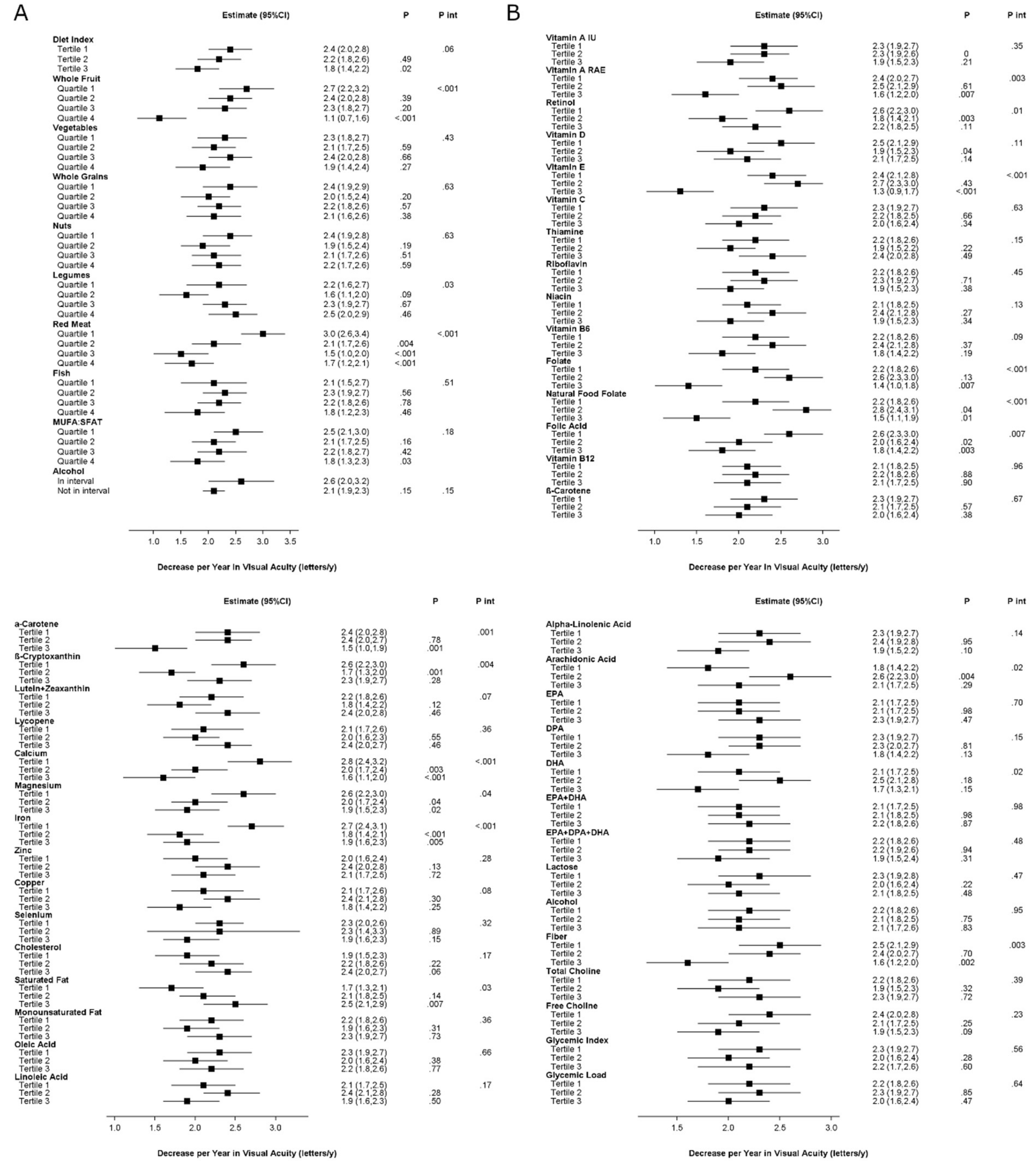

Abbreviations: CI=confidence interval; DHA=docosahexaenoic acid; DPA=docosapentaenoic acid; EPA=eicosapentaenoic acid; GA=geographic atrophy; IU=international units; MUFA: SFAT=monounsaturated fatty acid: saturated fatty acid ratio; RAE=retinol activity equivalents.
